# Multimodal substrate recruitment enables CTLH^MKLN1^ E3 ligase to target acetylated N-, C-, and internal degrons

**DOI:** 10.1038/s41467-026-78311-4

**Published:** 2026-10-17

**Authors:** Sara Sepic, Karthik V. Gottemukkala, Jiale Du, Samuel A. Maiwald, Jakub Chrustowicz, Eleftheria C. Papadopoulou, Luca J. Stier, Susanne von Gronau, Brenda A. Schulman

**Affiliations:** 1 Department of Molecular Machines and Signaling, Max Planck Institute of Biochemistry, Martinsried, Germany; 2 Department of Chemistry, School of Natural Sciences, Technical University of Munich, Garching, Germany

**Keywords:** Cryoelectron microscopy, Ubiquitylated proteins

## Abstract

The GID/CTLH family of E3 ubiquitin ligases employs several substrate receptor subunits that recruit distinct degrons, but the substrate recognition mechanisms of the CTLH^MKLN1^ assembly have largely remained elusive. Here, we reconstitute CTLH^MKLN1^-dependent ubiquitylation of three biochemically distinct substrates – MKLN1 itself, ZMYND19, and FAM72A-recruited UNG2, and determine cryo-EM structures of each substrate bound to MKLN1. The structures reveal how MKLN1’s discoidin and Kelch β-propeller domains engage substrates through multivalent contacts. MKLN1 recruits itself through discoidin-Kelch interactions between MKLN1 dimers, forming an assembly that competes with other substrates. ZMYND19 and FAM72A each bind to MKLN1 through loops engaging the discoidin trench domain while their C-terminal Arg residues engage the MKLN1 Kelch central channel, identifying MKLN1 as an Arg/C-degron receptor. The acetylated N-terminus of UNG2 is positioned within a Y-shaped tunnel of FAM72A, defining an adaptor-mediated Ac/N-degron recognition mechanism. Thus, our data reveal how combinatorial deployment of MKLN1 discoidin and Kelch domains enable a single receptor subunit to recognize a diverse substrate repertoire, adding to a complex picture of degron recognition across the GID/CTLH family.

## Abstract (web summary)

The CTLH^MKLN1^ E3 ligase recruits diverse substrates but the underlying mechanisms were unclear. Here, the authors use cryo-EM, biochemistry, and cellular experiments to show how MKLN1 discoidin and Kelch domains combinatorially recognize internal, Arg/C-, and Ac/N-degrons across distinct substrates.

## Introduction

Specificity in the ubiquitin system depends on E3 ligases selecting substrates and positioning them for targeting by the E3–E2~ubiquitin catalytic assembly. The prevailing view is that specificity is achieved by an E3’s dedicated substrate-binding site docking with a cognate degron conserved across its substrates^1,2^. Notably, a common mode of protein targeting by the ubiquitin system involves E3 ligase recognition of N- or C-terminal sequences, termed N- or C-degrons, respectively^3–14^. For homodimeric E3s or E3 complexes, a pair of substrate-binding domains can engage a pair of homologous degron motifs from a single ubiquitylation target, an oligomer, or two individual substrate molecules^15–21^. Although this mechanistic framework has been useful, it increasingly appears incomplete. An emerging concept from biochemical studies is that some E3s harbor multiple distinct domains or regions that combinatorially allow various modes of engagement to enable ubiquitylation of diverse substrates^22–25^. Understanding how such E3 ligase complexes recognize and orient diverse substrates for ubiquitin targeting remains an important problem in ubiquitin biology.

A particularly sophisticated mode for substrate engagement is utilized by the family of E3 ligases in the GID/CTLH family (yeast/higher eukaryotic nomenclature). GID/CTLH E3s are modular, multiprotein complexes built around a core assembly^26–32^. The GID/CTLH core contains a heterodimeric RING-like catalytic module and a multiprotein scaffold^26,30,33,34^. Initial studies in yeast showed that exchangeable, metabolically-induced substrate receptor subunits bind the core GID complex (via an adapter protein) and recruit various N-degron sequences in metabolic enzymes^5,29,30,35–41^. An additional subunit, Gid7, forms a dimer that in turn promotes dimerization of the core assembly into a giant oval “Chelator” E3 complex^15^. In this giant oval configuration, the E3 complex contains two opposing copies of the GID E3 elements: two catalytic modules, two scaffolds, two Gid7 dimer-mediated assembly interfaces, and two substrate receptors bound to adapters. Structural studies revealed that this oval architecture enables substrate engagement in a manner analogous to an organometallic chelator binding a ligand through multiple points of contact. The two opposing Gid4 substrate receptor subunits simultaneously capture Pro/N-degrons from two protomers of the tetrameric substrate, Fbp1. This arrangement drives ubiquitylation of specific Fbp1 lysines that are adjacent to metabolite-binding sites^15^. It was initially thought that the homologous mammalian CTLH E3s would function similarly^31,42,43^ by relying on the GID4 substrate receptor to recruit N-degrons^44–46^. However, subsequent work demonstrated that some CTLH complexes lack the GID4 subunit and instead rely on higher eukaryotic Gid7 paralogs to recruit substrates^42,47,48^. Indeed, the human CTLH subunit WDR26, which is homologous to yeast Gid7 and likewise forms a dimer that mediates formation of a giant oval E3 complex, was proposed to be a GID4-independent substrate receptor^15,48,49^. Unexpectedly, considering that all known yeast GID E3s recruit N-degrons, WDR26’s WD40 domain was found to recognize internal basic degron motifs in the metabolic enzyme substrate NMNAT1^48^. The pair of propellers from adjacent WDR26 subunits can also bind YPEL5, which inhibits substrates from binding to WDR26. YPEL5 has a CRBN-like Yippee domain that can also mediate targeted protein degradation^50^. Thus, mammalian CTLH complexes were found to deploy at least  GID4 and WDR26 for substrate recruitment, with the pair of WDR26 subunits also binding a single Yippee domain protein at their substrate-binding sites^45,46,48–52^.

A different higher eukaryotic subunit, Muskelin (MKLN1), also occupies a Gid7-like architectural position within an oval CTLH E3 assembly^53^. MKLN1 was proposed as yet another substrate-binding receptor^47,54^. However, MKLN1 markedly differs in domain composition, oligomerization properties, and functional output compared to yeast Gid7 and human WDR26. MKLN1 lacks WD40 repeats, and instead displays discoidin and Kelch domains^15,55–58^, suggesting it may employ entirely distinct mechanisms of substrate engagement. Consistent with this notion, MKLN1-dependent CTLH complexes have been implicated in a wide range of biological processes beyond the turnover of metabolic enzymes. Deletion of MKLN1 in mice leads to deficiencies ranging from behavioral changes and synaptic defects to impaired somatic hypermutation and class switch recombination^53,59,60^. MKLN1 mutations in dogs were found to be the cause of lethal acrodermatitis, a disease characterized by severe skin lesions, immunodeficiency, and growth failure^61^. In cellular systems, MKLN1 depletion perturbs cytoskeletal organization and adhesion^62–64^, while studies in flies suggested roles in developmental transitions^47,54^. This breadth of phenotypes implies that MKLN1-dependent CTLH complexes act on a diverse set of substrates. Yet, despite reports of many proteins becoming stabilized upon mutation of MKLN1, and high-resolution cryo-EM data for MKLN1 itself, structural details of CTLH substrate recruitment dependent on MKLN1 have not been experimentally elucidated.

One reason for this gap in our understanding is that MKLN1-dependent ubiquitylation has only been reconstituted with entirely purified reagents for a single substrate, the DNA repair enzyme UNG2, which is recruited to the FAM72A adapter to regulate antibody diversification^53,65^. A cryo-EM map of human CTLH^MKLN1^-FAM72A-UNG2 showed MKLN1 can organize a large oval CTLH E3 assembly similar to that observed with yeast Gid7 and human WDR26^15,48,53^. This map, however, did not reveal the molecular details of substrate recognition. Although two preprints posted on bioRxiv during preparation of our manuscript suggested MKLN1 recognizes Arg/C-degrons, those studies lack experimental structural data altogether, and do not explain how such substrate recruitment places targets relative to the CTLH E3 assembly or the ubiquitylation active sites^66,67^. Furthermore, the existing studies do not explain how MKLN1 itself is subject to ubiquitylation^46,68,69^. As a result, our understanding of the molecular principles that govern substrate recruitment by MKLN1-containing E3 ligases remains limited.

Here, we obtain cryo-EM data for stable proxies of ubiquitylation intermediates—in the form of substrate-bound CTLH E3 complexes with an E2~ubiquitin conjugate—to define structural mechanisms and relative roles of MKLN1 domains in regulating distinct substrates. These maps and focus-refined maps reveal molecular details of subcomplexes, together with biochemical and cell-based data herein and from previous and recent studies^53,66,67^. Our results uncover a diversity of mechanisms for CTLH^MKLN1^ placement of various substrates for ubiquitylation.

## Results

### Biochemical reconstitution of diverse MKLN1-dependent substrates

**Fig. 1:**
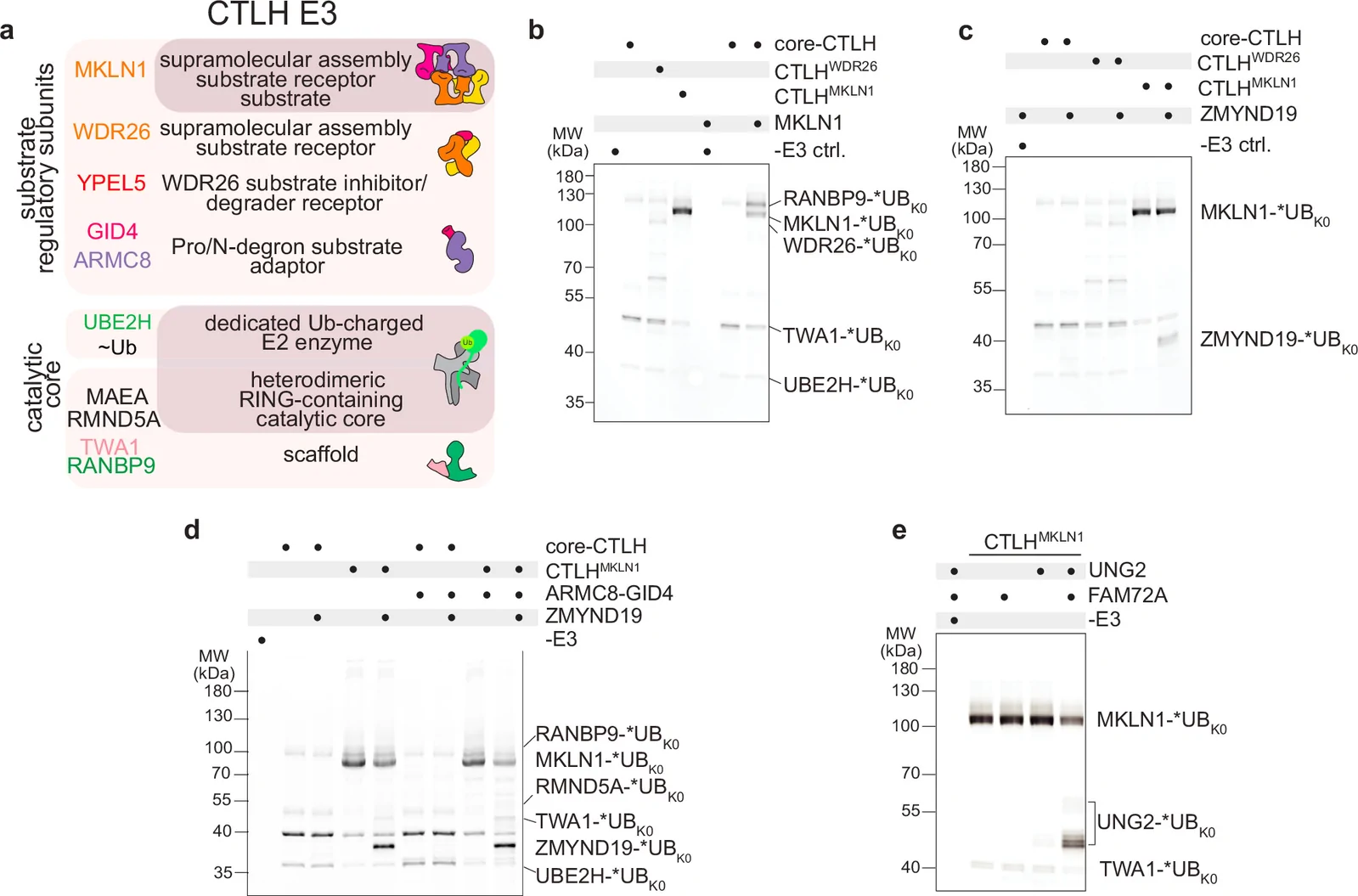
Biochemical reconstitution of CTLH^MKLN1^-dependent substrate ubiquitylation. **a** Schematic of the CTLH E3 complex subunits organized by functional roles. **b** Assays testing MKLN1 ubiquitylation by the indicated CTLH E3 complexes. Ubiquitylation assays used N-terminally fluorescently labeled K0-ubiquitin (*UB_K0_) with all lysines mutated to arginines to visualize direct substrate modification without chain formation, with UBE2H as the E2. **c** In vitro ubiquitylation assays testing ZMYND19 ubiquitylation by the indicated CTLH E3 complexes. **d** Assays testing the effect of the GID4 substrate receptor on CTLH^MKLN1^-dependent ZMYND19 ubiquitylation. **e** Assays testing UNG2 ubiquitylation by CTLH^MKLN1^ in the presence or absence of substrate adapter FAM72A. **b**–**e** All assays were performed for 15 min (*n* ≥ 2 independent replicates). Source data are provided as a Source data file.

As a first step towards defining molecular principles underlying substrate recognition by CTLH^MKLN1^, we used purified reagents to biochemically reconstitute MKLN1-dependent ubiquitylation activity toward previously defined substrates with distinct properties. Of all the subunits of the CTLH complex (Fig. 1a), MKLN1 is the one that is most frequently reported as stabilized upon elimination of CTLH E3 catalytic activity^46,68–71^. ZMYND19 was selected because the E3 subunit directly mediating its recognition is subject to debate^49,68,70^. Meanwhile, UNG2 degradation was shown to depend on the MKLN1-binding adapter protein FAM72A^53,60^.

Experiments compared activity with a “core” CTLH complex (containing RANBP9, TWA1, MAEA, and RMND5A) in the absence or presence of either human Gid7-like subunit WDR26 or MKLN1. We used N-terminally fluorescently-labeled “K0” ubiquitin with all lysines mutated to arginines to visualize direct substrate modification and circumvent complications in interpretation from additional banding from chain formation. Ubiquitylation was achieved by the CTLH E3’s cognate E2, UBE2H^31,72^. MKLN1 was robustly ubiquitylated in our assays, regardless of whether it was co-expressed with the core CTLH E3 complex or added separately (Fig. 1b). Notably, this differs from the other Gid7 ortholog, WDR26, which showed little ubiquitylation. Our assays showed ZMYND19 was ubiquitylated by CTLH^MKLN1^, but not by core-CTLH or CTLH^WDR26^ complexes. ZMYND19 ubiquitylation was also unaffected by the GID4 substrate receptor (Fig. 1c, d, Supplementary Fig. 1). Thus, our biochemical data differ from a previous claim that GID4 recruits ZMYND19, but are consistent with prior cellular studies suggesting that MKLN1 is both a ubiquitylation substrate and a substrate receptor for ZMYND19^49,68^. Meanwhile, our assays recapitulate prior work showing that UNG2 ubiquitylation required CTLH^MKLN1^ and the adapter protein FAM72A^53^ (Fig. 1e).

### Substrate recognition by CTLH^MKLN1^

**Fig. 2:**
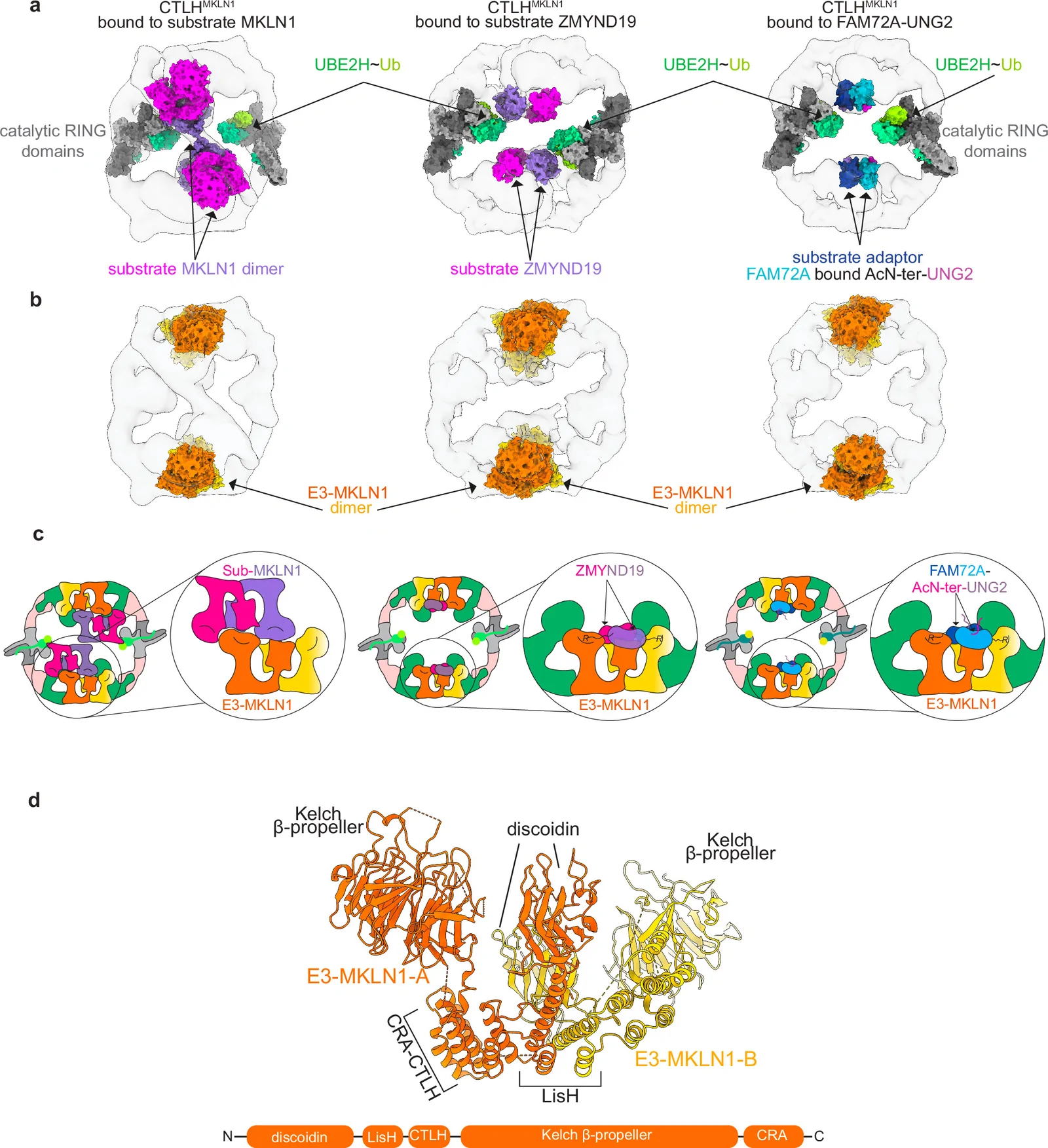
MKLN1 recruits diverse substrates to the center of an oval CTLH assembly. **a** Low-resolution cryo-EM maps of CTLH^MKLN1^-UBE2H~ubiquitin conjugate bound to substrate MKLN1 (left), substrate ZMYND19 (middle), and FAM72A-UNG2 (right), with fitted models from this study and the catalytic module (PDB:8PJN) displayed as surface representations. The UBE2H~ubiquitin conjugate used here is a stable mimic of the ubiquitylation intermediate, wherein ubiquitin’s C-terminus is isopeptide-bonded to a Lys replacement for the UBE2H catalytic Cys. Substrates and substrate adapter are indicated. **b** Cryo-EM maps with fitted model of E3-MKLN1 dimer as surface representation. The model was derived from the map of CTLH^MKLN1^. Briefly, one protomer, E3-MKLN1-A (dark orange), was fit from high-resolution structures of its complex with Sub-MKLN1 (PDB:29UE). The other protomer (E3-MKLN1-B, yellow) was modeled by fitting the domains (discoidin, LisH-CTLH-CRA, Kelch) from the E3-MKLN1-A structure individually into the map. **c** Schematic of the E3-MKLN1 binding to substrates Sub-MKLN1, ZMYND19, and UNG2 (recruited via FAM72A) displayed from left to right. **d** Ribbon diagram of the E3-MKLN1 dimer model generated as described in (**b**). Linear representation of the MKLN1 domains is shown below.

To determine the structural basis for substrate recognition by the CTLH^MKLN1^ E3, we obtained cryo-EM data for complexes representing ubiquitylation of the three substrates, MKLN1, ZMYND19, and FAM72A-UNG2. Ultimately, we visualized only a short peptide sequence from UNG2 bound to MKLN1-FAM72A, suggesting that the remainder of this substrate is flexibly tethered to the E3. The substrate-bound E3 complexes included a stable mimic of the UBE2H~ubiquitin conjugate. Ubiquitin transfer was prevented by use of a non-reactive complex where ubiquitin’s C-terminus was isopeptide-bonded to a Lys replacement for the UBE2H catalytic Cys^72^. Low-resolution cryo-EM maps showed all three substrates in the center of the oval CTLH^MKLN1^ E3, proximal to the UBE2H active sites (Fig. 2a). Details of substrate interactions with the MKLN1 discoidin and Kelch domains, described below, were obtained by fitting the map with structures, either achieved by focused refinements of the complete complex, or from acquiring data for subcomplexes (see “Methods,” Supplementary Table 1, Supplementary Figs. 8–14). In each case, the side-chains described in interactions below were resolved within a single local refinement, ensuring that the relative positioning of interacting residues was directly supported by the cryo-EM density rather than inferred from merging separately refined regions.

The CTLH^MKLN1^ E3s resemble Chelator-GID and CTLH^WDR26^ E3 complexes^15,48^, in that a pair of Gid7 orthologs (here MKLN1) homodimerize to close the oval-shaped E3 ligase assembly (Fig. 2b, c, Supplementary Fig. 2a). Gid7 and WDR26 form oval E3s through their LisH, CTLH, and WD40 beta-propeller domains, where the LisH domain mediates homodimerization, and the CTLH domain (together with other domains) mediates interactions with core E3 subunits^30,57,58,73^. MKLN1 is characterized by LisH, CTLH, discoidin, and Kelch β-propeller domains (Fig. 2d). The former two domains function analogously to those in Gid7 and WDR26, while the latter two domains mediate substrate-binding in various ways described below.

### E3-MKLN1 recognition of substrate MKLN1

**Fig. 3:**
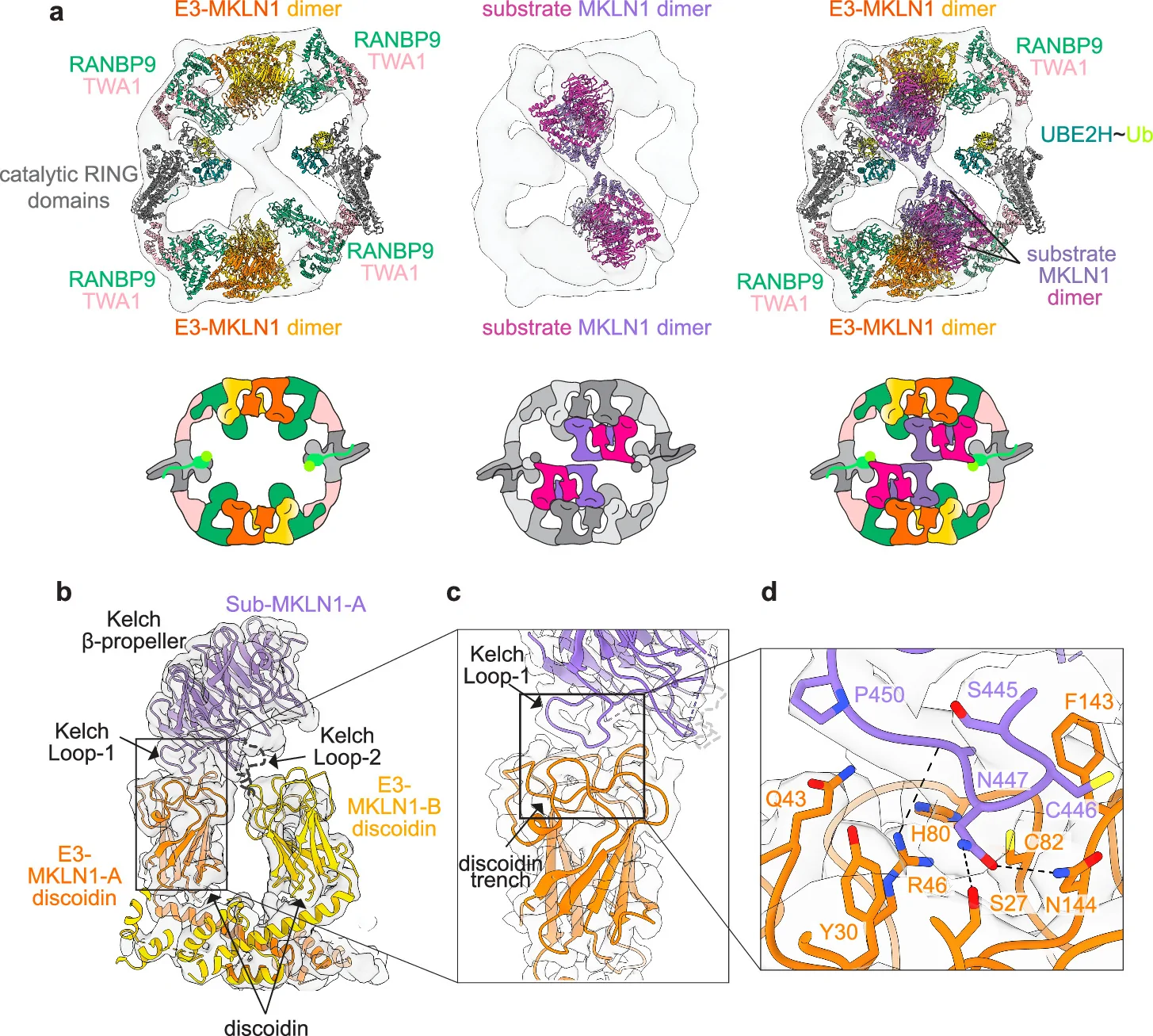
CTLH^MKLN1^-UBE2H~ubiquitin recruits a dimeric MKLN1 substrate through discoidin–Kelch domain contacts. **a** Cryo-EM map of complex containing CTLH^MKLN1^-UBE2H~ubiquitin bound to substrate MKLN1, shown with fitted structures and schematic diagrams below, illustrating subunits within the assembly. Left: Cryo-EM map fitted with the scaffolding module RANBP9–TWA1 (this study, PDB: 29UG), E3-MKLN1 dimer, modeled from the E3-MKLN1-A–Sub-MKLN1-A high-resolution structure (this study, PDB: 29UE), and catalytic module extracted from PDB: 8PJN. Middle: the same cryo-EM map with the Sub-MKLN1 dimer modeled as described for the E3-MKLN1 dimer. Right: all fitted structures shown together, depicting the complete CTLH^MKLN1^–Sub-MKLN1 assembly. **b** Map of E3-MKLN1–Sub-MKLN1 tetrameric assembly shown over model of E3-MKLN1 discoidin domains engaging the Sub-MKLN1-A Kelch β-propeller fitted from E3-MKLN1-A–Sub-MKLN1-A high-resolution structure (PDB: 29UE). **c** Close-up view of the boxed region in (**b**) showing the E3-MKLN1-A discoidin trench surface and Kelch Loop-1 from Sub-MKLN1-A engaging the trench, from 3.7 Å resolution structure of E3-MKLN-A–Sub-MKLN1-A subcomplex (PDB: 29UE). **d** Molecular details of the E3-MKLN1-A discoidin trench–Sub-MKLN1-A Kelch Loop-1 interface, highlighting key discoidin residues contacting Sub-MKLN1-A Asn447 and surrounding loop residues, with hydrogen bonds depicted as dashed lines.

The complex with MKLN1 differed strikingly from prior structures of Chelator-GID or CTLH^WDR26^ E3 complexes, which feature a hollow cavity for binding to substrates in the center of their oval shape (Supplementary Fig. 2b). Here, however, the oval was filled by two additional MKLN1 protomers (Fig. 3a). The structural data suggest how MKLN1 recruits itself as a substrate: while one MKLN1 dimer (hereafter “E3-MKLN1”) integrates into the CTLH oval assembly, the second MKLN1 dimer (hereafter “Sub-MKLN1”) is positioned as a substrate (Supplementary Fig. 3). We refer to the two protomers in each MKLN1 dimer as A and B. The E3-substrate interactions were visualized by determining the structure of an E3-MKLN1-A complex with Sub-MKLN1-A from a 3.7 Å resolution focus-refined map, and then fitting domains from this structure into the E3-MKLN1-B and Sub-MKLN1-B densities in a 4.0 Å resolution focused-refined map showing the MKLN1 tetramer.

The E3-MKLN1 and substrate MKLN1 dimers are related by approximate 2-fold symmetry, such that the E3-MKLN1-A subunit is equivalent to the Sub-MKLN1-A subunit, and the E3-MKLN1-B subunit is equivalent to the Sub-MKLN1-B subunit (Supplementary Fig. 3b). Within each dimer, however, the two protomers are asymmetric. Such arrangements had been observed in prior cryo-EM maps of MKLN1 alone (EMD-45088, EMD-41612)^53^ and in subcomplexes (EMD-12547)^15^. This arrangement had not been previously examined for the role of the tetrameric assembly in defining MKLN1 as a CTLH E3 substrate.

The MKLN1 substrate is recruited to the CTLH^MKLN1^ E3 through multiple interactions. The discoidin domains from both E3 protomers simultaneously engage the Kelch propeller domain of Sub-MKLN1-A (Fig. 3b), while the Kelch domain of E3-MKLN1-A reciprocally contacts the discoidin domains of both substrate protomers (Supplementary Fig. 3c). The discoidin domain comprises an 8-stranded β-barrel with a helical turn between strands 1 and 2 (Fig. 3c). Extended loops connecting the strands form a trench-like surface at the Kelch-facing end of the barrel. The E3-MKLN1-A discoidin domain trench embraces a five-residue loop (hereafter referred to as Loop-1) between blades 4 and 5 from the Sub-MKLN1-A Kelch domain. The Loop-1 residue Asn447 lies at the center of this interface. It embeds in the discoidin trench of E3-MKLN1-A, where it is surrounded by Tyr30, Arg46, Cys82, Asn144, and Ser27, and forms hydrogen bonds with the latter two side chains (Fig. 3d). The remainder of the substrate loop is secured by backbone and van der Waals contacts with Gln43, His80, and Phe143 from the E3-MKLN1-A discoidin domain.

The E3-MKLN1-A discoidin domain interaction with the Sub-MKLN1-B Kelch domain is visible only at lower resolution. Nonetheless, the data show this involves a distinct discoidin domain surface contacting a “Loop-2” between the central two strands in blade 4 of the Kelch domain (Supplementary Fig. 3c).

The Kelch domains of E3-MKLN1-B and Sub-MKLN1-B are located at the distal ends of the tetrameric assembly and are not well-resolved in our higher-resolution maps. Their positions suggest potential for interactions between E3-MKLN1-B and Sub-MKLN1-A, and between E3-MKLN1-A and Sub-MKLN1-B Kelch domains (Supplementary Fig. 3d). However, we did not obtain a map at sufficiently high resolution to allow definitive residue assignments for such interactions.

### E3-MKLN1 recognition of substrate ZMYND19

**Fig. 4:**
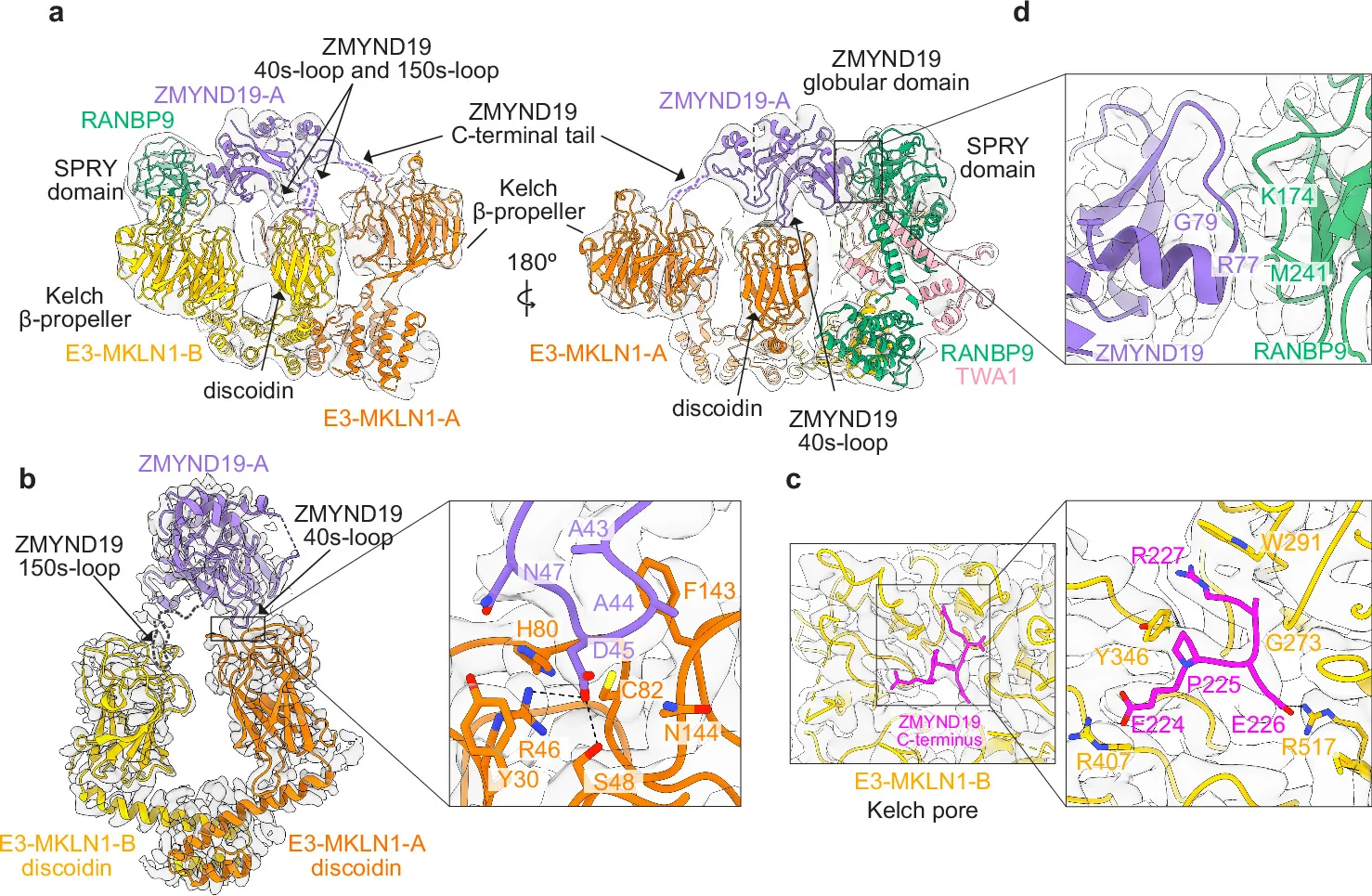
ZMYND19 is recruited to CTLH^MKLN1^ through multivalent contacts with the discoidin domain and Kelch β-propeller channel. **a** Two views of cryo-EM map of MKLN1–RANBP9–TWA1 subcomplex bound to ZMYND19-A, with fitted model from this study (PDB: 29UG). Views illustrate ZMYND19-A 40s-loop and 150s-loop contacts with the E3-MKLN1-A and E3-MKLN1-B discoidin domains, the ZMYND19 C-terminal tail extending toward the E3-MKLN1-B Kelch β-propeller, and the ZMYND19-A globular domain contacts with the RANBP9 SPRY domain. Subunits and domains are indicated. **b** Focus-refined cryo-EM map resolved to 3.3 Å, with fitted model, showing ZMYND19 40s-loop engaging the E3-MKLN1-A discoidin trench. Close-up highlights key discoidin residues contacting ZMYND19 Asp45 and surrounding loop residues, with hydrogen bonds indicated by dashed lines. **c** Focus-refined cryo-EM map resolved to 3.35 Å, showing the ZMYND19-B C-terminal residues fitted into the E3-MKLN1-B Kelch domain channel. Close-up highlights the Arg/C-degron recognition interface, with key side chains shown in sticks and hydrogen bonds indicated by dashed lines. **d** Focus-refined cryo-EM map resolved to 3.25 Å, showing backbone-mediated contacts between ZMYND19-A and the RANBP9 SPRY domain. Maps shown in (**b**–**d**) were sharpened with DeepEMhancer.

The maps of the CTLH^MKLN1^-UBE2H~ubiquitin complex with ZMYND19 revealed three striking features (Fig. 2a, middle). First, the only MKLN1 dimer in this map was the E3-MKLN1, i.e., within the oval CTLH E3 assembly. Second, the E3-MKLN1 dimer connects to additional density corresponding to two molecules of ZMYND19 in an asymmetric arrangement. Third, different 3D classes showed varying extents of density for ZMYND19. The entirety of one molecule (hereafter termed ZMYND19-A) is visible in all classes, along with the C-terminus of the second (hereafter termed ZMYND19-B) (Fig. 4a). A subset of classes also show density across the full ZMYND19-B protomer, albeit at low contour and at relatively low resolution (Supplementary Fig. 4a).

Maps from a MKLN1–RANBP9–TWA1 subcomplex showed detailed interactions with ZMYND19 (Fig. 4a, Supplementary Fig. 4b). For ZMYND19-A—i.e., the better-resolved molecule—one loop (hereafter termed 40s-loop) from the globular ZMYND domain binds the E3-MKLN1 protomer A discoidin domain trench. Another ZMYND19 loop (hereafter termed 150s-loop) extends towards the discoidin domain trench from the E3-MKLN1-B subunit. Meanwhile, the ZMYND19 C-terminal tails from both protomers extend towards the E3-MKLN1 Kelch domains.

The contacts between the E3-MKLN1-A-discoidin domain and the ZMYND19 40s-loop were resolved in detail after focus refinement (Fig. 4b). Interactions center around ZMYND19 Asp45, which is surrounded by Tyr30, Arg46, Ser48, Cys82, and Asn144 from the discoidin domain trench. While these interactions resemble those of Sub-MKLN1-A Asn447, the ZMYND19 Asp45 side-chain forms electrostatic interactions with E3-MKLN1-A Arg46 and Ser48 (Fig. 4b). Although limited resolution precludes unambiguous assignment of contacts with the ZMYND19 150s-loop, we note that this loop sequence (Asn153-Gly154-Asp155) is palindromic to that of the 40s-loop (Asp45-Gly46-Asn47), raising the possibility of analogous interactions in the reverse orientation.

A focus-refined map visualized the last four ZMYND19-B residues (Glu224-Pro225-Glu226-Arg227) fitting inside the E3-MKLN1-B Kelch domain central channel (Fig. 4c). The C-terminal ZMYND19 Arg227 is sandwiched between Gly273 and Trp291 from E3-MKLN1, with which it forms π-stacking interactions. Adjacent contacts further support this Arg/C-terminal motif. ZMYND19 Glu226 hydrogen bonds with MKLN1 Arg517. ZMYND19 Pro225 packs against the aromatic ring of MKLN1 Tyr346, and ZMYND19 Glu224 is positioned near MKLN1 Arg407.

Another ZMYND domain surface of molecule A, centered around Gly78 and Gly79, approaches the SPRY domain from the RANBP9 subunit adjacent to E3-MKLN1-B (Fig. 4d). These contacts are backbone-mediated, suggesting a structural rather than side-chain specific contribution to ZMYND19 recognition.

### E3-MKLN1 recognition of substrate UNG2 via FAM72A adapter

**Fig. 5:**
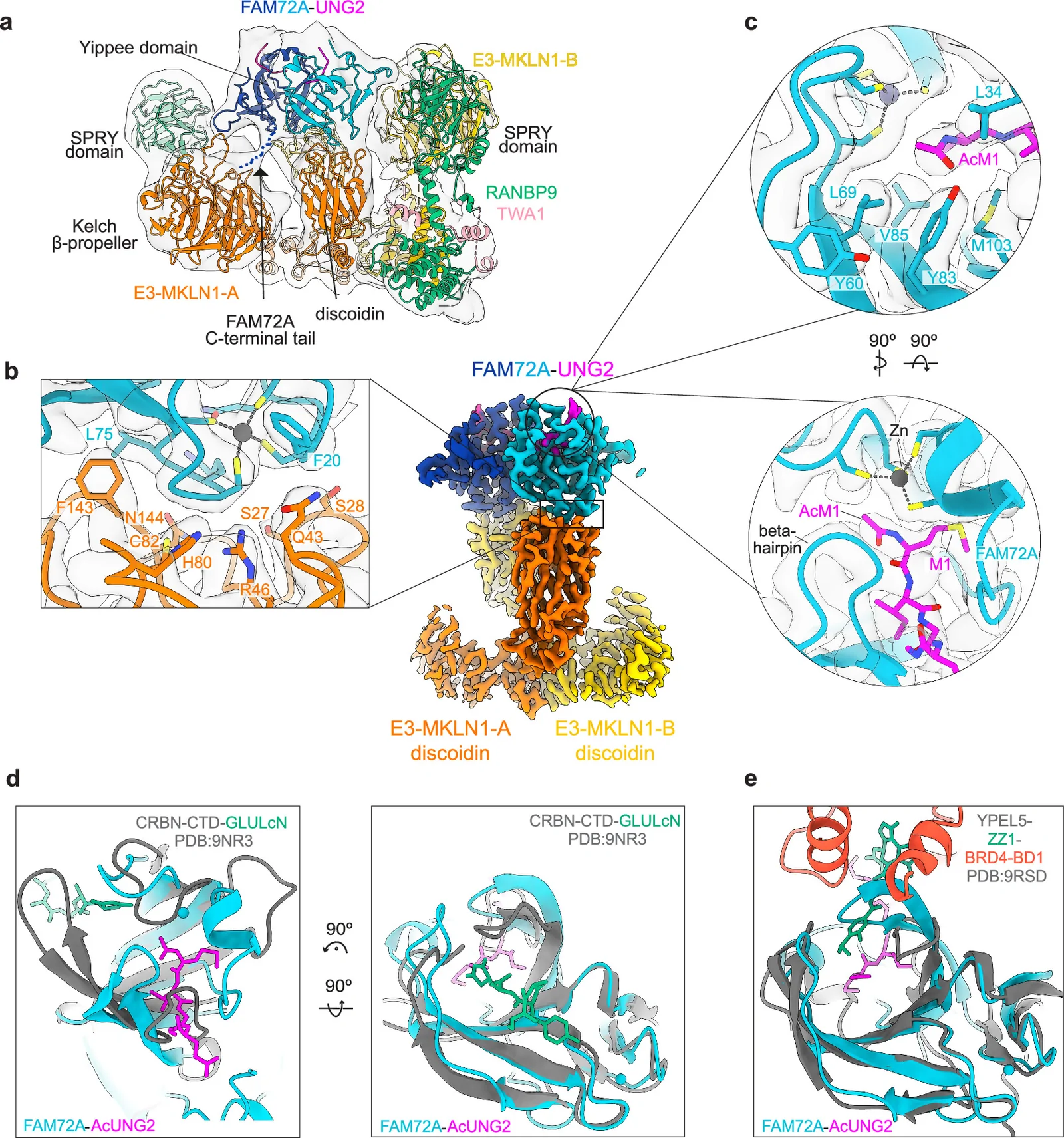
Structure of MKLN1-FAM72A-UNG2 reveals FAM72A Yippee domain engagement of the discoidin trench and FAM72A-mediated recognition of the acetylated UNG2 N-terminus. **a** Cryo-EM map of the MKLN1–RANBP9–TWA1 subcomplex bound to FAM72A-UNG2, fit with structures from this study. The composite model shows each FAM72A engaging the MKLN1 discoidin domain, and the FAM72A C-terminal tail extending toward the opposite MKLN1 Kelch β-propeller. **b** Left: close-up of the FAM72A zinc-coordinated Yippee fold–MKLN1 discoidin trench interface, with key interacting residues labeled. Middle: 3.0 Å resolution DeepEMhancer-sharpened cryo-EM map of the MKLN1 discoidin-FAM72A-UNG2. **c** Close-up of the UNG2 acetylated N-terminus (AcM1, magenta) inserted into the Y-junction tunnel inside the FAM72A Yippee domain. **d** Structural comparison of FAM72A–AcUNG2 (cyan) with CRBN C-terminal domain (CTD) bound to peptide from substrate GLUL modified with C-terminal Asn cyclic imide (cN) (PDB: 9NR3), illustrating both complexes recognizing protein termini at the center of the groove despite opposite substrate orientations. **e** Structural comparison of FAM72A–AcUNG2 with YPEL5–ZZ1–BRD4_BD1_ (PDB: 9RSD), showing similar substrate positioning relative to the Yippee domain groove. The FAM72A-specific zinc-binding element and extended β-hairpin enclosing the UNG2 acetylated N-terminus are indicated.

Previous studies indicated a distinct substrate-binding mode for UNG2: CTLH^MKLN1^ binds FAM72A, which in turn recruits UNG2^53,60,74^. Although published cryo-EM maps for the CTLH^MKLN1^-FAM72A-UNG2 complex^53^ are consistent with this model, they do not reveal molecular details. We obtained further structural insights into this E3-substrate assembly from cryo-EM data for a complex poised for ubiquitylation and for a subcomplex. In agreement with prior studies, the FAM72A structure consists of two domains—a globular region with a Yippee-domain fold stabilized by two zinc atoms, and a C-terminal tail terminating with an Arg residue. Also, as described^53^, two FAM72A molecules are symmetrically positioned in the center of the oval-shaped CTLH^MKLN1^ complex. The Yippee domain of one FAM72A molecule binds the E3-MKLN1-B protomer discoidin domain, while the FAM72A C-terminus extends toward and inserts into the E3-MKLN1-A Kelch domain central channel (Fig. 5a). The second FAM72A molecule makes the equivalent interactions with the opposite MKLN1 protomers.

Our 3.0 Å resolution map resolved the detailed interactions between the MKLN1 discoidin domain and FAM72A Yippee domain, and the basis for UNG2 recruitment to FAM72A (Fig. 5b). A convex zinc-coordinated portion of the FAM72A Yippee fold complements the concave surface of the MKLN1 discoidin trench. A FAM72A loop—adjacent to the zinc-binding site—embeds in the trench.

The structure also shows UNG2’s acetylated N-terminal region inserted into tunnels arranged like a Y-junction inside FAM72A (Fig. 5c). UNG2 residues 2–5 insert through a long tunnel. The intersecting tunnels are punctuated by the second zinc-binding site, and accommodate the acetylated N-terminal Met1. The acetyl and Met1 side chain are splayed apart and directed towards opposite sides of the Y-junction. Thus, our cryo-EM data now provide a structural understanding for many previous biochemical findings indicating that the N-terminal region of UNG2 is crucial for binding FAM72A^53,75,76^.

FAM72A’s substrate-binding domain shares a common fold with CRBN and YPEL5. Comparing their modes of substrate recruitment shows their distinguishing features. Superimposing FAM72A and CRBN shows that they engage targets in inverse directions, at opposite ends of their substrate-binding grooves^77^. Yet, they both recognize protein termini—an acetylated N-terminus or C-terminal cyclic imide—in the middle of the groove (Fig. 5d). FAM72A and YPEL5 (together with a degrader molecule) recruit their targets to the same side of the groove, but YPEL5 lacks the FAM72A-specific zinc-binding element that binds UNG2’s acetylated Met1 (Fig. 5e)^48,50^. FAM72A is closed around its substrate: an extended FAM72A beta-hairpin wraps around UNG2 to approach the second zinc-binding region in FAM72A.

### Effects of MKLN1 Kelch domain mutations

**Fig. 6:**
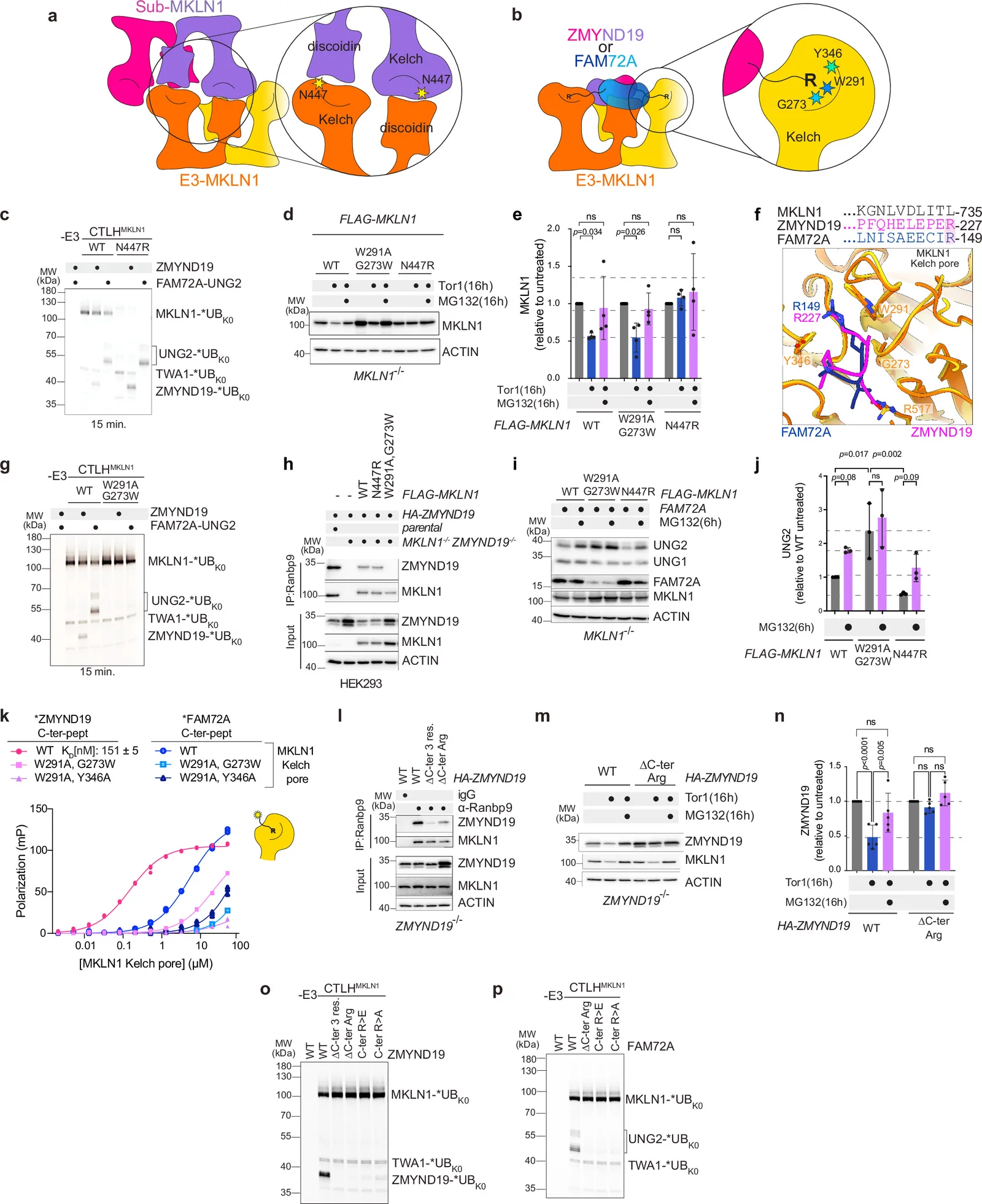
Structure-guided mutational analysis of the MKLN1 Kelch domain in CTLH^MKLN1^ substrate recognition. **a** Cartoon depicting the E3-MKLN1–Sub-MKLN1 tetrameric assembly, highlighting the discoidin and Kelch domains, and Asn447 within Kelch domain Loop-1 (starred), which is central to the E3-MKLN1–Sub-MKLN1 interface. **b** Cartoon depicting the Kelch domain central channel, which recognizes the C-terminal Arg of ZMYND19 and FAM72A, highlighting contact residues Gly273, Trp291, and Tyr346 (starred). **c** In vitro ubiquitylation assay comparing CTLH^MKLN1^ WT and the N447R mutant toward MKLN1, ZMYND19, and FAM72A-UNG2, *n* = 3 technical replicates. **d** Immunoblot analysis of MKLN1 levels in MKLN1-deficient cells transiently expressing WT, W291A/G273W, or N447R *3×Flag-MKLN1*, monitoring Torin1-dependent degradation after 16 h, Torin1 + MG132 (16 h) co-treatment, or matched DMSO controls. Actin serves as a loading control. **e** Quantification of MKLN1 immunoblots from (**d**). Data from each well were normalized to actin, and then each set expressing a particular *3×Flag-MKLN1* construct was normalized to the DMSO control for that construct. The graph shows results of mean ± s.d. of *n* = 4 biological replicates. *P*-values were calculated using two-way ANOVA with Dunnett’s multiple comparison tests (*p*-value significance <0.05). **f** Top: sequence alignment of MKLN1, ZMYND19, and FAM72A C-termini. Bottom: superposition of cryo-EM structures of FAM72A (blue) and ZMYND19 (magenta) C-termini bound within the E3-MKLN1 Kelch domain central channel, highlighting their similar contacts between MKLN1 Gly273, Trp291, Tyr346, Arg407, and Arg517, and the terminal Arg residues of FAM72A (Arg149) and ZMYND19 (Arg227). **g** In vitro ubiquitylation assay examining effect of W291A/G273W Kelch central channel mutant on CTLH^MKLN1^ activity toward MKLN1, ZMYND19 and FAM72A-UNG2, *n* = 3 technical replicates. **h** Co-immunoprecipitation (using anti-RANBP9 antibody) in MKLN1- and ZMYND19-deficient HEK293 cells transiently expressing *HA-ZMYND19* and *3×Flag-MKLN1* (WT or indicated N447R, or W291A/G273W mutants), assessing the role of Kelch channel surfaces on ZMYND19 association with the CTLH complex. Immunoprecipitates and inputs are shown. Actin serves as a loading control (*n* = 2 biological replicates). **i** Immunoblot analysis of UNG and FAM72A levels in *MKLN1*^−/−^ knockout HEK293 cells transiently co-transfected with constructs expressing *FAM72A* and *3×Flag-MKLN1* WT or indicated mutant versions (W291A/G273W, or N447R) following mock (DMSO) treatment or treatment with proteasome inhibitor MG132. **j** Quantification of UNG2 immunoblots from (**i**). Data from each well were normalized to actin, and then to the DMSO control for cells transfected with a construct expressing WT *3×Flag-MKLN1*. The graph shows results of mean ± s.d. of *n* = 3 biological replicates. *P*-values were calculated using two-way ANOVA with Dunnett’s multiple comparison tests (*p*-value significance <0.1). **k** Fluorescence polarization assay measuring binding of fluorescein-labeled peptides corresponding to the C-terminal 19 residues of ZMYND19 or FAM72A to the various versions of the MKLN1 Kelch domain (WT, W291A/G273W, or W291A/Y346A). *K*_D_ for the ZMYND19 peptide–WT MKLN1 interaction is indicated. Data shown are the mean ± s.d. (*n* = 3 technical replicates). **l** Co-immunoprecipitation via anti-RANBP9 antibody or rabbit IgG as a negative control in parental and *ZMYND19*^−/−^ knockout HEK293 cells transfected with constructs expressing *HA-ZMYND19* (WT, ΔC-ter 3 res, or ΔC-ter Arg), assessing the role of the ZMYND19 C-terminal region in association with the CTLH complex. Immunoprecipitates and inputs are shown. Actin serves as a loading control (*n* = 5 biological replicates). **m** Immunoblot analysis of ZMYND19 and MKLN1 levels in *ZMYND19*^*−/−*^ knockout HEK293 cells transiently expressing *HA-ZMYND19* WT or ΔC-ter Arg mutant, and treated either with DMSO as control, Torin1, or Torin1 and proteasome inhibitor MG132. Actin serves as a loading control. **n** Quantification of ZMYND19 immunoblots from (**m**). Data from each well were normalized to actin, and then each set transfected with a particular *HA-ZMYND19* expression construct was normalized to the DMSO control for that construct. The graph shows results of mean ± s.d. of *n* = 5 biological replicates. *P*-values were calculated using two-way ANOVA with Dunnett’s multiple comparison tests (*p*-value significance <0.05). In vitro ubiquitylation assays examining the effect of C-terminal mutations in ZMYND19 (**o**) or FAM72A (**p**) on ubiquitylation by CTLH^MKLN1^. *n* = 2 technical replicates. Source data are provided as a Source data file.

Altogether, the cryo-EM structures showed two distinct regions of the MKLN1 Kelch domain involved in substrate or adapter recruitment (Fig. 6a, b). First, the MKLN1 substrate is recruited via Kelch domain loops. A Loop-1 N447R substitution designed to disrupt interaction in trans with another MKLN1’s discoidin domain converted isolated MKLN1 from a tetramer to a dimer (Supplementary Fig. 5a). This mutation also eliminated MKLN1 ubiquitylation in vitro and increased MKLN1 levels in cells to a degree similar to that observed for WT MKLN1 upon treatment with a proteasome inhibitor (Fig. 6c–e). Similar effects were observed in the in vitro assays upon wholesale replacement of the Loop-1 sequence (Supplementary Fig. 5a, b). Strikingly, the N447R mutation enhanced ubiquitylation of ZMYND19 and UNG2 (Fig. 6c). This effect agrees with our structural data showing that Sub-MKLN1 binding is mutually exclusive with other substrates, and that neither ZMYND19 nor FAM72A engages the Kelch Loop-1 surface. The data indicate that MKLN1 self-recruitment limits CTLH^MKLN1^ E3 access to other substrates competing for the discoidin domain trench.

The second role for the E3-MKLN1 Kelch domain is its central channel binding a substrate’s C-terminal Arg, as observed at high resolution in our structure with ZMYND19. Sequence comparison confirms that ZMYND19 and FAM72A both terminate with an Arg, whereas MKLN1 does not. A 3.7 Å resolution focus-refined cryo-EM map of the MKLN1-FAM72A-UNG2 subcomplex also shows the last three FAM72A residues bound within the E3-MKLN1 Kelch domain central channel, much like the structure with ZMYND19 (Fig. 6f, Supplementary Fig. 5c). Accordingly, a MKLN1 mutant with residue substitutions in the Kelch central channel (W291A which would eliminate aromatic interactions with C-terminal Arg, and G273W that would exclude the C-terminal Arg from accessing the channel) was impaired for mediating in vitro ubiquitylation of ZMYND19 and the FAM72A-dependent substrate UNG2 (Fig. 6g). Upon co-expression in HEK293 cells, the association of ZMYND19 with the CTLH complex was disrupted for the Kelch channel mutant (W291A and G273W mutant) (Fig. 6h). Cell treatment with the proteasome inhibitor MG132 increased the level of UNG2 for WT MKLN1 and the control N447R mutant. However, the effect of proteasome inhibition was not observed for the W291A G273W mutant impaired in binding the C-terminal Arg motif (Fig. 6i, j). Meanwhile, the levels of exogenously expressed FAM72A substantially increased upon co-expression with MKLN1, but not with the W291A and G273W mutants (Fig. 6i). The latter data suggest that MKLN1 may stabilize FAM72A, potentially through promoting its folding, much like levels of the structurally homologous YPEL5 depend on co-expression of its Gid7-like partner WDR26^48^. Notably, mutation of the Kelch channel did not affect MKLN1 ubiquitylation or degradation (Fig. 6d, g), consistent with our structural data showing Sub-MKLN1 does not engage through this region.

Several experiments considering MKLN1 Kelch channel engagement from the perspective of its binding partners showed a key role for their C-terminal Arg. Fluorescein-labeled peptides corresponding to the 19 C-terminal residues of ZMYND19 and FAM72A are sufficient to bind the MKLN1 Kelch propeller. The peptide corresponding to the substrate, ZMYND19, bound with a dissociation constant (*K*_D_) of 151 nM ± 5 nM, whereas that from the adapter FAM72A bound less tightly, in the micromolar range (Fig. 6k). For both peptides, the binding was substantially reduced for the Kelch channel mutants (W291A G273W, as well as W291A Y346A that additionally disrupts contacts with the penultimate residue). Moreover, a full-length WT ZMYND19 effectively displaced the labeled ZMYND19 peptide from the MKLN1 Kelch channel, whereas a ZMYND19 construct lacking the C-terminal Arg failed to compete (Supplementary Fig. 5d). In cellular assays, C-terminal mutants in ZMYND19 were impaired for association with the CTLH complex (Fig. 6l). Deleting the C-terminal Arg increased the cellular level of ZMYND19, and unlike WT ZMYND19 its abundance did not significantly further increase upon cell treatment with the proteasome inhibitor MG132 (Fig. 6m, n). Additionally, ZMYND19 ubiquitylation in vitro was essentially abolished for C-terminal deletion mutants and the C-terminal R227E mutant. Shortening the C-terminal side chain with an R227A substitution partially reduced ubiquitylation with purified proteins (Fig. 6o). Likewise, deleting the FAM72A C-terminal Arg or mutating it to Asp or Ala also impaired UNG2 ubiquitylation (Fig. 6p). Thus, the structural and biochemical data reveal that MKLN1 recognizes a C-terminal Arg. The concept that MKLN1 is an Arg/C-degron-binding E3 was also proposed by work posted on bioRxiv while our manuscript was in preparation^66,67^.

### Effects of MKLN1 discoidin domain mutations

**Fig. 7:**
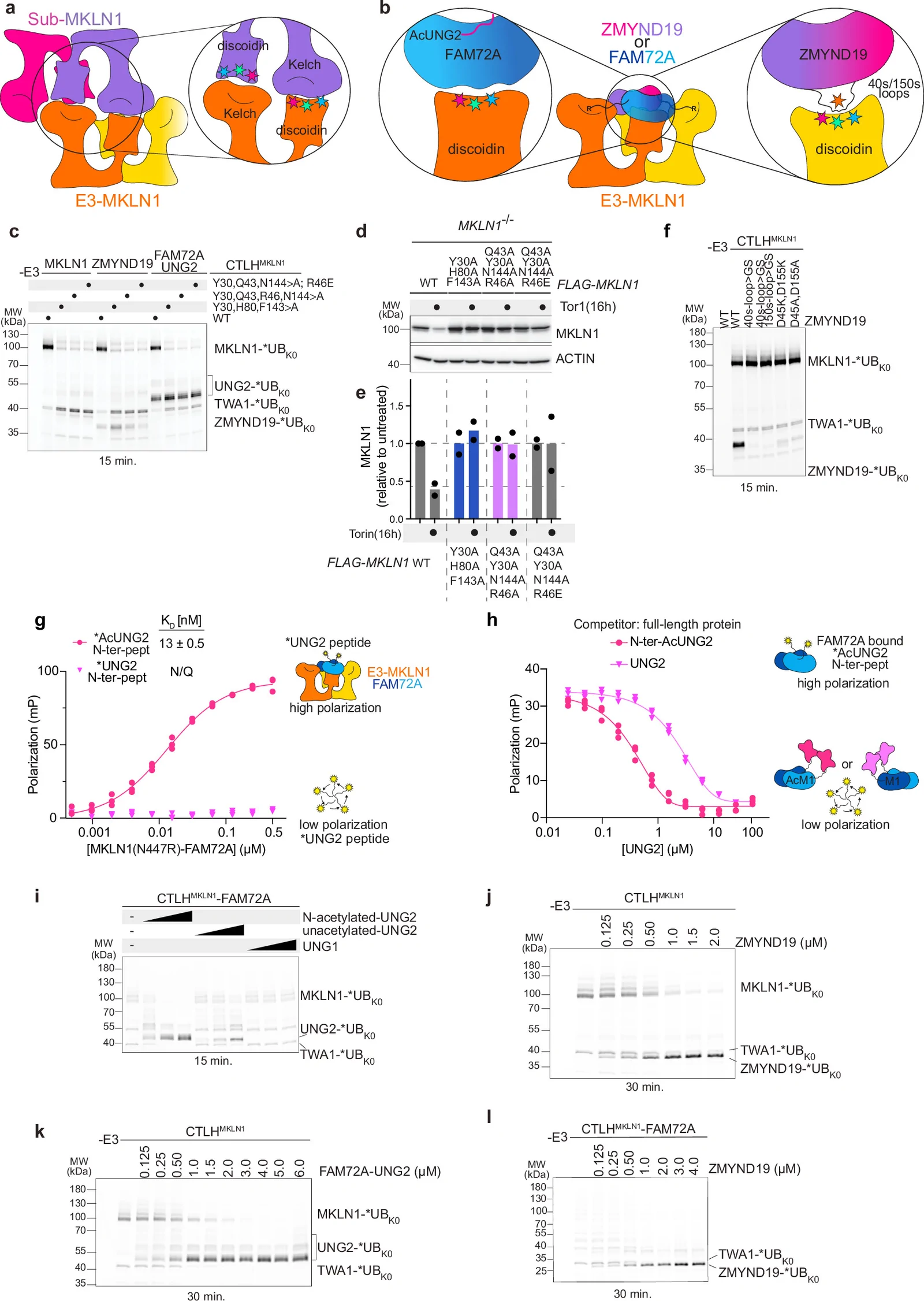
Structure-guided mutational analysis of MKLN1 engagement of internal loops and adapter-mediated binding to acetylated N-terminal motif. **a** Cartoon depicting the Sub-MKLN1 complex, highlighting discoidin domain trench contacts with the opposing Sub-MKLN1 Kelch domain. **b** Cartoon depicting ZMYND19 or FAM72A engagement of the discoidin domain trench. **c** In vitro ubiquitylation assays comparing CTLH^MKLN1^ WT and discoidin trench mutants toward ZMYND19, FAM72A-UNG2, and MKLN1; *n* = 3 technical replicates. **d** Immunoblot analysis measuring MKLN1 levels in *MKLN1*^−/−^ knockout cells transiently expressing WT *3×Flag-MKLN1* or indicated discoidin trench mutants, following 16 h treatment with Torin1 or DMSO as control. Actin serves as a loading control. **e** Quantification of MKLN1 immunoblots from (**d**). Data from each well were normalized to actin, and then each set expressing a particular *3×Flag-MKLN1* construct was normalized to the DMSO control for that construct. The graph shows results of the mean *n* = 2 biological replicates. **f** In vitro ubiquitylation assays examining effect of ZMYND19 trench-inserting loop substitutions (40s-loop>GS; 40s-loop and 150s-loop>GS) and central loop residue substitutions (D45K/D155K; D45A/D155A) on ZMYND19 ubiquitylation by CTLH^MKLN1^ *n* = 3 technical replicates. **g** Fluorescence polarization assay measuring binding of N-terminally acetylated (AcUNG2) or unacetylated UNG2 N-terminal peptides to MKLN1(N447R)-FAM72A. *K*_D_ for the acetylated peptide is indicated (mean ± s.d.; *n* = 3 technical replicates); N/Q, not quantifiable (no detectable binding). **h** Competition fluorescence polarization assay in which full-length N-terminally acetylated or unacetylated UNG2 was titrated against the labeled AcUNG2 N-terminal peptide bound to FAM72A; *n* = 3 technical replicates. **i** In vitro ubiquitylation assay comparing N-terminally acetylated UNG2 (produced in insect cells), unacetylated UNG2 (produced in bacteria), and UNG1 (produced in bacteria), as substrates for CTLH^MKLN1^-FAM72A. **j** In vitro ubiquitylation assay in which increasing concentrations of ZMYND19 were titrated into the CTLH^MKLN1^ ubiquitylation reaction, monitoring competition between Sub-MKLN1 and ZMYND19 ubiquitylation. **k** As in (**j**), but with increasing concentrations of FAM72A-UNG2 titrated into the CTLH^MKLN1^ ubiquitylation reaction. **l** In vitro ubiquitylation assay in which increasing concentrations of ZMYND19 were titrated into the ubiquitylation reaction with a pre-assembled CTLH^MKLN1^-FAM72A (without UNG2) complex, monitoring competition between residual complex autoubiquitylation and ZMYND19 ubiquitylation. **i**, **j** *n* = 2 technical replicates. Source data are provided as a Source data file.

The structural data show that a plethora of side chains from the MKLN1 discoidin domain trench differentially contact various MKLN1 binding partners (Fig. 7a, b). Ala replacements for MKLN1 discoidin domain residues Tyr30, His80, and Phe143—which contact the opposing MKLN1 Kelch domain Loop-1 (Fig. 3d)—reduced MKLN1 ubiquitylation in vitro (Fig. 7c). Importantly, the effects of this discoidin domain mutant paralleled those of the Kelch Loop-1 mutant (see Fig. 6c, Supplementary Fig. 5b), with increased alternative autoubiquitylation of the TWA1 subunit, and slightly increased ubiquitylation of ZMYND19 and UNG2 (Fig. 7c). Meanwhile, eliminating a different constellation of side-chains that also include a key ZMYND19-binding residue (R46E) reduced ubiquitylation of ZMYND19 along with MKLN1. In cells, these mutants block the proteasome-dependent MKLN1 degradation pathway (Fig. 7d, e).

In vitro ubiquitylation of ZMYND19 was also impaired by mutation of the ZMYND domain loops that bind the MKLN1 discoidin domains (40s-loop alone, or together with mutation of the 150s-loop, Fig. 7f). Similar results were also observed for mutation of the central Asp residues from the two loops (Asp45 from the 40s-Loop and Asp155 from the 150s-Loop). However, discoidin domain mutants at the interface with ZMYND19, and these ZMYND19 loop mutants retained interaction when exogenously expressed in cells (Supplementary Fig. 6a, b). Thus, under the conditions of our cellular assays, the ZMYND19 interactions with the MKLN1 Kelch domain, or other regions of the CTLH E3, may be sufficient to mediate interaction. Those mutants, however, lost the ability to support FAM72A levels (Supplementary Fig. 6c, d).

### Role of UNG2 N-terminal acetylation in binding FAM72A

The CTLH^MKLN1^-FAM72A recognition of UNG2’s acetylated N-terminus was striking considering that the first-characterized substrates of GID/CTLH E3s are recognized by N-degrons binding to the GID4 subunit^5,39^. To determine the importance of the acetyl group for FAM72A binding, we tested whether peptides corresponding to the UNG2 N-terminal region could compete with full-length substrate^78^. Addition of the N-terminally acetylated peptide reduced UNG2 ubiquitylation compared to the non-acetylated counterpart (Supplementary Fig. 6e). Quantifying the interaction by monitoring fluorescence polarization of C-terminally fluorescein-labeled peptides revealed that the N-terminally acetylated version bound MKLN1(N447R)-FAM72A with a *K*_D_ of 13 ± 0.5 nM, whereas the non-acetylated peptide did not show detectable binding under the same conditions (Fig. 7g). The MKLN1 N447R mutant was used to prevent MKLN1 self-recruitment. To determine whether this preference extends to direct binding of full-length protein, we performed a competition fluorescence polarization assay. Full-length, N-terminally acetylated UNG2 competed with the FAM72A-bound acetylated peptide more effectively than non-acetylated full-length UNG2 (Fig. 7h).

Notably, N-terminally acetylated full-length UNG2 (expressed in insect cells) was a superior in vitro ubiquitylation substrate of CTLH^MKLN1^-FAM72A compared to non-acetylated UNG2 (expressed in bacteria) (Fig. 7i). Consistent with a specific requirement for the UNG2 N-terminal sequence itself, UNG1, the mitochondrial uracil-DNA glycosylase, which differs only by its N-terminal sequence, was not ubiquitylated by CTLH^MKLN1^-FAM72A^79^. Thus, acetylation enhances both the binding of UNG2 to FAM72A and the efficiency of its ubiquitylation.

### MKLN1 autoubiquitylation competes with other substrates

Our structural data indicated that Sub-MKLN1 occupies the same discoidin trench surface engaged by ZMYND19 and FAM72A, implying that the recruitment of either of these two proteins competes with MKLN1 self-recruitment. Superimposing the models of Sub-MKLN1-, ZMYND19-, and FAM72A-bound to the E3-MKLN1 discoidin domain confirms that all three occupy an overlapping footprint on the trench surface (Supplementary Fig. 7). To test whether this structural overlap translates into functional competition, we titrated ZMYND19 or FAM72A-UNG2 into ubiquitylation reactions containing CTLH^MKLN1^ and monitored both Sub-MKLN1 ubiquitylation and substrate ubiquitylation. Increasing concentrations of ZMYND19 progressively suppressed Sub-MKLN1 ubiquitylation while increasing ZMYND19 ubiquitylation (Fig. 7j). Similarly, titrating FAM72A-UNG2 suppressed Sub-MKLN1 ubiquitylation as UNG2 ubiquitylation increased (Fig. 7k).

A separate point of overlap is evident at the MKLN1 Kelch central channel, where the C-terminal residues of ZMYND19 and FAM72A occupy an equivalent binding position and engage the same residues in the E3-MKLN1 Kelch central channel (Fig. 6f), indicating that ZMYND19 and FAM72A share overlapping engagement surfaces with E3-MKLN1 at both the discoidin trench and the Kelch channel. Titrating ZMYND19 into a preassembled CTLH^MKLN1^-FAM72A (without UNG2) complex likewise suppressed residual autoubiquitylation of the complex while promoting ZMYND19 modification (Fig. 7l).

## Discussion

In determining cryo-EM structures representing three distinct CTLH^MKLN1^ ubiquitylation complexes, our data unveil how the combination of multiple domains from an E3 ligase receptor subunit allows a tremendous versatility of routes for substrate targeting. Despite unique features of three different substrates, they all converge on two common themes: multivalent engagement of the E3 ligase—either by the substrate itself or a substrate-binding adapter—and placement near the ubiquitylation active sites within a giant oval E3 ligase assembly (Fig. 2).

Each E3-MKLN1 dimer displays four interaction domains that altogether can be engaged in various ways (Fig. 2d). The Kelch domain Loop-1 cooperates with a particular portion of the discoidin domain trench surface to recruit the Sub-MKLN1 dimer in a 2-fold symmetric arrangement (Fig. 3). Multiple discoidin and the Kelch domain surfaces can be asymmetrically engaged, as shown by their recruiting a pair of ZMYND19 molecules (Fig. 4). Meanwhile, the discoidin trench and Kelch propeller surfaces can also mediate symmetric interactions, as during engagement of a pair of FAM72A-UNG2 complexes (Fig. 5). In addition, the UNG2 substrate is secured through its acetylated N-terminus engaging the Yippee-domain groove of FAM72A (Fig. 5c). The finding that natural substrates exploit multiple contact points distributed across MKLN1 and its binding partners has functional implications: E3 engagement at multiple sites can increase binding specificity, reinforce individually weak contacts, or constrain domain positions that would otherwise be flexibly tethered.

The structures nominate several potential MKLN1-dependent degron motifs. Internal loops from both Sub-MKLN1 and ZMYND19 display a central Asn or Asp, respectively, embedding in the MKLN1 discoidin domain trench. The second, less-resolved ZMYND19 loop that contacts the other E3 MKLN1 protomer’s discoidin also contains an Asp and an Asn residue that are candidates for making similar interactions, with potential for the loop to be arranged in the opposite orientation. Despite these similarities, the breadth of distinct substrate contacts with the MKLN1 discoidin domain allows design of E3 mutations selectively impairing association with subsets of substrates (Fig. 7c). It seems likely that many CTLH substrates will be discovered that exploit the versatility of side chains exposed in the MKLN1 discoidin domain. However, the precise sequence rules governing which loop-containing proteins are recognized by the discoidin trench remain to be defined.

In addition to their loops binding the discoidin domain, both ZMYND19 and FAM72A also project C-terminal sequences that end with an Arg that captures the MKLN1 Kelch domain (Fig. 6f, k–p). This C-terminal Arg-mediated interaction appears to drive affinity for FAM72A, because in vitro ubiquitylation of UNG2 is drastically reduced upon elimination of the FAM72A C-terminal Arg or its docking site in the MKLN1 Kelch domain, whereas there is little such effect for mutations in the MKLN1 discoidin domain. The C-terminal Arg motif was also implicated as a MKLN1-binding degron in parallel studies posted on bioRxiv during preparation of our manuscript^66,67^, and notably is also in the previously reported MKLN1 substrate AAMP^80^.

Yet another mode of substrate recruitment was resolved at high resolution in our cryo-EM structure of the MKLN1-FAM72A-UNG2 subcomplex. UNG2’s acetylated N-terminus is gripped within a Y-shaped tunnel buried inside the FAM72A Yippee domain. This architecture explains prior findings that the UNG2 N-terminal region is necessary for degradation and sufficient for FAM72A binding^53,75^, and is consistent with our peptide binding and in vitro ubiquitylation assays that demonstrated a critical role for N-terminal acetylation (Fig. 7g–i). Interestingly, the deep substrate-binding tunnel in FAM72A is established by loops wrapping around UNG2’s N-terminus. We speculate that these loops could adopt alternative, more open arrangements to engage other sequences, much like the Yippee domains of YPEL5 or CRBN^48,50,81,82^. It is possible that alternative FAM72A conformations could enable recruitment of other substrates to CTLH^MKLN1^. Irrespective of whether there are additional FAM72A-recruited substrates and how they are recognized, our structural data define how an E3 ligase can target substrates through an acetylated N-terminus. Our results also extend understanding of CTLH E3 capacity to recognize substrate N-terminal motifs, by revealing a binding mode that is mechanistically distinct from the other CTLH substrate receptor, GID4, which directly contacts the charged or polar terminus of peptides or peptidic small molecules initiating with Pro or hydrophobic residues^41,44–46^.

Beyond showing modes of CTLH^MKLN1^ substrate recognition, our data reveal how MKLN1 self-assembly mediates its ubiquitylation in a manner that competes with substrate modification. Indeed, mutations designed to selectively disrupt the E3-MKLN1–Sub-MKLN1 interface both reduced its ubiquitylation and increased the modification of substrates. Meanwhile, in our in vitro ubiquitylation assays, addition of competitor substrates ZMYND19 or FAM72A-recruited UNG2 reduced ubiquitylation of WT MKLN1 (Fig. 7j, k). Thus, MKLN1 joins the growing list of multiprotein E3 ligases that form higher-order assemblies that compete with substrates^9,83–86^. For such E3s in the cullin-RING ligase (CRL) family, substrate binding leads to activation of the E3 ligase, not only by overcoming autoinhibition, but also by directing retention of the NEDD8 modification that both protects the E3 ligase complex from disassembly and stimulates ubiquitylation activity^87,88^. Based on this precedent, we speculate that future studies could reveal additional layers of CTLH E3 regulation through formation of the MKLN1 substrate complex. For example, it is possible that the MKLN1 substrate complex could serve as a chaperone function that assists or stabilizes formation of the giant oval, or that this protects MKLN1 complexes from shuffling of CTLH subunits into complexes with WDR26 instead. The MKLN1 substrate complex may serve to protect the CTLH assembly from self-modifications that could alter activity, although availability of other binding partners may also play roles. In this regard, we note that in our cellular assay, the N447R mutant that abolishes E3-MKLN1–Sub-MKLN1 self-recruitment does not show increased ZMYND19 binding (Fig. 6h). We presume that the exogenous co-expression of MKLN1 and ZMYND19 in this setting results in preferential recruitment of ZMYND19 compared to Sub-MKLN1. The precise cellular signals that tip the balance between MKLN1 self-recruitment and engagement of other substrates, whether through post-translational modifications of Kelch Loop-1, changes in MKLN1 stoichiometry, or competition from binding partners, remain to be determined. Nonetheless, some clues to physiological context are already apparent. Degradation of both MKLN1 and ZMYND19 is stimulated by inhibition of mTORC1 signaling^46,68^, indicating that nutrient- or growth factor-dependent signaling can trigger degradation of self-recruited MKLN1 and turnover of other substrates. By contrast, FAM72A expression is highly cell type-specific and is enriched in B cells undergoing antibody diversification^53,60^. We speculate that availability of FAM72A, irrespective of competition with MKLN1 or ZMYND19, may be the primary determinant of UNG2 recruitment to CTLH^MKLN1^. Thus, cellular substrate and adapter availability, together with upstream signaling state, likely influence which mode of CTLH^MKLN1^ substrate recognition predominates in a given condition or cell type.

The variety of substrate recognition modes deployed by CTLH^MKLN1^ adds to an increasingly complex picture of degron recognition across the GID/CTLH family. The originally characterized Pro/N-degrons bind GID4^41^, while basic-aromatic internal degrons bind the WD40 domain of WDR26^48^. Heterobifunctional and molecular glue degraders can recruit substrates to GID4 and to YPEL5 bound to WDR26^50,89,90^. Ours and others’ studies^66,67^ of CTLH^MKLN1^ now extend this repertoire to C-terminal Arg degrons, while our study also reveals recognition of Asp/Asn-loop internal motifs, and adapter-mediated recognition of an acetylated N-terminus. Perhaps the most striking feature of CTLH E3 assemblies is that the diverse modes of substrate recruitment all allow placement of substrates within the hollow oval E3, proximal to the active sites of the CTLH-specific E2 UBE2H. This distinctive E3 geometry—which allows substrate receptors to be localized at various positions along the oval and yet place substrates in the center—contrasts with the more constrained placement of substrate-binding modules in the Cullin-RING ligase family^87^, yet may serve a similar function: ensuring that diverse degron-bearing substrates are reliably positioned for ubiquitylation. Such plug-and-play E3 ligase mechanisms may be particularly suitable for targeted protein degradation.

## Methods

### Cloning, protein expression and purification

All constructs in this study were generated using standard molecular biology techniques. Mutant constructs were either generated by Gibson Assembly or by QuikChange Site-Directed Mutagenesis. Oligonucleotide sequences for mutational analyses are provided in Supplementary Data 1. Plasmid sequences were verified through Sanger sequencing, and purified proteins were confirmed by intact mass analysis.

#### Insect cell expression and purification

All CTLH E3 complexes, subcomplexes, E1 enzyme UBA1, MKLN1 in isolation, FAM72A, UNG2, MKLN1(N447R)-FAM72A, and FAM72A-UNG2 were expressed in *Trichoplusia ni* High Five insect cells (Thermo Fisher Scientific, catalog no. B85502) following three rounds of baculovirus amplification in Sf9 cells (Thermo Fisher Scientific, catalog no. 11496016). Core CTLH components for insect cell expression (RANBP9- TWA1-MAEA-RMND5A) or (RANBP9- TWA1-MAEA-RMND5A-ARMC8 used in Fig. 1d), and CTLH^MKLN1^-FAM72A (used in Figs. 7i and 7l)were combined into single baculoviral expression vectors with the bigBac assembly method^91^. To generate supramolecular assemblies, untagged MKLN1 WT or mutants, or WDR26 were co-expressed with the core-CTLH. Cell pellets were resuspended on ice in lysis buffer 50 mM Tris pH 8.0, 150 mM NaCl, 5 mM DTT supplemented with protease inhibitors (1 µM pepstatin, 10 µg/mL aprotinin, 5 µg/mL leupeptin, and 1 mM AEBSF). Cells were lysed by sonication, and the lysate was cleared by centrifugation (20,000 × *g*, 30 min). All assemblies of CTLH used in this study contained a Twin-Strep tag on the TWA1 C-terminus and were affinity-captured by Strep-Tactin resin (IBA Life Sciences) for 60 min^15,48,50^. The eluted protein complexes were subjected to size-exclusion chromatography (SEC) using a Superose 6 Increase 10/300 GL column (Cytiva) in the final buffer containing 25 mM HEPES, pH 7.5, 150 mM NaCl, 1 mM DTT (SEC buffer) (or 5 mM DTT for Cryo-EM). FAM72A WT and mutant versions used for ubiquitylation assays carried an N-terminal Twin-Strep tag. During expression, cells were supplemented with 50 µM ZnCl_2_ and were purified by affinity capture on Strep-Tactin resin for 60 min. Proteins were eluted from the resin by overnight cleavage with HRV3C protease at 4 °C and further purified using an anion exchange HiTrap Q HP column (Cytiva) and eluted along the salt gradient 50–1000 mM NaCl in 50 mM Tris pH 8.0, 5 mM DTT (Q buffer), followed by SEC using Superdex 75 Increase 10/300 GL in SEC buffer. WT and mutant MKLN1 proteins in isolation or co-expressed with untagged FAM72A (as in the case of MKLN1 N447R used in Fig. 7g) were expressed as N-terminal GST fusion proteins, captured by GST affinity chromatography (Cytiva) and eluted by overnight incubation with TEV protease at 4 °C. Proteins were further purified by anion exchange in a Q buffer gradient and eluted over a Superose 6 column in SEC buffer. E1 UBA1 protein was purified analogous to MKLN1, but eluted over a Superdex 200 10/300 GL column (Cytiva) in SEC buffer. UNG2 was fused to a 6x C-terminal His-Tag and purified in isolation or co-expressed with untagged FAM72A (supplemented with 50 µM ZnCl_2_). The cell pellet was resuspended in 50 mM Tris pH 8.0, 150 mM NaCl, 5 mM DTT, 10 mM imidazole, captured by His-affinity chromatography (Cube Biotech), eluted with 300 mM imidazole, then subjected to cation exchange on a HiTrap SP HP column (Cytiva) and eluted along the salt gradient 50–1000 mM NaCl in 50 mM MES pH 6.5, 5 mM DTT (S buffer). The protein was then passed through a Superdex 200 column in SEC buffer.

#### Bacterial cells expression and purification

Proteins used for ubiquitylation assays (E2 enzyme UBE2H, substrate receptor GID4, substrate ZMYND19, ubiquitin K0, UNG1, and unacetylated UNG2), proteins used in fluorescence polarization assays (unacetylated UNG2, and MKLN1 Kelch propeller), as well as proteins used for cryo-EM sample preparation (UBE2H C87K and ubiquitin for E2~UB stable mimic) were expressed in E. coli Rosetta cells. Cultures were grown in Terrific Broth with antibiotics at 37 °C until (OD)_600_ of 0.6. The temperature was then lowered to 18 °C, and expression was induced using 0.2 mM IPTG, and grown overnight. For ZMYND19 expression, the induction was also supplemented with 200 µM ZnCl_2_. UBE2H E2 enzyme was co-expressed with a catalytic subunit of the kinase CK2 (CK2α), as described previously, to fully phosphorylate the C-terminal UBE2H tail^72^. Cells were pelleted by centrifugation (4500 × *g*, 15 min) and resuspended in lysis buffer containing 50 mM Tris pH 8.0, 150 mM NaCl, 5 mM DTT, 1 mM PMSF. GID4 and WT ubiquitin lysis buffers were supplemented with 5 mM imidazole. Cells were then sonicated, and lysates were cleared by centrifugation (20,000 × *g*, 45 min). UBE2H (WT or C87K) and all versions of ZMYND19 were expressed as N-terminal GST fusions with a TEV cleavage site, whereas MKLN1 Kelch propeller (res. 246-627) WT and mutants contained an HRV3C cleavage site in place of TEV. These purifications were analogous to insect cell purification steps of GST-tagged proteins. GID4 (∆1-99) was fused to a 6x C-terminal His-Tag, whereas ubiquitin was fused to a 6x N-terminal His-Tag with an HRV3C cleavage site. After purification by anion exchange in a Q buffer gradient, all three proteins were eluted by size-exclusion chromatography on a HiLoad 16/600 Superdex 75 column (Cytiva). UNG1 and UNG2 purification steps were analogous to UNG2 purification from insect cell expression. K0 ubiquitin (all Lys residues mutated to Arg to prevent polyubiquitin chain formation) was expressed with an N-terminal GST tag followed by an HRV3C cleavage site and a single cysteine residue immediately upstream of the ubiquitin sequence to enable fluorescent labeling. The protein was purified by GST affinity chromatography (Cytiva), eluted by overnight cleavage with HRV3C protease at 4 °C, followed by size-exclusion chromatography on a HiLoad 16/600 Superdex 75 column (Cytiva).

#### Fluorescent labeling—ubiquitin (*K0)

Cys-UB K0 (0.8 mM) was buffer-exchanged using a PD MidiTrap G-25 column (Cytiva, 28918008) into 20 mM HEPES, 200 mM NaCl, and incubated with 10x molar excess (8 mM) of Fluorescein-5-maleimide (5-MF) for 2 hr at RT. The reaction was then quenched with 10 mM DTT, and the protein was then desalted using a PD MidiTrap G-25 column in 20 mM HEPES, 200 mM NaCl, and finally subjected to size-exclusion chromatography on Superdex 75 in SEC buffer.

#### Preparation of stable mimic of E2~ubiquitin conjugate

A stable mimic of the E2~ubiquitin intermediate was prepared by substituting the catalytic cysteine of UBE2H with lysine (C87K), enabling conjugation of 6xHis-3C-ubiquitin to the lysine side chain via an isopeptide bond as described previously^72^. The E2 loading reaction contained 3 µM E1 UBA1, 100 µM UBE2H C87K, and 100 µM 6xHis-3C-ubiquitin in loading buffer (25 mM Bis-Tris propane pH 9.5, 100 mM NaCl, 2.5 mM MgCl_2_, 1 mM ATP). Following overnight incubation at 37 °C, the reaction mixture was subjected to size-exclusion chromatography in 25 mM Tris pH 8.0, 150 mM NaCl, 5 mM imidazole, 1 mM DTT to remove excess ubiquitin and E1. The UBE2H~ubiquitin conjugate was then affinity-captured by His-affinity chromatography for 30 min to remove unreacted UBE2H, followed by SEC on Superdex 75 in 25 mM HEPES, pH 7.5, 150 mM NaCl, 1 mM DTT.

### In vitro ubiquitylation assays

All ubiquitylation assays were performed in the reaction buffer containing 25 mM HEPES, pH 7.5, 150 mM NaCl,0.25 mM DTT, 5 mM ATP and 10 mM MgCl_2_ at room temperature. Reactions were quenched by adding Laemmli buffer containing β-mercapthoethanol. The assays were performed by mixing 0.2 μΜ UBA1, 1 μΜ UBE2H, 0.5 μΜ CTLH^MKLN1^ complex, (supplemented with 1 μΜ GID4 (∆1-99) for Fig. 1d). For assays assessing UNG2 ubiquitylation (Figs. 1e, 6c, g, p and 7c, i), FAM72A was pre-incubated with UNG2 at 0.5 μΜ each, prior to being added to the reaction mixture. For assays assessing ZMYND19 ubiquitylation, ZMYND19 was added to the mixture at 0.5 μΜ. The reaction was started by adding 10 μΜ of fluorescently labeled ubiquitin *K0 and quenched after 15 min. An assay assessing the importance of N-terminal acetylation of UNG2 was performed using 0.5 μΜ CTLH^MKLN1^ -FAM72A complex expressed as a single baculovirus (see “Insect cell expression and purification”). Synthesized peptides containing the 25 N-terminal residues of UNG2 contained either N-terminally acetylated Met or unacetylated Met (synthesized in-house, purified by HPLC to >90% purity). Acetylated and nonacetylated UNG2 peptides were used at both 50 and 20 μΜ. Following pre-incubation of peptides with 0.5 μΜ CTLH^MKLN1^-FAM72A, 0.5 μΜ UNG2 was added and reaction was started with 10 μΜ of fluorescently labeled ubiquitin *K0, and quenched after 5 min (Supplementary Fig. 6e). To assess the requirement for the UNG2 N-terminal sequence, ubiquitylation reactions were assembled as above using 0.5 μΜ CTLH^MKLN1^-FAM72A, with N-terminally acetylated UNG2 (expressed in insect cells), non-acetylated UNG2 (expressed in bacteria), or UNG1 added at increasing concentrations (0.125 μΜ, 0.25 μΜ, 0.5 μΜ; calculated as a dimer to account for two UNG2 binding sites on each side of the CTLH^MKLN1^-FAM72A assembly). The reaction was started by adding 10 μΜ of fluorescently labeled ubiquitin *K0 and quenched after 15 min (Fig. 7i).

To test competition between Sub-MKLN1 and ZMYND19 or FAM72A-bound UNG2, increasing concentrations of the indicated competitor were pre-incubated with the CTLH^MKLN1^ complex for 30 min prior to initiating the reaction by adding 10 μΜ of fluorescently labeled ubiquitin *K0, and quenching after 30 min. To test whether ZMYND19 competes with Sub-MKLN1 for ubiquitylation (Fig. 7j), ZMYND19 (0.125–2 μΜ, calculated as a dimer to account for the two ZMYND19-binding sites on each side of the CTLH^MKLN1^ assembly) was pre-incubated with 0.5 μΜ CTLH^MKLN1^. An analogous assay was performed using FAM72A-UNG2 (0.125–6 μΜ, co-expressed; see “Insect cell expression and purification”) in place of ZMYND19 (Fig. 7k), and pre-incubated with 0.5 μΜ CTLH^MKLN1^. To test whether ZMYND19 competes with residual autoubiquitylation of a pre-assembled CTLH^MKLN1^-FAM72A complex (Fig. 7l), increasing concentrations of ZMYND19 (0.125–4 μΜ, calculated as a dimer) were pre-incubated with 0.5 μΜ CTLH^MKLN1^-FAM72A. Ubiquitylation was visualized by a fluorescence scan of SDS–PAGE gels using the Amersham Typhoon Imager 600 (GE Healthcare).

### Fluorescence polarization assays

#### Quantification of MKLN1 Kelch domain–C-terminal peptide interactions

To quantify binding of C-terminal Arg-containing peptides to the MKLN1 Kelch domain (Fig. 6k), we used an FP assay monitoring association of fluorescein-labeled ZMYND19 or FAM72A C-terminal peptides with the MKLN1 Kelch domain. Peptides corresponding to the 19 C-terminal residues of ZMYND19 or FAM72A, labeled with fluorescein at the N-terminus, were synthesized in-house, purified by HPLC to >90% purity, and confirmed by mass spectrometry. To set up binding reactions, a 2.5-fold serial dilution series of the MKLN1 Kelch domain (WT, W291A/G273W, or W291A/Y346A) in the binding buffer (25 mM HEPES pH 7.5, 150 mM NaCl, 1 mM DTT, 1 mg/ml BSA, and 0.1% Tween-20) was prepared, starting at 50 μM. 27 μL of each dilution point was combined with 3 μL of 100 nM labeled peptide prepared in 10% DMSO, yielding final concentrations of 50 μM Kelch domain and 10 nM peptide in 1% DMSO at the first titration point, and the mixtures were transferred to 384-well flat-bottom black plates (Greiner). Fluorescence polarization was determined by measuring the parallel and perpendicular components of fluorescence emission, following excitation at 482 nm and detection at 530 nm, on a CLARIOstar microplate reader (BMG Labtech). Polarization values from three independent experiments were plotted using GraphPad Prism (version 10.4.1) and fit by nonlinear regression to a one-site binding model. A dissociation constant (*K*_D_) was derived only for the interaction between the ZMYND19 C-terminal peptide and WT MKLN1 Kelch domain, as binding of the other peptide-mutant combinations did not reach saturation under the conditions tested.

#### Quantification of MKLN1-FAM72A binding to the UNG2 N-terminal peptide

An analogous FP assay was used to quantify binding of the acetylated or non-acetylated UNG2 N-terminal peptides (Fig. 7g), synthesized and labeled with fluorescein at the C-terminus (in-house, as described above), to MKLN1(N447R)-FAM72A (co-expressed in insect cells; see “Insect cell expression and purification”). N447R mutant was used to prevent MKLN1 self-recruitment. A twofold serial dilution series of MKLN1(N447R)-FAM72A was prepared in the binding buffer (25 mM HEPES pH 7.5, 150 mM NaCl, 1 mM DTT, 1 mg/ml BSA, and 0.1% Tween-20), with a final concentration of 0.5 μM at the first titration point, combined with the labeled peptide at a final concentration of 10 nM in 1% DMSO. Fluorescence polarization was measured as described above, and data were analyzed by nonlinear regression using a one-site binding model. A dissociation constant (*K*_D_) was obtained only for the acetylated UNG2 peptide, since the non-acetylated UNG2 peptide showed no detectable binding.

#### Competitive FP assay probing full-length ZMYND19 engagement of the MKLN1 Kelch domain

To test whether full-length ZMYND19 competes with the labeled ZMYND19 C-terminal peptide for MKLN1 Kelch domain binding, we monitored the propensity of unlabeled full-length ZMYND19 (WT or ΔC-term Arg) to displace the peptide (Supplementary Fig. 5d). A premix of 250 nM MKLN1 Kelch domain and 20 nM labeled ZMYND19 peptide in 2% DMSO was prepared in the binding buffer (25 mM HEPES pH 7.5, 150 mM NaCl, 1 mM DTT, 1 mg/ml BSA, and 0.1% Tween-20). A twofold serial dilution series of full-length ZMYND19 (WT or ΔC-term Arg), starting at 160 μM, was combined with an equal volume of the peptide-Kelch premix, yielding final concentrations of 125 nM Kelch domain and 10 nM peptide in 1% DMSO at each titration point, with full-length ZMYND19 present at a starting concentration of 80 μM and diluted twofold thereafter. A control well containing the peptide-Kelch premix diluted 1:1 with binding buffer alone (125 nM Kelch domain, 10 nM peptide, 1% DMSO), without full-length competitor, was included to define the uncompeted polarization signal. Polarization values from three independent experiments were plotted using GraphPad Prism (version 10.4.1) and fit to a log(inhibitor) versus response–variable slope (four parameters) model.

#### Competitive FP assay probing full-length UNG2 engagement of FAM72A

To test whether full-length UNG2 (N-terminally acetylated, expressed in insect cells, or non-acetylated, expressed in bacteria) competes with the labeled acetylated UNG2 N-terminal peptide for binding to FAM72A, we monitored the propensity of unlabeled full-length UNG2 to displace the peptide (Fig. 7h). A twofold serial dilution series of full-length UNG2 (acetylated or non-acetylated), starting at a 2× concentration of 100 μM, was combined with an equal volume of a premix containing 250 nM FAM72A and 20 nM labeled acetylated UNG2 peptide in 2% DMSO, yielding final concentrations of 125 nM FAM72A, 10 nM peptide, and 50 μM full-length UNG2 in 1% DMSO at the first titration point. Polarization values from three independent experiments were plotted using GraphPad Prism (version 10.4.1) and fit to a log(inhibitor) versus response—variable slope (four parameters) model.

### Mass photometry

WT and mutant MKLN1 were analyzed using mass photometry to determine their oligomeric states. The mass photometer was calibrated by running a standard containing a protein mixture of different masses (Conalbumin, Aldolase, Ferritin, Thyroglobulin), each at the final concentration of 20 nM. Purified MKLN1 proteins were measured at the final concentration of 20 nM (calculated for monomeric MKLN1) in 25 mM HEPES, pH 7.5, 150 mM NaCl, 1 mM DTT. Data were collected on a Refeyn TwoMP mass photometer using Refeyn AcquireMP v.2.3.0. Data were analyzed using the Refeyn DiscoverMP v.2.3.0 software.

### Single particle cryo-EM

#### Cryo-EM sample preparation

CTLH complexes (CTLH^MKLN1^, CTLH^MKLN1^-ZMYND19, CTLH^MKLN1^-FAM72A-UNG2) or subcomplexes (ZMYND19-MKLN1-RANBP9-TWA1 and FAM72A-UNG2-MKLN1-RANBP9-TWA1) used for structural studies were expressed as a single baculovirus in insect cells using methods described above (see “Insect cell expression and purification”). Subcomplexes containing either FAM72A-UNG2 or ZMYND19 were supplemented with 50 μΜ ZnSO_4_. Each complex was purified by Strep-Tactin affinity chromatography (Twin-Strep tag on TWA1 C-terminus) followed by size-exclusion chromatography on a Superose 6 Increase 10/300 GL column (Cytiva) in the final buffer containing 25 mM HEPES, pH 7.5, 150 mM NaCl, 5 mM DTT. CTLH complexes were then mixed with 8 molar excess of UBE2H~ubiquitin stable mimic, incubated on ice for 30 min, and sized on Superose 6 in the same SEC buffer as above. The fractions containing UBE2H~ubiquitin bound to the complex were pooled and concentrated. All three CTLH^MKLN1^-UBE2H~ubiquitin complexes were then concentrated to 2.7 mg/ml. Just before plunging, 0.1% octyl-β-glucoside detergent (Sigma-Aldrich) was added to each complex. Three subcomplexes were concentrated to 3 mg/ml and supplemented with the same detergent at 0.1% final concentration. Cryo-EM grids were prepared using copper holey-carbon grids (R1.2/1.3, 200 mesh, Quantifoil), glow-discharged prior to use. 3.5 µl of sample was applied to the grids and incubated, then blotted using Whatman No. 1 filter paper (blot force: 3, blot time: 3 s) and vitrified by plunge-freezing into liquid ethane using a Vitrobot Mark IV (Thermo Fisher Scientific) operated at 4 °C and 100% humidity.

#### Data collection

Cryo-EM data collection details are listed in Supplementary Table 1, and data processing workflow schemes are shown in Supplementary Figs. 8–14. A screening dataset was recorded using SerialEM (v4.2.1) for each sample either on a Glacios or Arctica transmission electron microscope (Thermo Fisher Scientific) operated at 200 kV, equipped with a Gatan Vista Alpine or Falcon III direct electron detector. High-resolution datasets and lower-resolution CTLH^MKLN1^-ZMYND19, CTLH^MKLN1^-FAM72A-UNG2 complexes were recorded on a Titan Krios transmission electron microscope (Thermo Fisher Scientific) operated at 300 kV, equipped with a Gatan K3 Summit direct electron detector operating in counting mode. Automated data acquisition was carried out using SerialEM^92^.

#### Data processing

The CTLH^MKLN1^-UBE2H~ubiquitin complex dataset was processed in RELION 5.0 (beta 4)^93,94^. Raw video frames were motion-corrected with dose-weighting using MotionCor2^95^ followed by contrast transfer function (CTF) estimation using CTFFIND4^96^, both implemented in RELION 5.0 (beta 4). Particles were automatically picked using Gautomatch (K. Zhang) with an initial model generated from the screening dataset as a template. The extracted particles were directly subjected to three rounds of unmasked 3D classification, which revealed heterogeneity in catalytic module conformations. To further classify different conformations of catalytic modules, a 3D classification without alignment and a mask over a catalytic module was performed. To obtain high-resolution details of MKLN1, masks were generated around each MKLN1 tetramer positioned on either side of the CTLH assembly, and signal subtraction was performed to isolate the corresponding densities and enrich the particle number. The signal-subtracted particle pools from each side were joined and subjected to 3D classification and refinement with a mask over the MKLN1 tetramer. Focused 3D classification without particle alignment was then applied to enrich for particles with the most complete and well-resolved features, while masking out more flexible parts. This was followed by successive rounds of local refinement to yield the final high-resolution reconstructions. The map was then post-processed by B-factor sharpening, and final resolution is reported based on the gold-standard Fourier shell correlation at the 0.143 cutoff criterion. The data processing workflow is outlined in Supplementary Fig. 8. The remaining datasets were processed in CryoSPARC (v. 4.7.1)^97^. Particles were picked in CryoSPARC using either a template generated from CryoSPARC Live processing or an initial model obtained by ab initio reconstruction, as shown in processing schemes (Supplementary Figs. 9–14). Where indicated in the scheme, a dedicated “junk” class was included as input volume during classification to remove low-quality particles. To further improve map quality, a global CTF, local CTF, and Reference Motion Correction were performed once particles were classified and re-extracted to full pixel size. To remove flexible parts of the sample, particle subtraction was performed over the more flexible parts of the ZMYND19 and FAM72A-UNG2-MKLN1-RANBP9-TWA1 subcomplex. For the ZMYND19-MKLN1 subcomplex, a flexible Kelch propeller (assigned as E3-MKLN1-A in this study) was signal-subtracted from the subcomplex. Then, various masks over different areas were used to obtain high-resolution details by performing 3D classification and local refinements, similar to the approach taken for MKLN1 dataset processing described above. For the final local refinements of the ZMYND19-containing subcomplex, three additional passes through the data beyond the point of GS-FSC resolution convergence were applied to maximize map quality. All final high-resolution maps were post-processed using DeepEMhancer^98^. Data processing workflows are outlined in Supplementary Figs. 9–11. For the CTLH^MKLN1^-UBE2H~ubiquitin complex with FAM72A-UNG2 whole circle and FAM72-UNG2 bound to E3-MKLN1 discoidin, a series of classifications and refinements were performed, without signal subtraction. To obtain the FAM72A C-terminus bound to MKLN1 Kelch propeller, the CTLH^MKLN1^-UBE2H~ubiquitin complex with the FAM72A-UNG2 whole-circle dataset was further processed, and a global CTF, local CTF, and Reference Motion Correction were performed once particles were re-extracted to a full pixel size. Parts of the map were then signal-subtracted, followed by a series of local refinements over the FAM72A-MKLN1-RANB9-TWA1 subcomplex. The final high-resolution map was post-processed using DeepEMhancer^98^. The processing workflows are outlined in Supplementary Figs.12–14.

#### Model building and analysis

Atomic models were built by first docking the AlphaFold3^99^ predictions into the post-processed maps, followed by iterative cycles of manual model building in Coot-1.1.20^100^ and real-space refinement in Phenix^101^. PDB 7NSC^15^ was additionally used as an initial reference for the RANBP9 and TWA1 subunits in the ZMYND19 and FAM72A subcomplex datasets. The refinement and validation statistics are listed in Supplementary Table 1. The configuration of the zinc-binding sites within FAM72A and ZMYND19 was restrained during each Phenix refinement. For the ZMYND19-MKLN1-RANBP9-TWA1 subcomplex, initial models were manually built into the corresponding high-resolution maps (see Supplementary Fig. 10) as described above. Then, a composite map was generated using the VOP maximum command in UCSF ChimeraX. Individual models built from local maps were then fitted into the composite map, and the final model was refined in Phenix against the composite map. All maps used in this study have been deposited. Low-resolution maps used in this study are indicated by boxes in the processing schemes (Supplementary Figs. 8–14). All ZMYND19 high-resolution densities shown in Fig. 4 come from individual maps. Structural analyses and visualization were performed in ChimeraX^102^.

### Cellular assays

#### Cell culture maintenance

Flp-In T-Rex HEK293 cells (ATCC CRL-1573) were purchased from Thermo Fisher Scientific (catalog no. R78007). Cells were maintained in DMEM (Gibco) supplemented with 10% (v/v) fetal bovine serum (Gibco), GlutaMAX (Gibco), penicillin (100 U/ml), streptomycin (0.1 mg/ml), Zeocin (100 µg/ml), and Blasticidin S HCl (15 µg/ml) (Gibco). Cultures were grown at 37 °C in a humidified atmosphere containing 7% CO₂. Cultures were regularly checked for the absence of mycoplasma contamination.

#### Knockout generation

MKLN1−/−, ZMYND19−/−, and MKLN1−/−ZMYND19−/− knockout cell lines were generated using a CRISPR–Cas9(D10A) nickase-based genome editing approach. Paired guide RNAs targeting two loci on the sense and antisense strands within a 20-base-pair vicinity were used. For MKLN1, the target locus was on exon 4, and for ZMYND19 on exon 3. The guides were designed using the ATUM gRNA design tool. Sense and antisense gRNA sequences followed by the PAM sequence were cloned into the pBABED-U6-Puromycin plasmid (a gift from Thomas Macartney, University of Dundee, UK) and pX335-Cas9(D10A) (Addgene), respectively. Two pairs of complementary primers have been used for each knockout, i.e., for MKLN1, guide 01 CACCGCCAGCTTTAACTTTAGCATC (forward primer) and AAACGATGCTAAAGTTAAAGCTGGC (reverse primer); guide 02 CACCGAGGACAAGAGTGGAACTTTA (forward primer) and AAACTAAAGTTCCACTCTTGTCCTC (reverse primer). Similarly, for ZMYND19, guide 01 CACCGCAACCTGCAACTGGTGCCGT (forward primer) and AAACACGGCACCAGTTGCAGGTTGC (reverse primer); guide 02 CACCGTGTCCACGGTCACAGCGTTG (forward primer) and AAACCAACGCTGTGACCGTGGACAC (reverse primer). The complementary guide primer pairs were diluted to 2 µM and were annealed by mixing and heating them to 95 °C for 5 min and letting them cool to RT. The annealed primers were ligated into Bbs1-digested above-mentioned plasmids using the manufacturer’s protocol from Roche (11635379001). Flip-In T-Rex HEK293 cells were co-transfected with the corresponding sense and antisense gRNA constructs of 2.5 μg each using Lipofectamine 3000 (Invitrogen) according to the manufacturer’s instructions. After 24 h, transfected cells were subjected to puromycin selection (2 µg/ml) for 48 h, followed by expansion and single-cell cloning by serial dilution. Knockout clones were confirmed by immunoblot analysis and sequencing of the targeted genomic regions (Supplementary Fig. 15).

Successful knockout clones were verified by genomic sequencing of the target locus. Briefly, the genomic DNA was extracted from 0.5-1 million cells of single-cell-expanded knockout clones using the DNeasy Blood & Tissue Kit (Qiagen, catalog no. 69504) following the manufacturer’s protocol. Further 50 ng of genomic DNA was used as a template for PCR amplification of target loci. The PCR product was cloned into the pCR-Blunt II-TOPO vector using Zero Blunt TOPO PCR Cloning Kit (Invitrogen, catalog no. K280002) and verified by Sanger sequencing using M13 standard primer and aligned to MKLN1 and ZMYND19 target loci, respectively (Supplementary Fig. 15).

#### Transient transfections

For transient transfections, N-terminally 3xFlag tagged MKLN1 (wildtype; W291A,G273W; N447R; Q43A,Y30A,N144A,R46A; Q43A,Y30A,N144A,R46E; Y30A,H80A,F143A), or N-terminally HA tagged ZMYND19 (wildtype; ∆C-ter Arg; ∆C-ter 3 res.; ∆44-47 > GS (40s-loop> GS) ∆153-156>GS(150s-loop>GS); D45A,D155A; D45K,D155K) or untagged FAM72A (wildtype) were cloned into pcDNA3.1 vector using standard molecular biology techniques and constructs were verified by sanger sequencing. These plasmids were transiently expressed in different cellular backgrounds as depicted in the figures. Briefly, HEK293 Flip-In cells were seeded in a 6-well plate at 0.5 million cells per well. After 24 h, maintenance media was replaced with serum-free media without antibiotics (DMEM (Gibco) supplemented with GlutaMAX (Gibco)), penicillin (100 U/ml), streptomycin (0.1 mg/ml). Each well is transfected with 1 μg per plasmid, either alone or in combination as described in the figures, using 7.5 µl of Polyethylenimine (PEI) (stock of 1 mg/ml dissolved in 0.2 M HCl) in 130 µl serum free media, followed by supplementing 10 % serum to final volume after 3 h of transfection and cultured for 24 h before proceeding to other treatments and downstream applications.

#### Inhibitor treatments

Flip-In T-Rex-HEK293 cell lines of knockouts and/or transient transfections underwent either treatments with 0.5 μM mTOR inhibitor, Torin1 (Cell Signaling Technology, 14379S, stock: 1 mM in DMSO) or co-treatment of 1 µM proteasome inhibitor, MG132 (Sigma-Aldrich, M8699, stock: 42 mM in DMSO) together with Torin1 or control treatment with DMSO (equal volumes) for 16 h followed by cell lysis and other downstream experiments as indicated in figures.

#### Lysis, SDS-PAGE and Western blotting

Flp-In T-Rex-HEK293 cell lines were washed once with PBS, lysed in RIPA buffer 1x (Cell Signaling, 9806), supplemented with HALT protease/phoshotase inhibitor cocktail (Thermo Fisher Scientific, 78444), Benzonase 10 U/ml (Millipore), and homogenized by pushing 10× through a 22 G syringe. Lysates were cleared by centrifugation at 20,000 × *g* for 20 min at 4 °C, and protein concentration was determined by Micro BCA-Protein Assay (Thermo Fisher Scientific, 23235). Lysates were mixed with Laemmli sample buffer and heated for 10 min at 95 °C for protein denaturation. For immunoblot analysis, equal amounts of denatured lysates were separated on SDS-PAGE, and proteins from gels were transferred onto 0.2 µm methanol-activated PVDF membrane (Cytiva) and visualized by immunoblotting using the indicated primary antibodies overnight at 4 °C followed by incubation with corresponding secondary HRP-conjugated antibodies for 30 min at room temperature. The following primary antibodies were used: Mouse monoclonal anti-ZMYND19 (Santa Cruz # sc-398514) Rabbit polyclonal anti-RANBP9 (Abnova # PAB16671), Mouse monoclonal anti-ARMC8 (Santa Cruz # sc-365307), Mouse monoclonal anti-MKLN1 (Santa Cruz # sc-398956), Rabbit polyclonal anti-RANBP9 (Proteintech # 17755-1-AP), Mouse monoclonal Anti-Ubiquitinylated proteins clone FK2 (Millipore # 04-263), Rabbit monoclonal β-Actin (13E5(Cell Signaling Technology # 4970)), Rabbit monoclonal Vinculin [EPR8185] (Abcam # ab129002), Mouse monoclonal UNG (OTI2C12) (Thermo Fisher Scientific # MA525686), Mouse monoclonal ANTI-FLAG M2 (Sigma-Aldrich # F1804), Mouse monoclonal HA-Tag (6E2) (Cell Signaling Technology # 2367), Rabbit Ponoclonal FAM72A (Thermo Fisher Scientific # PA583650). The following secondary HRP-conjugated antibodies were used: Goat anti-Mouse IgG (H + L) Secondary Antibody (Thermo Fisher Scientific # 31430), Goat anti-Rabbit IgG (H + L) Secondary Antibody (Thermo Fisher Scientific # 31460). Immunoblots were developed using Clarity Western ECL Substrate (Bio-Rad, 1705061) and imaged using Amersham Imager 600/800 (GE Life Sciences). All primary antibodies were used at a 1:1000 dilution, whereas the secondary antibodies were used at 1:5000.

#### Ranbp9 immunoprecipitation

For immunoprecipitation of CTLH-associated proteins, 1 µg of Ranbp9 antibody (Proteintech, 17755-1-AP) was incubated overnight at 4 °C with equal amounts of 0.5–1 mg of cell lysates on a roller. This was followed by incubation of samples with 20 µl of Pierce^TM^ Protein A/G Magnetic beads (Thermo Fisher Scientific, 88803) for 2 h at 4 °C on a roller. All immunoprecipitation reactions were extensively washed in lysis buffer to remove nonspecific binding. Immunoprecipitated proteins were eluted by boiling in reducing SDS sample buffer and separated by SDS-PAGE gel, followed by immunoblotting using indicated antibodies.

#### Quantification and statistical analysis of Western blots

For the semi-quantitative assessment of cellular protein amounts, MKLN1, ZMYND19, UNG2, and the loading controls (Actin and Vinculin) were visualized by immunoblot analysis and imaged using Amersham Imager 800 (GE Life Sciences). Signals of immunoblots were quantified using ImageJ software and normalized to loading controls. Additional normalization of the signals was performed with control (DMSO) treatments either within their respective transfected proteins or with WT transfected proteins, as stated in the figures. For statistical analysis, at least three biological replicates were considered, and two-way ANOVA with Dunnett’s multiple comparison tests were performed and plotted in PRISM v10.4.0 (GraphPad).

## Supplementary information

Supplementary Information

Supplementary Data 1

Transparent Peer Review file

## Source data

Source data

## Data Availability

The structural data and volumes for MKLN1 obtained from CTLH^MKLN1^ are available from the RCSB-PDB and EMDB with the identifiers: PDB 29UE, EMD-57377, EMD-57376, EMD-57378. Cryo-EM maps of ZMYND19-bound MKLN1 in complex and subcomplex, each determined in two distinct assembly states, are available under accession codes: EMD-57380, EMD-57379, EMD-57381, EMD-57382. Consensus map for a composite map, and all volumes used for high-resolution analysis and composite map generated by these volumes are available as: EMD-56913, EMD-56909, EMD-56910, EMD-56911, EMD-56912. Composite map and atomic coordinates are available with the identifiers: EMD-57385, PDB 29UG. Maps of FAM72A-UNG2-bound MKLN1 assemblies are available under EMD-57383, EMD-57384. Atomic coordinates and structural data for the FAM72A-UNG2-discoidin heterodimer are available as: PDB 29UI, EMD-57387. FAM72A C-terminus-bound MKLN1 complex atomic coordinates and structural data are available as: PDB 31TM, EMD-58652. The PDB coordinates and maps used for fitting and comparison with the structures and maps reported here have been previously published and are publicly available under the following accession codes: PDB 8PJN, PDB 7NSC, PDB 8QBN, PDB 9NR3, PDB 9RSD; EMD-12541, EMD-17705, EMD-12542, EMD-18174. Source data are provided with this paper.
