# Supplementary material for: Multimodal substrate recruitment enables CTLHMKLN1 E3 ligase to target acetylated N-, C-, and internal degrons
Source: Nat Commun. 2026 Oct 17;17:10877. doi: 10.1038/s41467-026-78311-4 (PMC13676621; doi:10.1038/s41467-026-78311-4)
Supplement: Supplementary file 1 [file 41467_2026_78311_MOESM1_ESM.pdf]

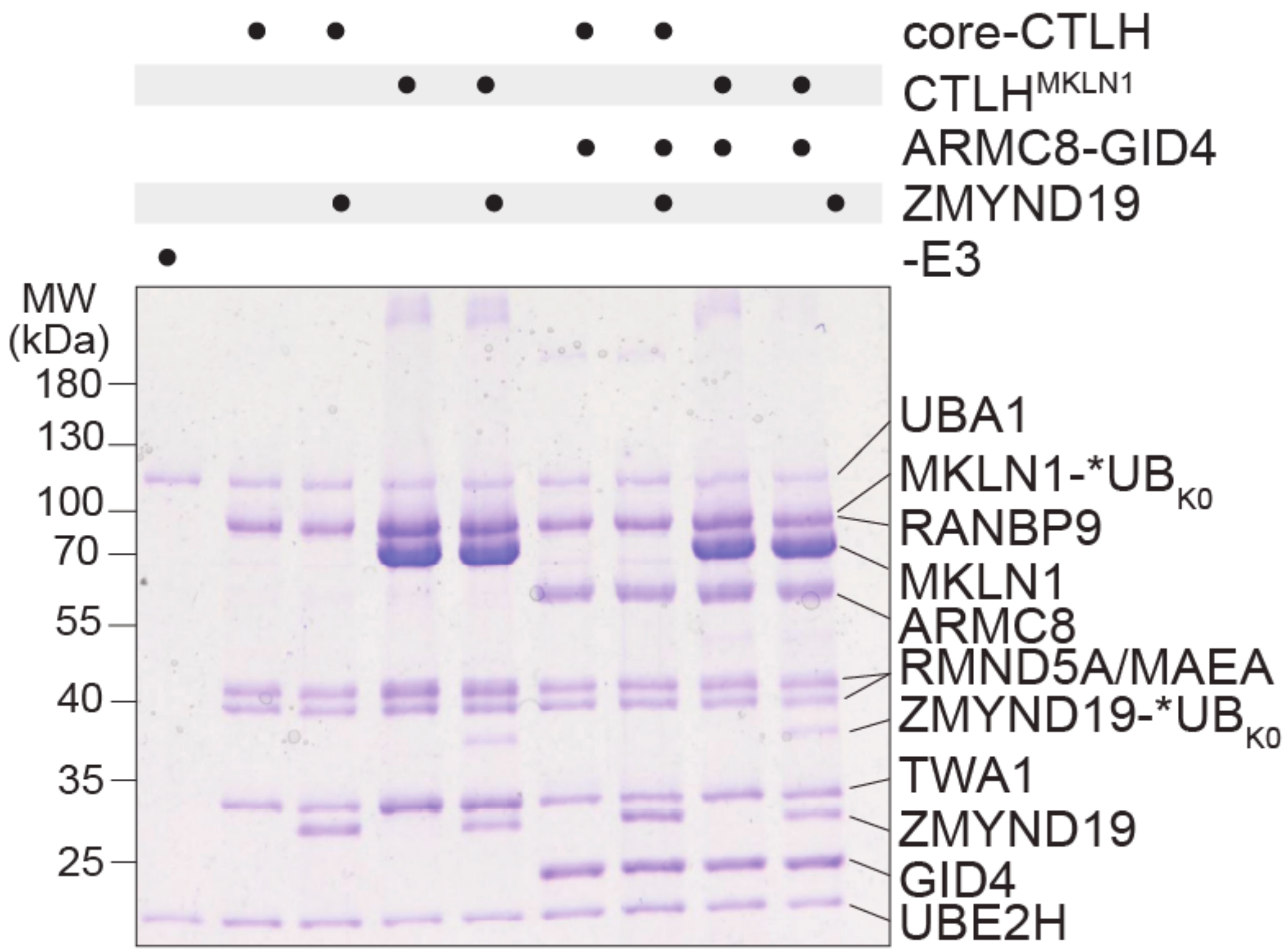

Supplementary Fig. 1 | Representative Coomassie-stained gel of ubiquitylation reaction. Components used in the in vitro ubiquitylation assay shown in Fig.1d, with individual protein bands indicated at their corresponding molecular weights. Source data are provided as a Source Data file.

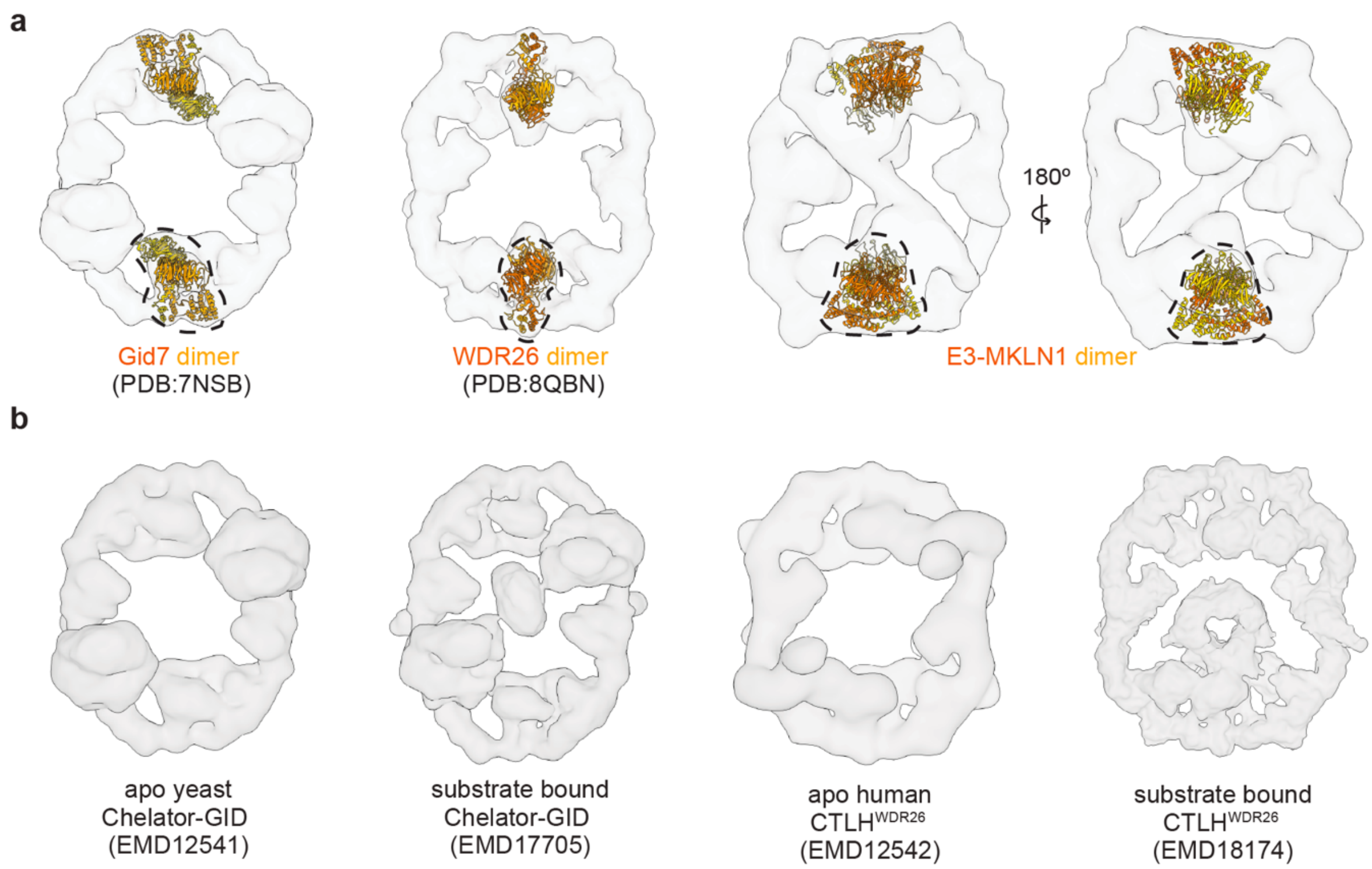

Supplementary Fig. 2 | Survey of giant oval supramolecular GID/CTLH E3 assemblies. a Cryo-EM maps, from left-to-right, shown as transparent surface for substrate-free (apo) yeast Chelator-GID (EMD-12541) and human CTLH^{WDR26}-YPEL5 (EMD-12542), or CTLH^{MKLN1} (this study). Dashed circles indicate the position of the Gid7-like dimer within each assembly. The indicated dimer structures of yeast Gid7 WDR26, and model for E3-MKLN1 (this study) are shown in ribbon diagram fit inside the maps (PDB: 7NSB, 8QBN, and 29UE, respectively). **b** Cryo-EM maps of substrate-free and substrate (Fbp1)-bound yeast Chelator-GID (EMD-12541 and EMD-17705), and substrate-free and substrate (NMNAT1)-bound human CTLH^{WDR26} (EMD-12542 and EMD-18174), illustrating how substrates are engaged inside the hollow central cavity of oval GID/CTLH E3 assemblies, much like Sub-MKLN1 fills the center of CTLH^{MKLN1} (Fig. 3a).

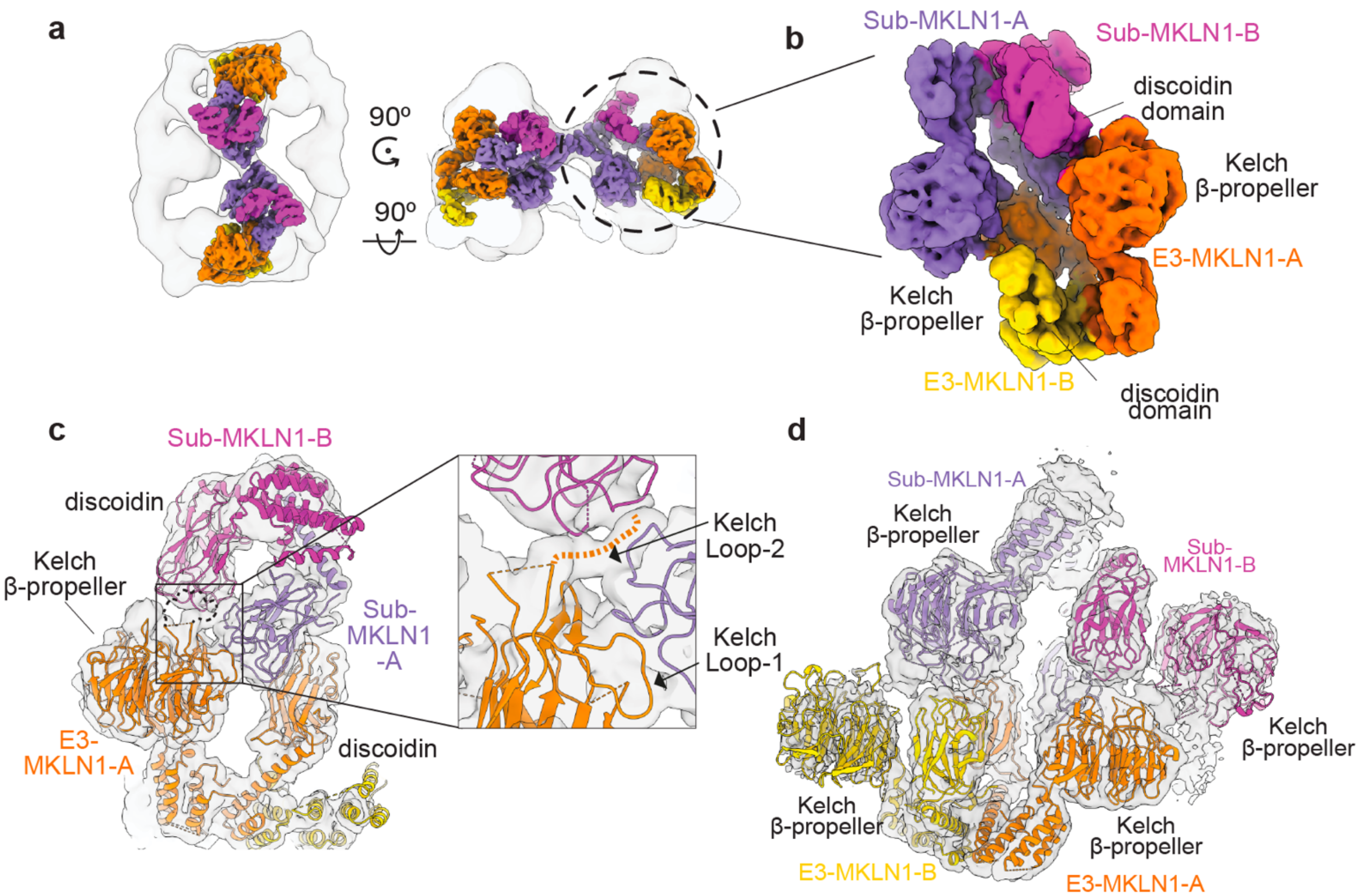

Supplementary Fig. 3 | Cryo-EM analysis of the E3-MKLN1–Sub-MKLN1 tetrameric assembly. a Cryo-EM map of CTLH^{MKLN1}-UBE2H~ubiquitin (transparent) fit with focus-refined map of E3-MKLN1–Sub-MKLN1 tetramer assembly (solid surface colored by MKLN1 protomer). **b** Close-up view of cryo-EM density of tetrameric MKLN1 arrangement illustrating the approximate 2-fold symmetry relating the E3-MKLN1 and Sub-MKLN1 dimers. Within each dimer, the MKLN1 protomers are asymmetrically arranged. **c** Portion of focus-refined map of E3-MKLN1–Sub-MKLN1 tetramer assembly shown as a transparent surface, fitted with model derived from E3-MKLN1–Sub-MKLN1 dimer structure. One protomer E3-MKLN1-A (dark orange) was fit from high-resolution structure of its complex with Sub-MKLN1 (PDB:29UE). The other protomer (E3-MKLN1-B, yellow) was modeled by fitting the domains (discoidin, LisH-CTLH-CRA, Kelch) from the E3-MKLN1-A structure individually into the map. Inset shows E3-MKLN1-A Kelch β-propeller with Loop-1 and density for Loop-2, extending toward Sub-MKLN1 discoidin domains symmetric with the Sub-MKLN1 Kelch β-propeller binding to E3-MKLN1 shown in Fig. 3b. Loop-2 is indicated by dashed lines. **d** Distal Kelch domain arrangements suggest potential for their interactions between E3-MKLN1-B and Sub-MKLN1-A, and between E3-MKLN1-A and Sub-MKLN1-B. E3-MKLN1-B–Sub-MKLN1-B model derived from fitting domains from E3-MKLN1-A–Sub-MKLN1-A structure (PDB 29UE) into the density.

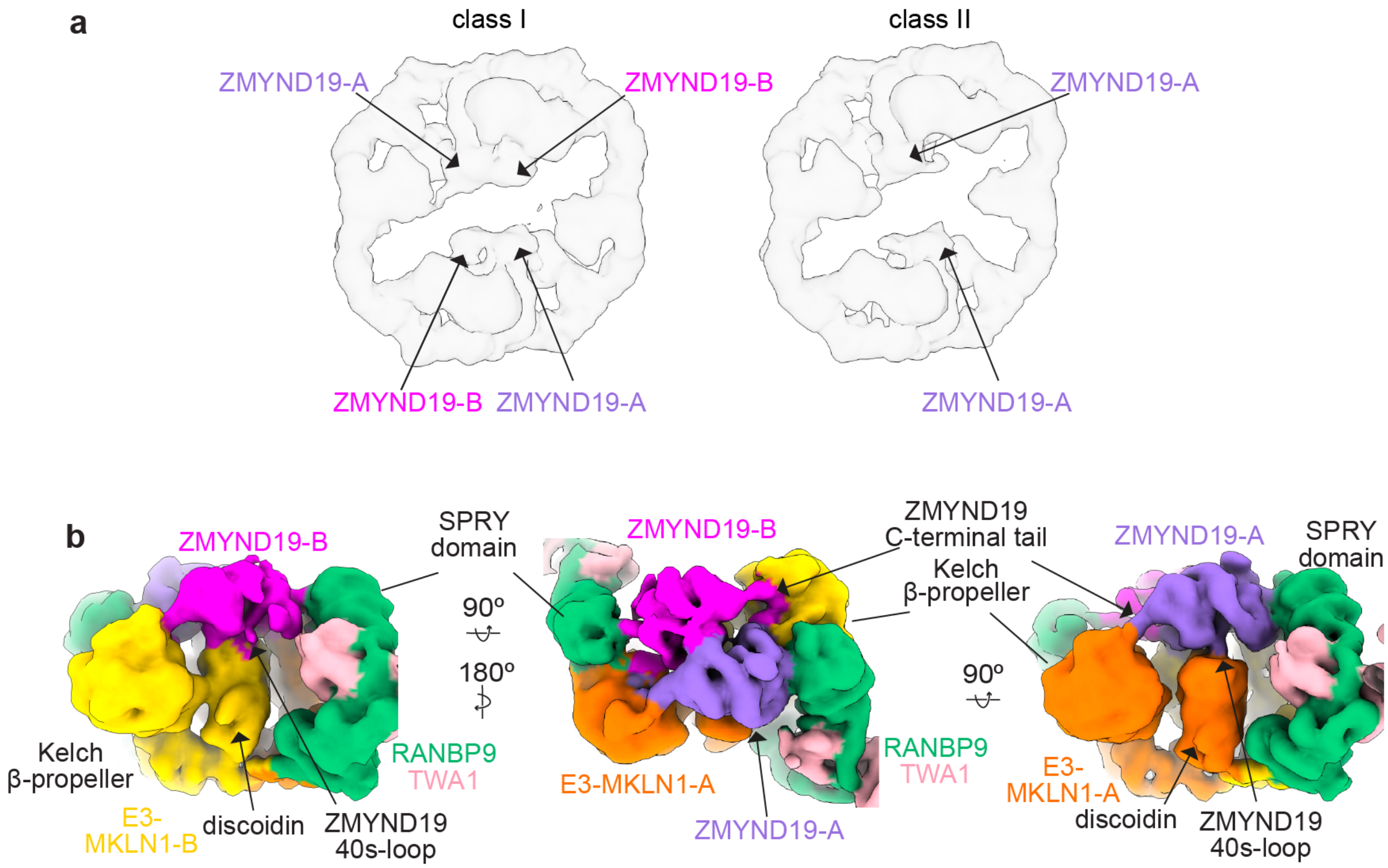

Supplementary Fig. 4 | Varying extents of density for ZMYND19 in different 3D classes of complex with CTLH^{MKLN1} and UBE2H~ubiquitin. a Two 3D classes from cryo-EM data processing of CTLH^{MKLN1}-UBE2H~ubiquitin bound to ZMYND19. Density across the entirely of two ZMYND19 molecules (ZMYND19-A and ZMYND19-B) are visible in Class I, whereas the ZMYND domain from ZMYND19-A is only visible in Class II. **b** Three views of cryo-EM map of the MKLN1-RANBP9-TWA1 subcomplex bound to ZMYND19-A and ZMYND19-B, illustrating the asymmetric arrangement of the two ZMYND19 molecules within the complex. Views highlight the ZMYND19 C-terminal tail of both molecules asymmetrically extending toward the Kelch β-propellers from the two E3-MKLN1 protomers, the ZMYND19-A 40s-loop contacting the E3-MKLN1-A discoidin domain, and the ZMYND19 globular ZMYND domain contacting the RANBP9 SPRY domain.

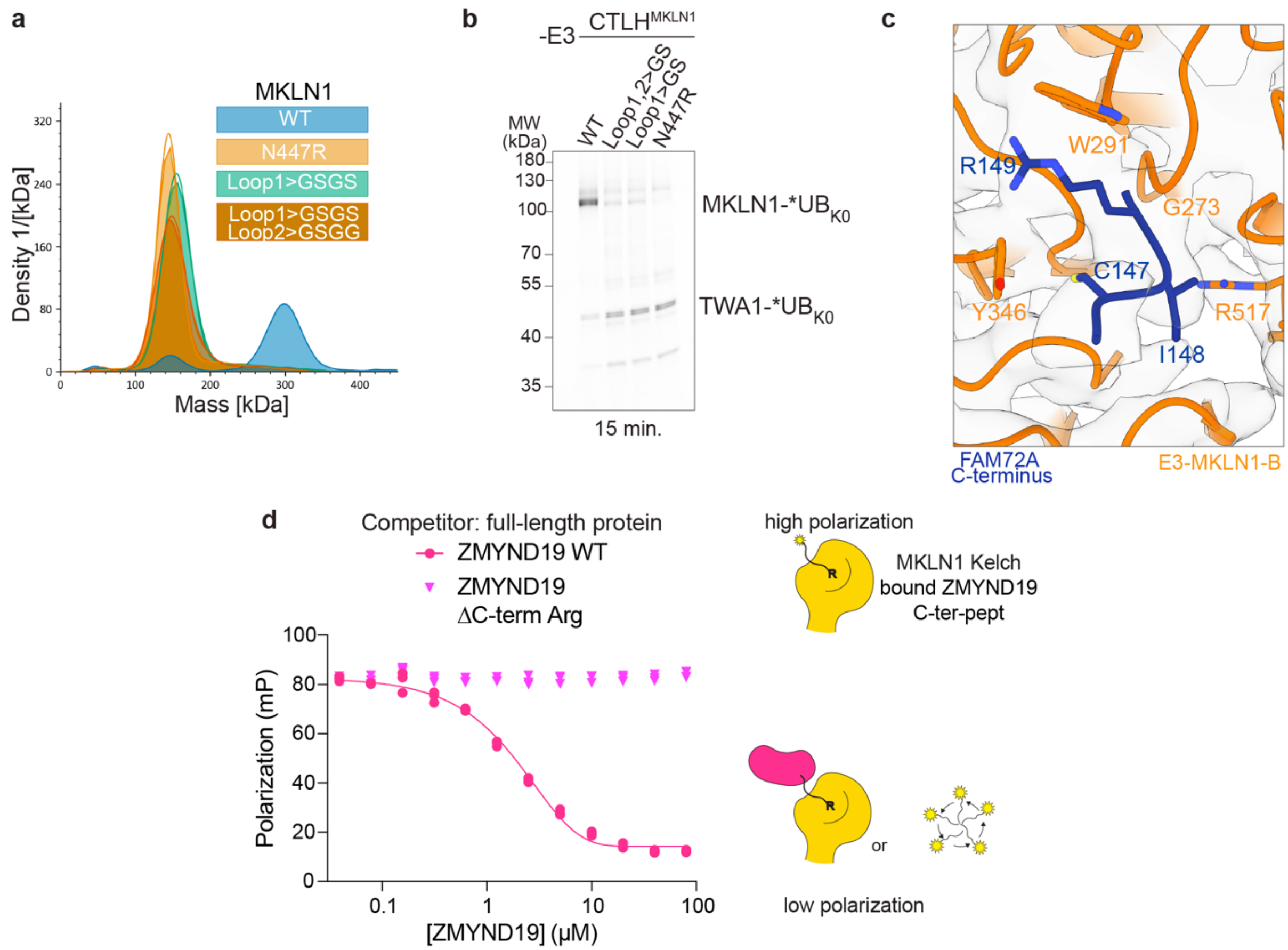

Supplementary Fig. 5 | Biochemical and structural analysis of CTLH^{MKLN1} substrate recognition mediated by MKLN1 Kelch domain. a Mass photometry analysis of wild-type MKLN1, N447R, and Kelch domain loop substitution mutants (Loop1>GSGS; Loop1>GSGS and Loop2>GSGG), showing conversion of isolated MKLN1 from a tetramer to a dimer upon mutation. **b** Assays testing effects of MKLN1 Kelch domain loop substitutions (Loop1>GSGS, Loop1,2>GSGS/GSGG, N447R) on MKLN1 ubiquitylation by CTLH^{MKLN1}. Increased alternative ubiquitylation of scaffolding subunit TWA1, upon disruption of MKLN1 self-recruitment, is indicated (n=3 technical replicates). **c** 3.7 Å resolution focus-refined cryo-EM map showing FAM72A C-terminal residues inserted into E3-MKLN1 Kelch domain channel. **d** Fluorescence polarization assays monitoring N-terminally fluorescein-labeled ZMYND19 C-terminal peptide bound to the MKLN1 Kelch domain upon titration of unlabeled WT ZMYND19 protein or a deletion mutant lacking the C-terminal Arg (ΔC-term Arg) (n=3 technical replicates). Source data are provided as a Source Data file.

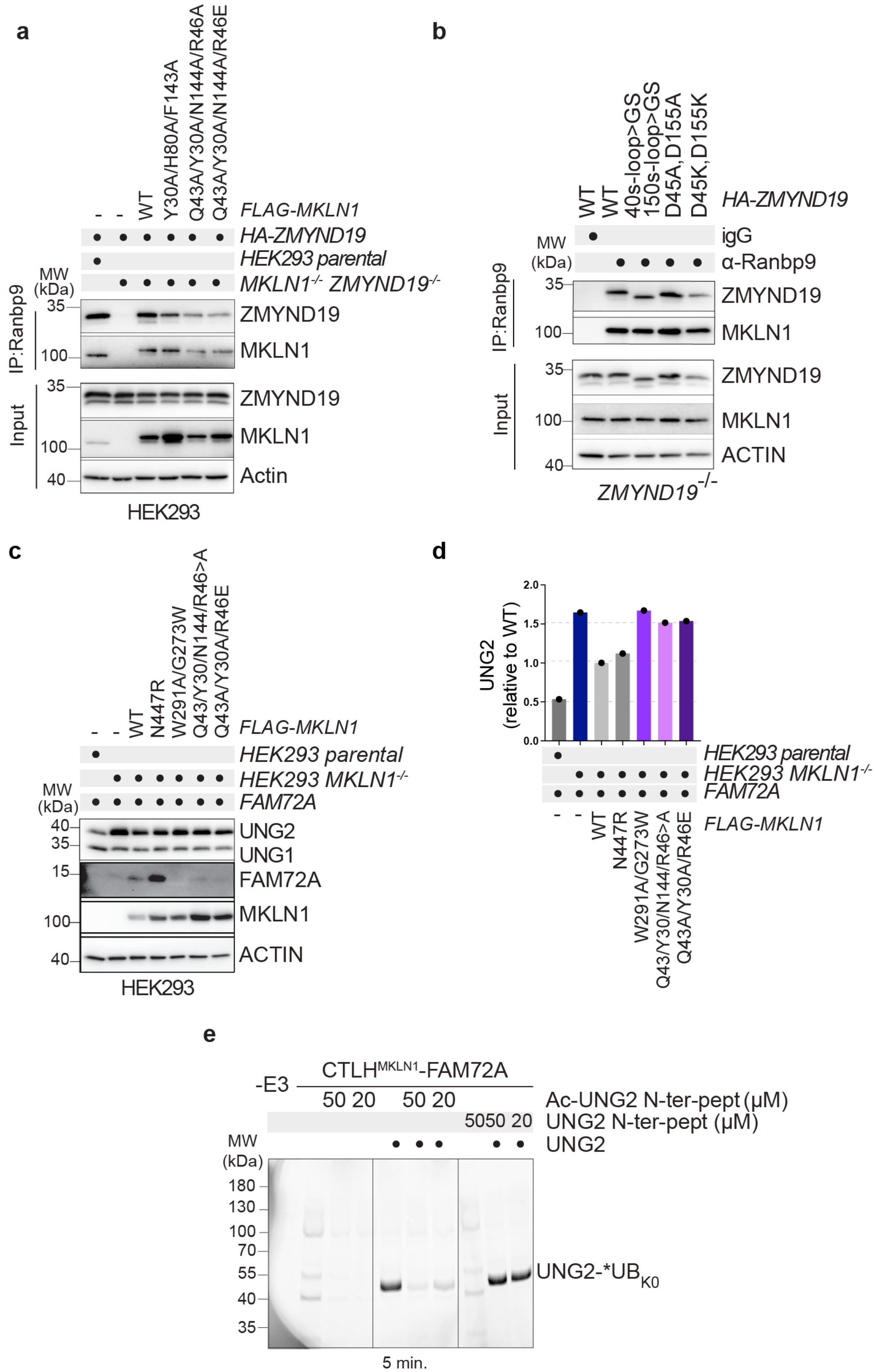
a
FLAG-MKLN1
- - WT Y30A/H80A/F143A Q43A/Y30A/N144A/R46A Q43A/Y30A/N144A/R46E
HA-ZMYND19
HEK293 parental
MKLN1-/- ZMYND19-/-
MW (kDa)
IP:Ranbp9
Input
35
100
40
ZMYND19
MKLN1
Actin
HEK293
b
HA-ZMYND19
WT WT 40s-loop>GS 150s-loop>GS D45A,D155A D45K,D155K
igG
α-Ranbp9
ZMYND19
MKLN1
ACTIN
ZMYND19-/-
c
FLAG-MKLN1
- - WT N447R W291A/G273W Q43/Y30/N144/R46>A Q43A/Y30A/R46E
HEK293 parental
HEK293 MKLN1-/-
FAM72A
UNG2
UNG1
FAM72A
MKLN1
ACTIN
HEK293
d
UNG2 (relative to WT)
2.0
1.5
1.0
0.5
0.0
HEK293 parental
HEK293 MKLN1-/-
FAM72A
FLAG-MKLN1
e
-E3
CTLH MKLN1-FAM72A
50 20 50 20
Ac-UNG2 N-ter-pept (μM)
5050 20
UNG2 N-ter-pept (μM)
UNG2
180
130
100
70
55
40
35
UNG2-*UB K0
5 min.

Supplementary Fig. 6 | Mutational analysis of E3-MKLN1 discoidin domain and corresponding loop mutants of substrate ZMYND19 and substrate-binding adaptor protein FAM72A. a Interactions with CTLH E3, monitored by indicated immunoblots of immunoprecipites (using anti-RANBP9 antibody) from parental HEK293 cells or *MKLN1*$^{-/-}$ *ZMYND19*$^{-/-}$ double knockouts co-transfected with constructs expressing *HA-ZMYND19* and *3xFlag-MKLN1* (WT or discoidin trench mutants). Inputs are shown below. Actin serves as loading control. **b** Interactions with CTLH E3 monitored by indicated immunoblots of immunoprecipites (using anti-RANBP9 antibody or rabbit IgG as a negative control) from *ZMYND19*$^{-/-}$ HEK293 cells transiently expressing *HA-ZMYND19* WT or loop substitution mutants (40s-loop>GS and 150s-loop>GS; D45A/D155A; D45K/D155K). Inputs are shown below. Actin serves as loading control. a-b n=2 biological replicates **c** Immunoblot analysis of UNG2, UNG1, FAM72A, and MKLN1 levels in parental and *MKLN1*-deficient cells co-transfected with constructs expressing untagged *FAM72A* and *3xFlag-MKLN1* (WT or discoidin trench mutants). Actin serves as loading control. **d** Quantification of UNG2 immunoblots from **c.** normalized to actin and then to *MKLN1* null cells transfected with untagged *FAM72A* and WT *3xFlag-MKLN1*; n=1 biological replicate. **e** In vitro assays testing effects of indicated acetylated or non-acetylated UNG2 N-terminal peptide (corresponding to the first 25 amino acids of UNG2) on CTLH^{MKLN1}-FAM72A E3 autoubiquitylation or ubiquitylation of N-terminally acetylated UNG2 protein (CTLH^{MKLN1}-FAM72A E3 complex expressed from a single baculovirus; see Materials); n=2 technical replicates. Source data are provided as a Source Data file.

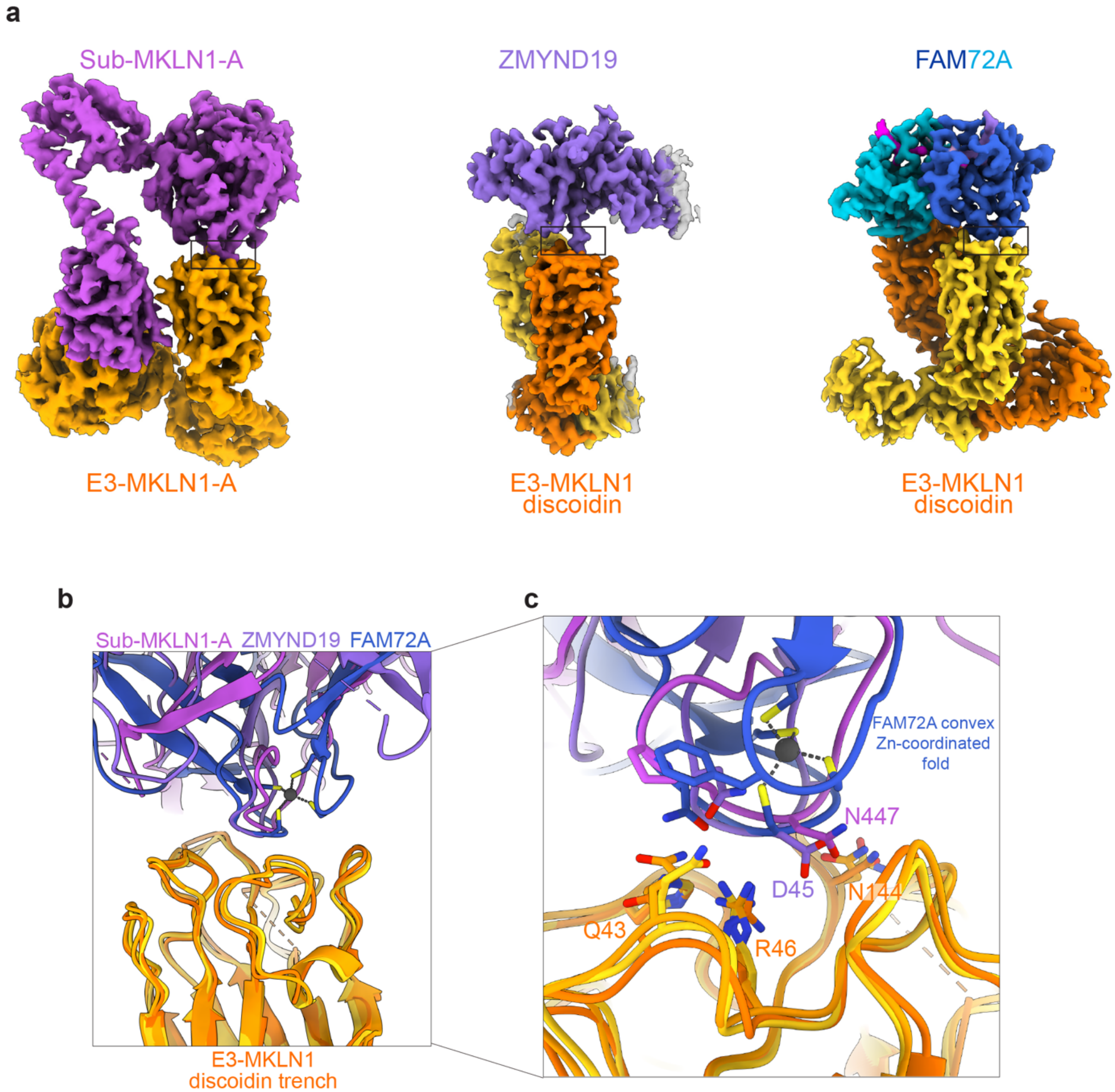

Supplementary Fig. 7 | Structural data showing E3-MKLN1 discoidin domain trench binds Sub-MKLN1, ZMYND19, and FAM72A. a Solid surface representations of focus-refined cryo-EM maps of the E3-MKLN1 discoidin domain(s) bound to Sub-MKLN1-A (left)**,** ZMYND19 (middle), or UNG2-bound FAM72A (right). Boxed regions indicate the discoidin trench interface regions highlighted in panels **b** and **c**. **b** Superposition of the structures showing E3-MKLN1 discoidin domain (orange/yellow) bound to Sub-MKLN1-A (purple), ZMYND19 (light purple), and FAM72A (blue), illustrating their binding to the discoidin domain trench. **c** Close-up view of the superposition in **b**.

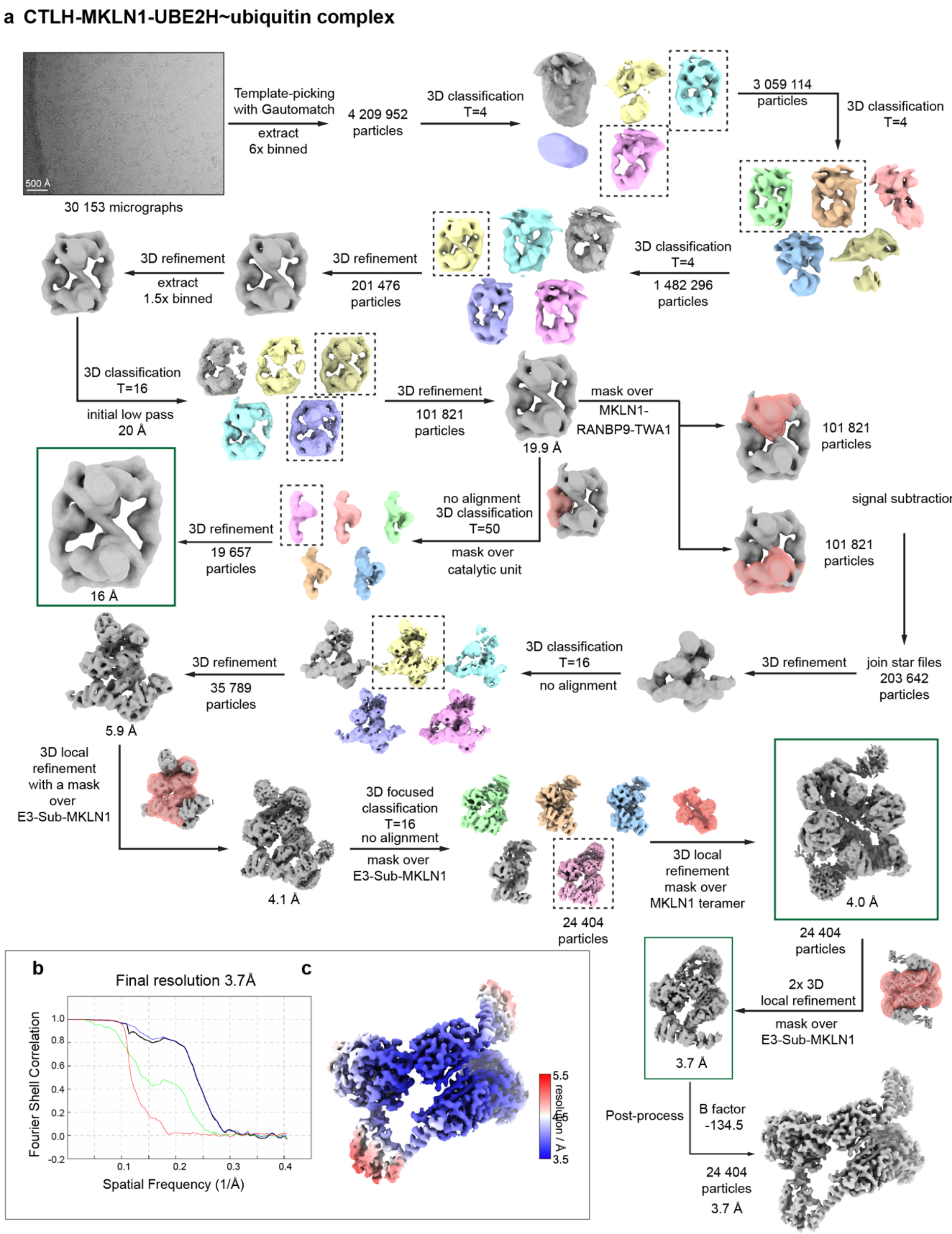

Supplementary Fig. 8 | Cryo-EM data processing scheme for the CTLH^{MKLN1} complex. a Representative micrograph is shown; scalebar, 500 Å. Data were processed in RELION. Classes selected from classification steps are shown in boxes. Green boxes indicate maps used for Fig. 3b and Supplementary Fig. 2 of this study. Masks are shown on the flowchart arrow when used. **b** Gold-standard Fourier shell correlation at 0.143 cutoff. The final reconstruction reached 3.7 Å resolution. **c** Local resolution estimation.

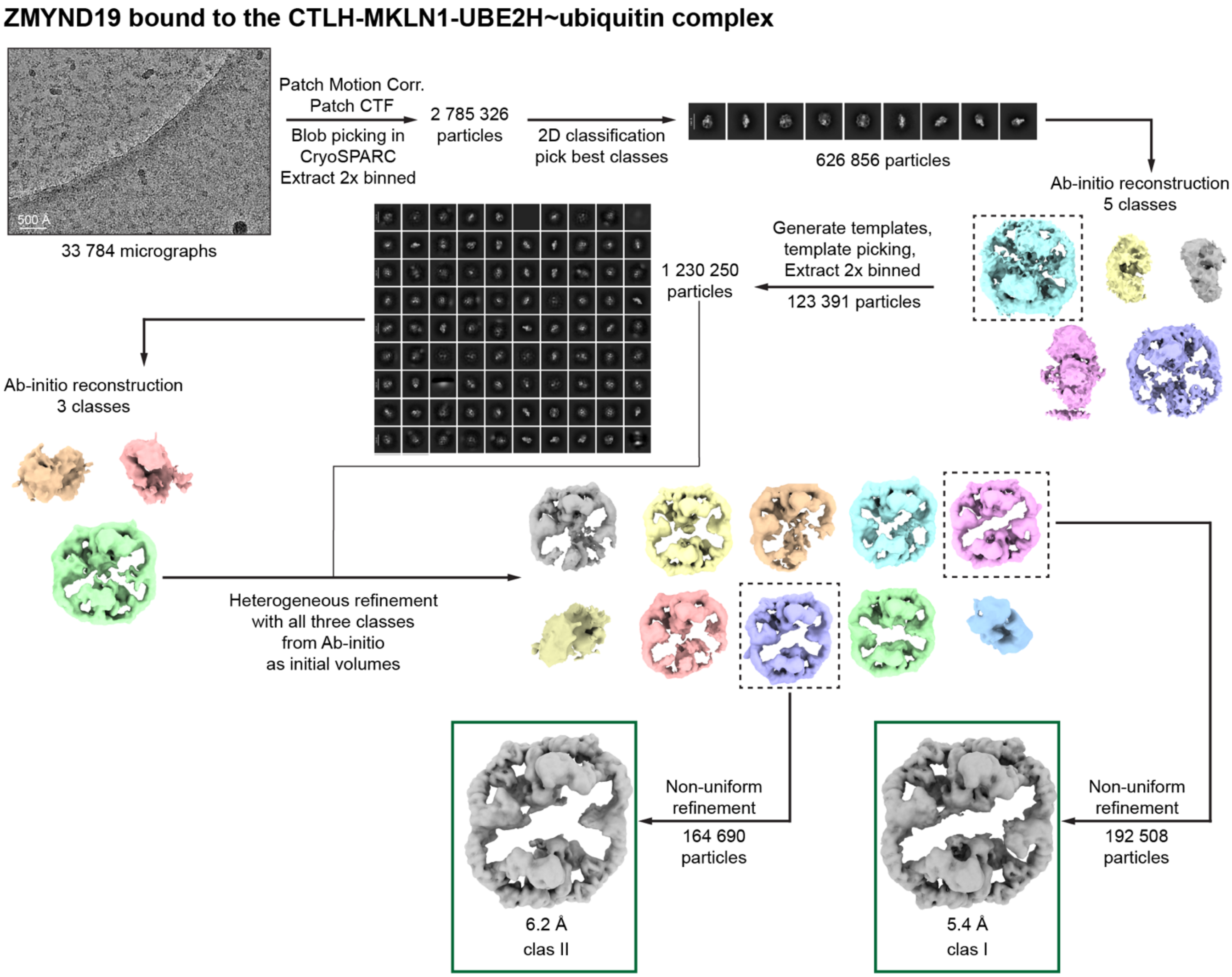

Supplementary Fig. 9 | Cryo-EM data processing scheme for the CTLH^{MKLN1}-ZMYND19 complex. Representative micrograph is shown; scalebar, 500 Å. Data were processed in cryoSPARC. Templates were generated from Ab-initio model. Green boxes indicate maps used for Fig. 2 (class I) and Supplementary Fig. 4a of this study.

ZMYND19 bound to the MKLN1-RANBP9-TWA1 subcomplex

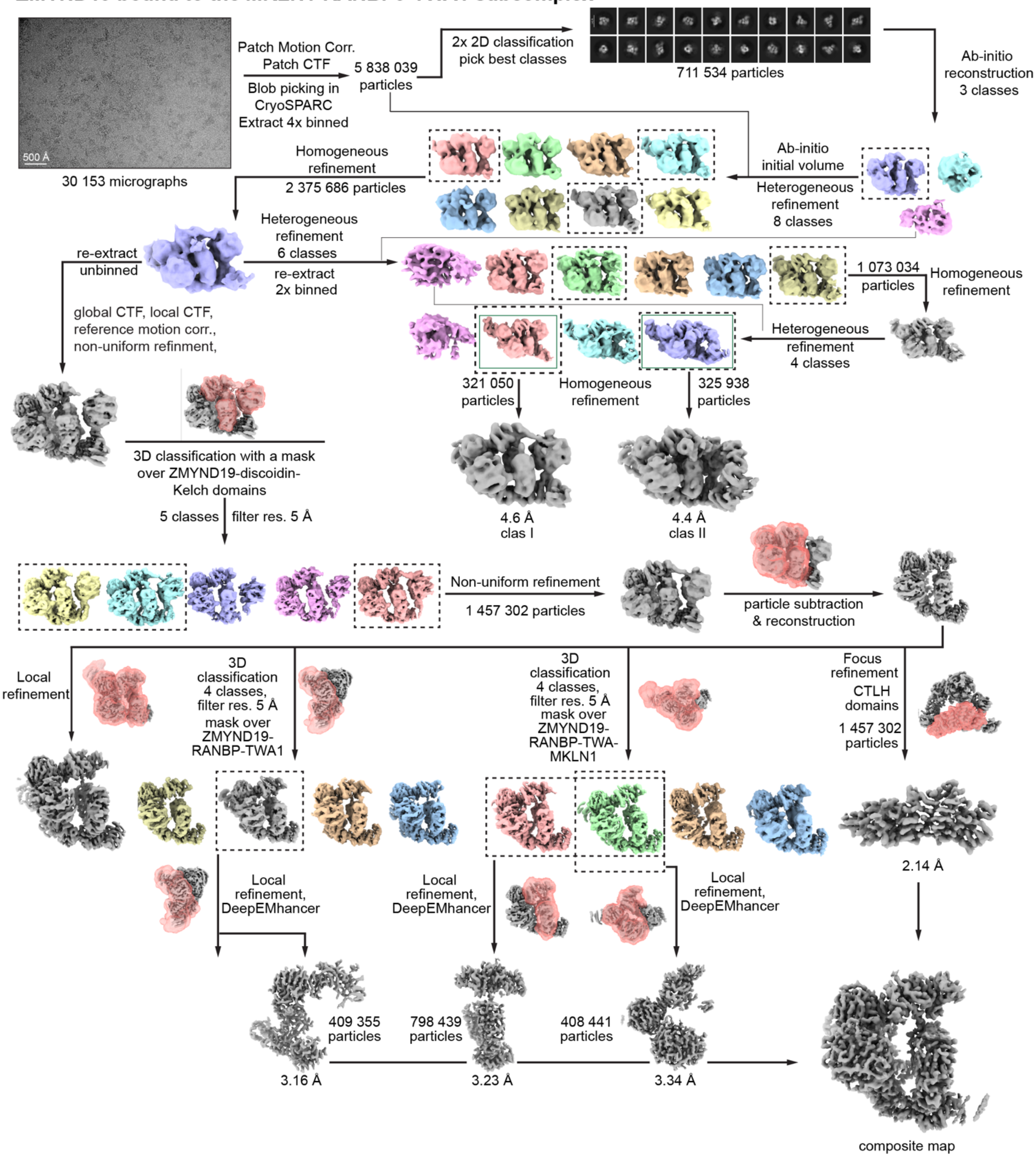

Supplementary Fig. 10 | Cryo-EM data processing scheme for the ZMYND19-MKLN1-RANBP9-TWA1 subcomplex. Representative micrograph is shown; scalebar, 500 Å. Data were processed in cryoSPARC. Ab-initio model was directly used for Heterogeneous refinement. Green boxes indicate maps used for Fig. 4a (class I) and Supplementary Fig. 3b of this study. High-resolution maps were achieved through particle subtraction and local-refinement with masks. They were used to generate the composite map. Maps used for composite map were sharpened with DeepEMhancer.

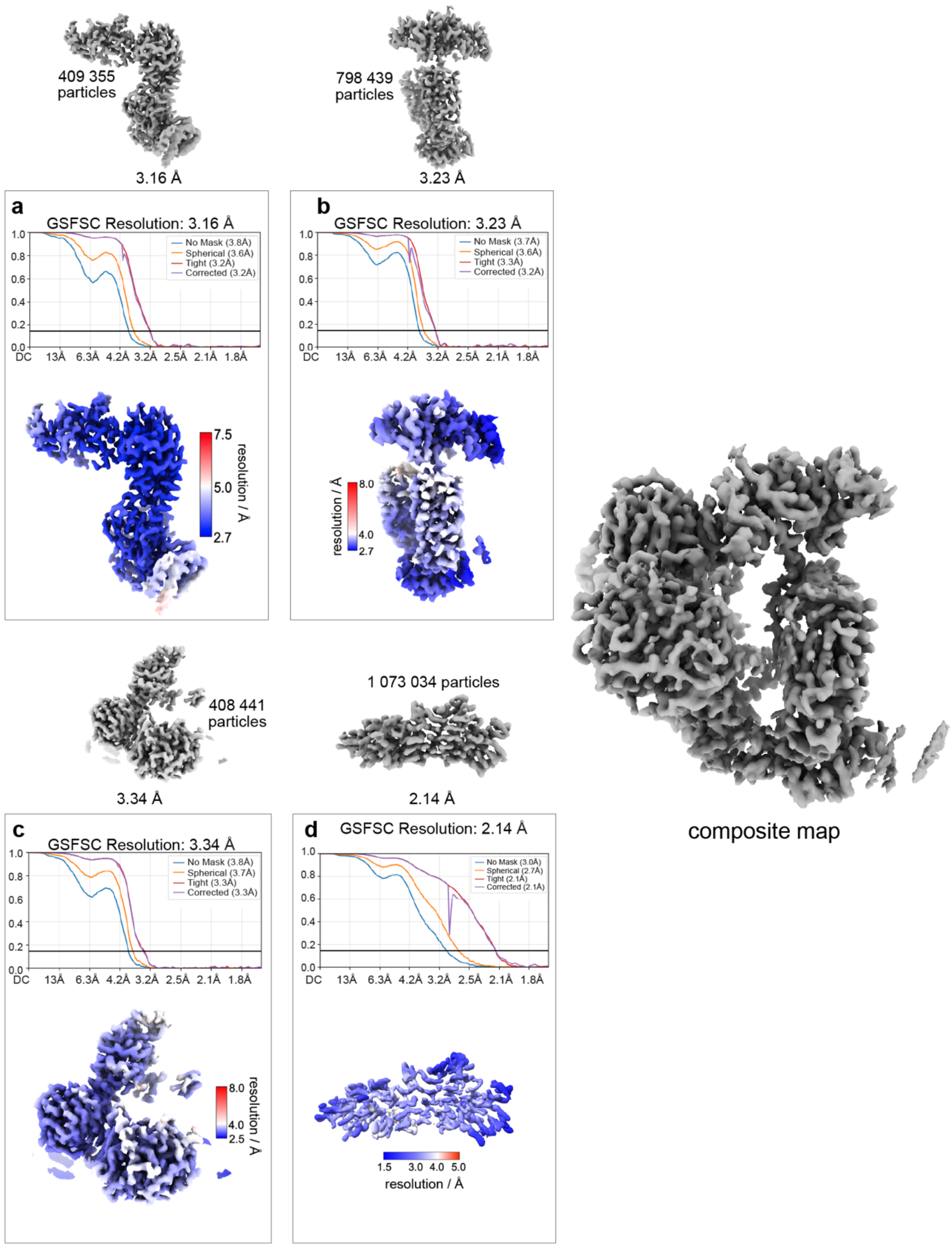

Supplementary Fig. 11 | a-d Final reconstructions with particle counts and resolutions displayed on each map used to generate a composite map. Gold-standard Fourier shell correlation (GSFSC) at 0.143 cutoff and local resolution estimation are shown for each sharpened map. The composite map is shown on the right.

FAM72A-UNG2 bound to CTLH-MKLN1-UBE2H~ubiquitin complex

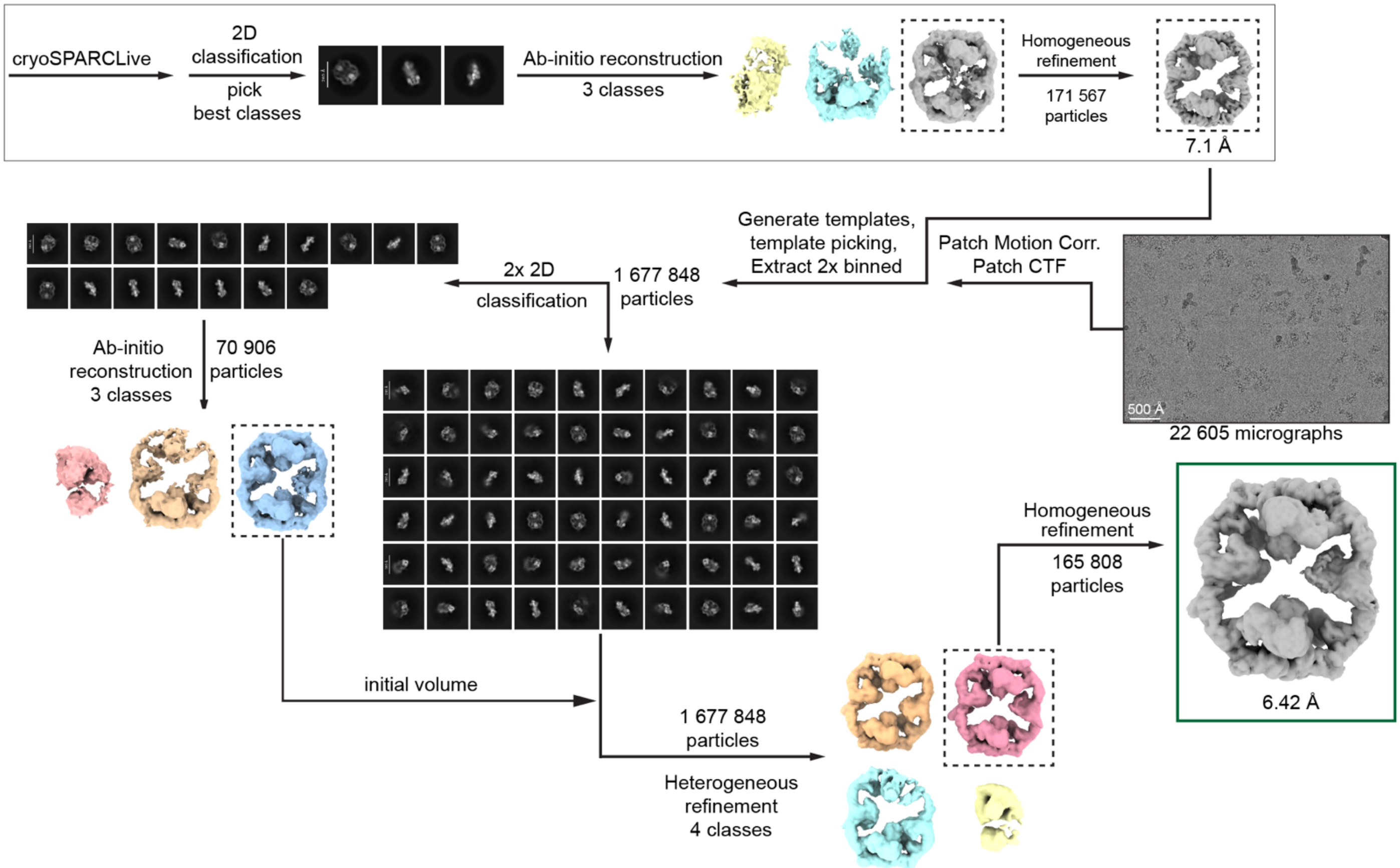

Supplementary Fig. 12 | Cryo-EM data processing scheme for the CTLH^{MKLN1}-FAM72A UNG2 subcomplex. Representative micrograph is shown; scalebar, 500 Å. Data were processed in cryoSPARC. Templates were generated from cryoSPARC Live processing Ab-initio model. Green box indicates map used for Fig. 2 of this study.

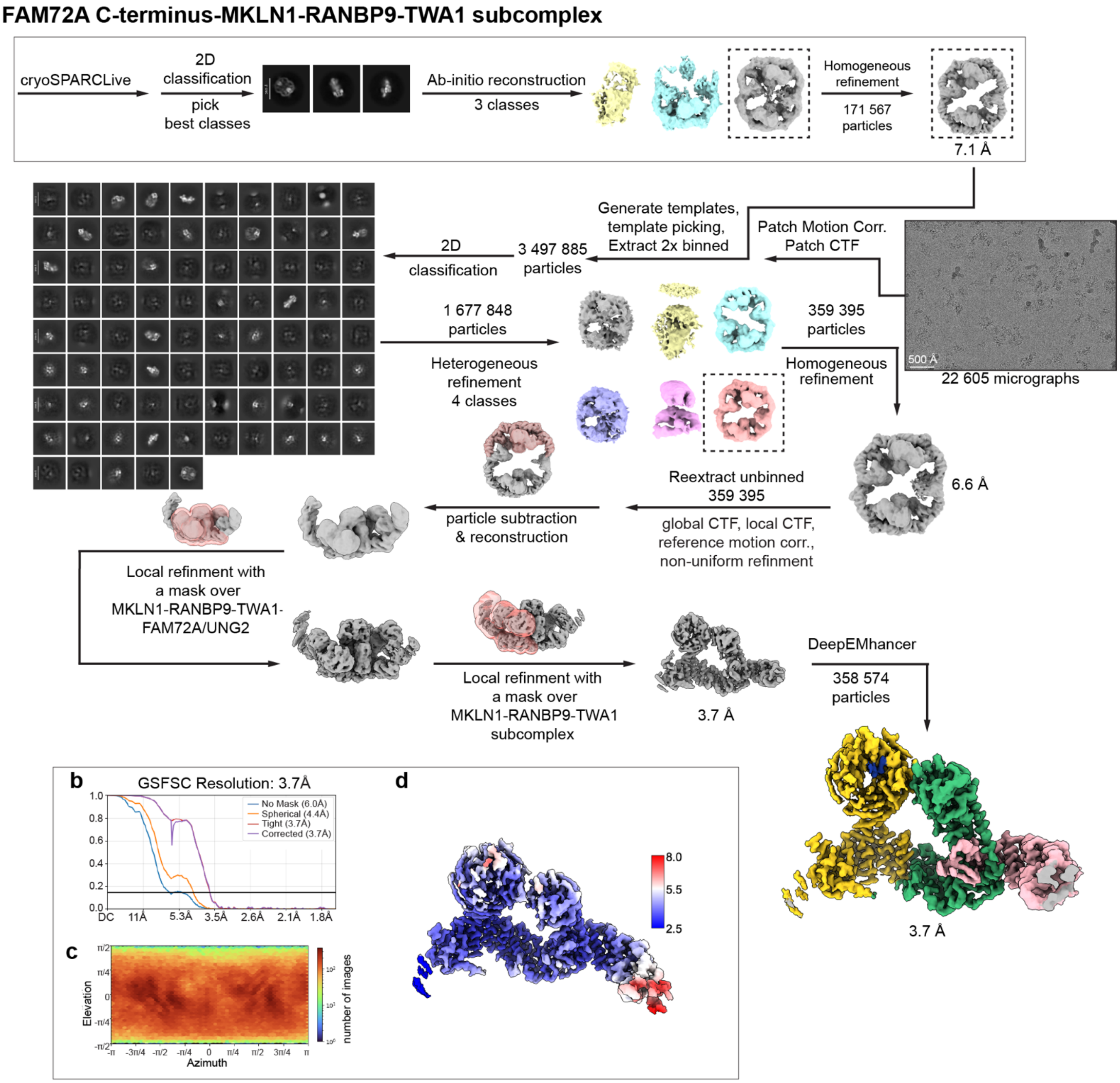

Supplementary Fig. 13 | Cryo-EM data processing scheme for the FAM72A C-terminus-MKLN1-RANBP9-TWA1 subcomplex. Representative micrograph is shown; scalebar, 500 Å. Data were processed in cryoSPARC. Templates were generated from cryoSPARC Live processing Ab-initio model. **b** The final reconstruction reached 3.7 Å resolution by the gold-standard Fourier shell correlation at 0.143 cutoff and was sharpened with DeepEMhancer. Gold-standard Fourier shell correlation curve. **c** Orientation distribution plot. **d** Local resolution estimation.

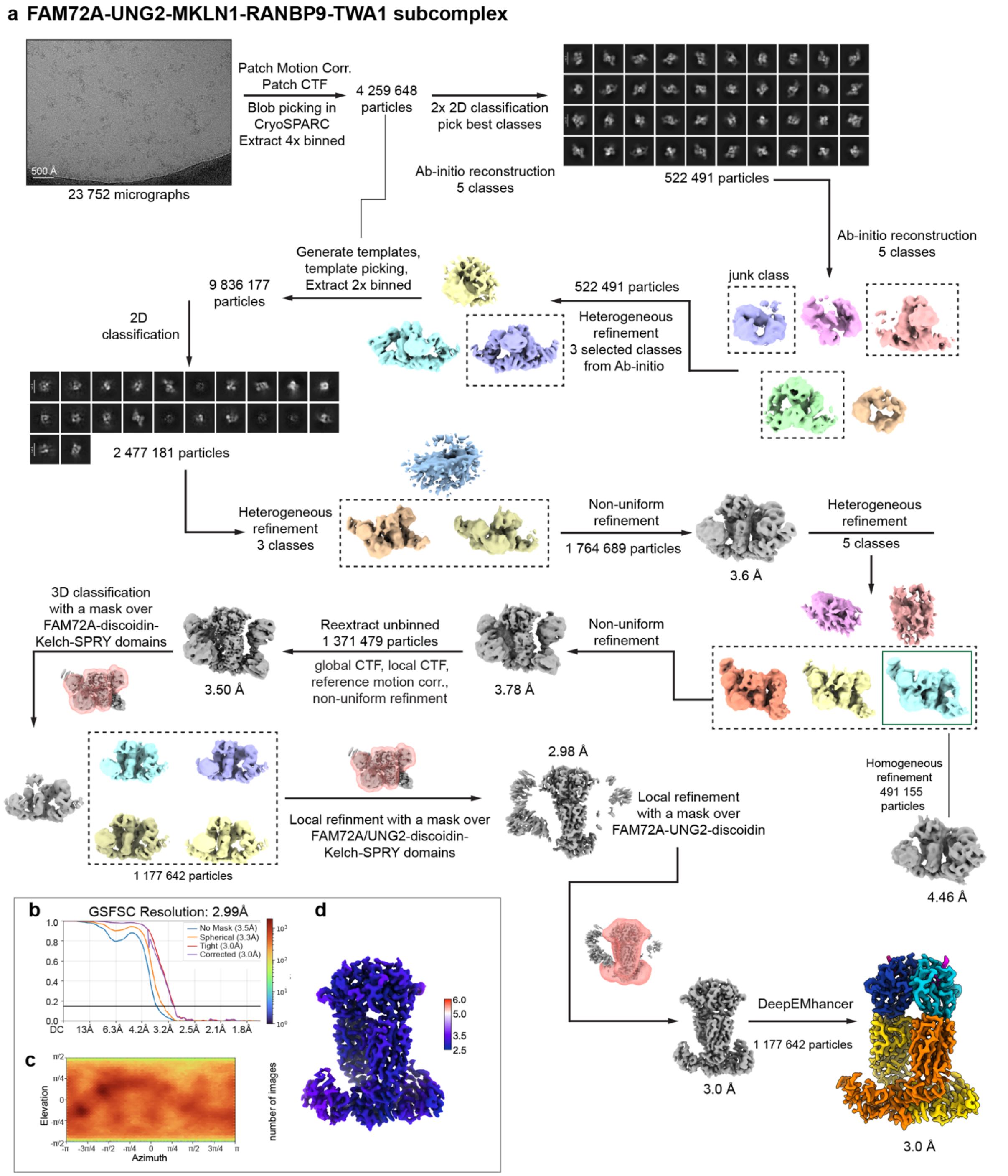

Supplementary Fig. 14 | Cryo-EM data processing scheme for the FAM72A-UNG2-MKLN1-RANBP9-TWA1 subcomplex. a Representative micrograph is shown; scalebar, 500 Å. Data were processed in cryoSPARC. Classes selected from classification steps are shown in boxes. Heterogeneous refinements contained one junk class. Green box indicates map used for Fig. 5a of this study. **b** The final reconstruction reached 3.0 Å resolution by the gold-standard Fourier shell correlation at 0.143 cutoff and was sharpened with DeepEMhancer. Gold-standard Fourier shell correlation curve. **c** Orientation distribution plot. **d** Local resolution estimation.

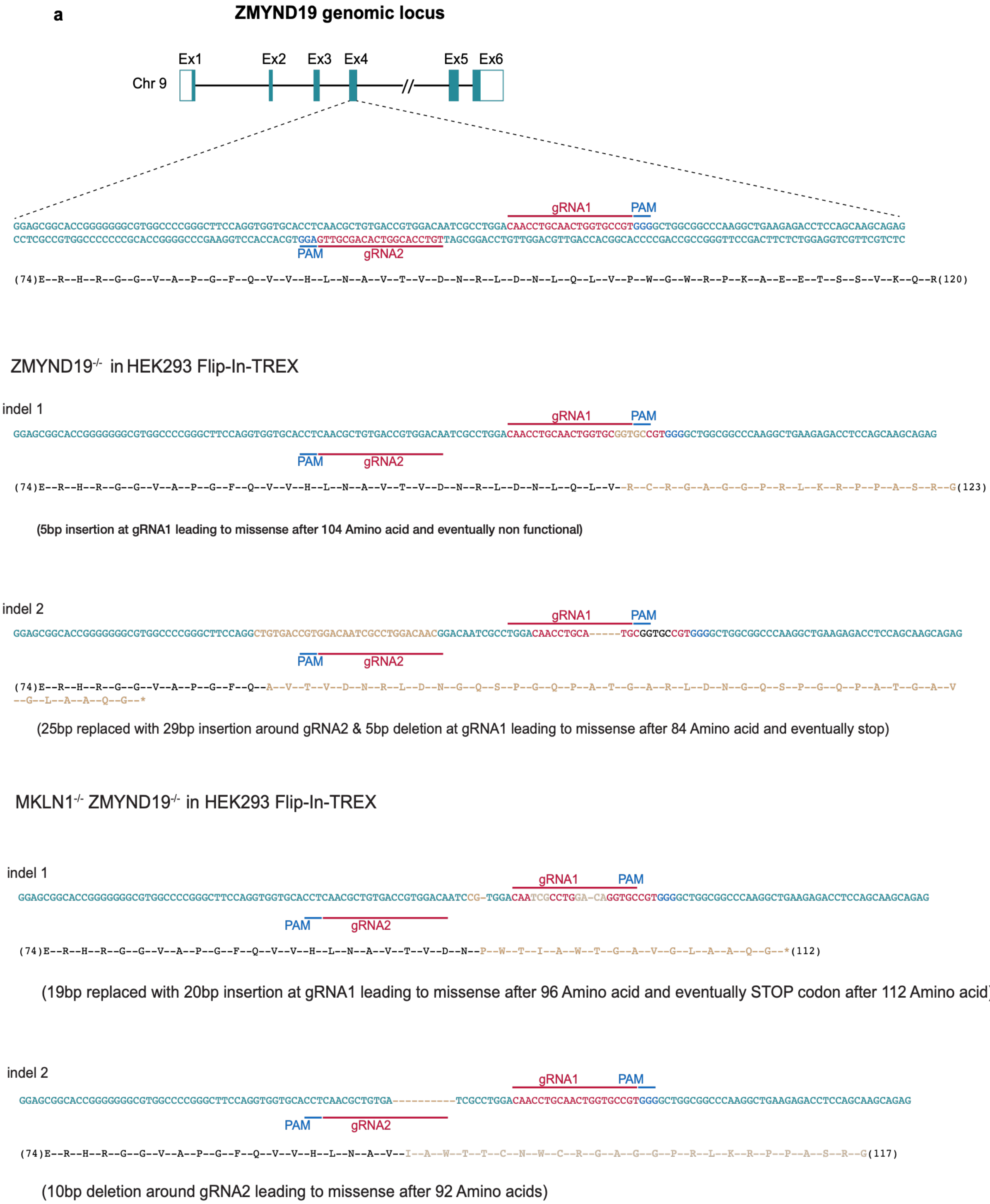
a
ZMYND19 genomic locus
Ex1 Ex2 Ex3 Ex4 Ex5 Ex6
Chr 9
gRNA1 PAM
PAM gRNA2
ZMYND19-/- in HEK293 Flip-In-TREX
indel 1
(5bp insertion at gRNA1 leading to missense after 104 Amino acid and eventually non functional)
indel 2
(25bp replaced with 29bp insertion around gRNA2 & 5bp deletion at gRNA1 leading to missense after 84 Amino acid and eventually stop)
MKLN1-/- ZMYND19-/- in HEK293 Flip-In-TREX
indel 1
(19bp replaced with 20bp insertion at gRNA1 leading to missense after 96 Amino acid and eventually STOP codon after 112 Amino acid)
indel 2
(10bp deletion around gRNA2 leading to missense after 92 Amino acids)

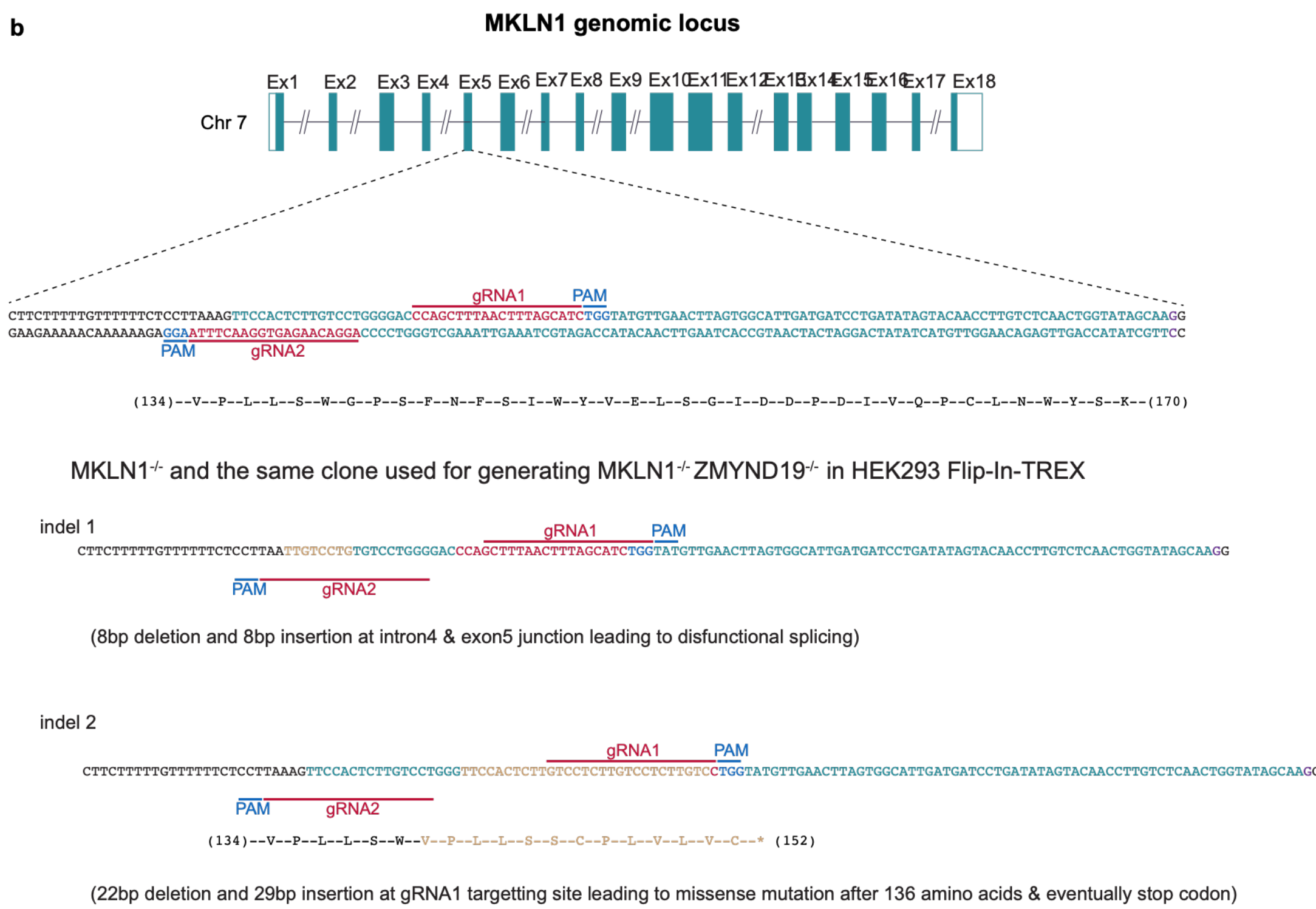

Supplementary Fig. 15 | CRISPR Cas9 (nickase) mediated knockout of *ZMYND19*, *MKLN1* and double knockout of *MKLN1* and *ZMYND19*. a Schematic representation of genomic locus of *ZMYND19* showing exons structure in genome, location of target guide RNAs (gRNAs) pairs (in red), and PAM sequence (in blue). Insertions/deletions of each knockout clones in Flip-In-TREX HEK293 cells (in orange) and its potential missense effects on ZMYND19 protein expression (in orange). **b** Schematic representation of genomic locus of *MKLN1* showing exons structure in genome, location of target guide RNAs (gRNAs) pairs (in red), and PAM sequence (in blue) analogous to **a**.

Supplementary Table 1. | Data collection, refinement and validation statistics.

	Cryo-EM map of substrate-MKLN1 bound CTLH-MKLN1 E3 ligase assembly (EMD-57378)	Cryo-EM structure of tetrameric MKLN1 within CTLH complex (EMD-57376)	Focus refined structure of CTLH E3-MKLN1 and substrate-MKLN1 dimer (EMD-57377) (PDB-29UE)
**Data collection and processing**			
Magnification	105,000	105,000	105,000
Voltage (kV)	300	300	300
Electron exposure (e–/Å^2)	62.6	62.6	62.6
Defocus range (μm)	-0.5 to -2.0	-0.5 to -2.0	-0.5 to -2.0
Pixel size (Å)	1.2333	1.2333	1.2333
Symmetry imposed	C1	C1	C1
Initial particle images (no.)	4 209 952	4 209 952	4 209 952
Final particle images (no.)	19 657	24 404	24 404
Map resolution (Å)	16	4.0	3.7
FSC threshold	0.143	0.143	0.143
Map resolution range (Å)			3.1-3.8
**Refinement**			
Initial model used (PDB code)			Alphafold3
Model resolution (Å)			3.7
FSC threshold			0.143
Model resolution range (Å)			
Map sharpening *B* factor (Å^2)			-134.5
Model composition			
Non-hydrogen atoms			8142
Protein residues			1117
Ligands			
B factors (Å^2)			
Protein			40.15
Ligand			
R.m.s. deviations			
Bond lengths (Å)			0.003
Bond angles (°)			0.5
Validation			
MolProbity score			1.44
Clashscore			3.30
Poor rotamers (%)			0.00
Ramachandran plot			
Favored (%)			95.42
Allowed (%)			4.58
Disallowed (%)			0.00

Supplementary Table 1. | Data collection, refinement and validation statistics.

	Cryo-EM map of ZMYND19-bound CTLH-MKLN1 E3 ligase – class I (EMD-57380)	Cryo-EM map of ZMYND19-bound CTLH-MKLN1 E3 ligase - class II (EMD-57379)	Cryo-EM map of ZMYND19 bound to RANBP9-TWA1-MKLN1 subcomplex - class I (EMD-57381)	Cryo-EM map of ZMYND19 bound to RANBP9-TWA1-MKLN1 subcomplex - class II (EMD-57382)	Cryo-EM structure of ZMYND19 bound to MKLN1-RANBP9-TWA1 CTLH subcomplex (EMD-56913)
**Data collection and processing**					
Magnification	105,000	105,000	105,000	105,000	105,000
Voltage (kV)					
Electron exposure (e–/Å^2)	45.6	45.6	59	59	59
Defocus range (μm)	-0.5 to -2.0	-0.5 to -2.0	-0.5 to -2.0	-0.5 to -2.0	-0.5 to -2.0
Pixel size (Å)	1.6444	1.6444	1.6444	1.6444	1.6444
Symmetry imposed	C1	C1	C1	C1	C1
Initial particle images (no.)	2 785 326	2 785 326	5 838 039	5 838 039	5 838 039
Final particle images (no.)	192 508	164 690	321 050	325 938	1 457 302
Map resolution (Å)	5.33	6.13	4.6	4.4	3.3
FSC threshold	0.143	0.143	0.143	0.143	0.143
Map resolution range (Å)	4.4-6.6	4.6-7.1	4.1-6.3	4.0-4.7	3.2-3.6
**Refinement**					
Initial model used (PDB code)					
Model resolution (Å)					
FSC threshold					
Model resolution range (Å)					
Map sharpening *B* factor (Å^2)					
Model composition					
Non-hydrogen atoms					
Protein residues					
Ligands					
B factors (Å^2)					
Protein					
Ligand					
R.m.s. deviations					
Bond lengths (Å)					
Bond angles (°)					
Validation					
MolProbity score					
Clashscore					
Poor rotamers (%)					
Ramachandran plot					
Favored (%)					
Allowed (%)					
Disallowed (%)					

Supplementary Table 1. | Data collection, refinement and validation statistics.

	Focus refined map of ZMYND19 bound to discoidin domain of substrate receptor MKLN1 (EMD-56909)	Focus refined map of ZMYND19 bound to RANBP9-TWA1 (EMD-56910)	Focus refined map of CTLH domains of MKLN1 and RANBP9 (EMD-56911)	Focus refined map of ZMYND19 bound to SPRY domain of RANBP9 and Kelch propeller of MKLN1 (EMD-56912)	Structure of MKLN1-RANBP9-TWA1 CTLH subcomplex bound to ZMYND19 substrate (EMD-57385) (PDB-29UG)
**Data collection and processing**					
Magnification	105,000	105,000	105,000	105,000	105,000
Voltage (kV)					
Electron exposure (e–/Å^2)	59	59	59	59	59
Defocus range (μm)	-0.5 to -2.0	-0.5 to -2.0	-0.5 to -2.0	-0.5 to -2.0	-0.5 to -2.0
Pixel size (Å)	0.8222	0.8222	0.8222	0.8222	0.8222
Symmetry imposed	C1	C1	C1	C1	C1
Initial particle images (no.)	5 838 039	5 838 039	5 838 039	5 838 039	5 838 039
Final particle images (no.)	798 439	409 355	1 457 302	408 441	1 457 302
Map resolution (Å)	3.23	3.2	2.14	3.34	3.4
FSC threshold	0.143	0.143	0.143	0.143	0.143
Map resolution range (Å)	3.1-3.7	3.1-3.5	2.2-2.7	3.1-3.8	2.2-3.8
**Refinement**					
Initial model used (PDB code)					Alphafold3,7NSC
Model resolution (Å)					3.4
FSC threshold					0.143
Model resolution range (Å)					2.2-3.8
Map sharpening *B* factor (Å^2)					
Model composition					
Non-hydrogen atoms					11956
Protein residues					1618
Ligands					2 Zn^{2+}
B factors (Å^2)					
Protein					31.85
Ligand					35.42
R.m.s. deviations					
Bond lengths (Å)					0.004
Bond angles (°)					0.576
Validation					
MolProbity score					1.80
Clashscore					6.84
Poor rotamers (%)					0.1%
Ramachandran plot					
Favored (%)					93.52
Allowed (%)					6.48
Disallowed (%)					0.00

Supplementary Table 1. | Data collection, refinement and validation statistics.

	Cryo-EM map of FAM72A-bound CTLH-MKLN1 E3 ligase (EMD-57383)	Cryo-EM map of FAM72A-UNG2 bound to RANBP9-TWA1-MKLN1 subcomplex (EMD-57384)	FAM72A-UNG2 bound to the discoidin domain of substrate receptor MKLN1 (EMD-57387) (PDB-29UI)	MKLN1-RANBP9-TWA1 CTLH subcomplex bound to FAM72A C-terminus (EMD-58652) (PDB-31TM)
**Data collection and processing**				
Magnification	105,000	105,000	105,000	105,000
Voltage (kV)	300	300	300	300
Electron exposure (e–/Å^2)	51.7	53.0	53.0	53.0
Defocus range (μm)	-0.5 to -2.0	-0.5 to -2.0	-0.5 to -2.0	-0.5 to -2.0
Pixel size (Å)	0.8222	0.8222	0.8222	0.8222
Symmetry imposed	C1	C1	C1	C1
Initial particle images (no.)	1 677 848	9 836 177	9 836 177	3 497 885
Final particle images (no.)	165 808	491 155	1 177 642	358 574
Map resolution (Å)	6.5	4.5	3.0	3.7
FSC threshold	0.143	0.143	0.143	0.143
Map resolution range (Å)	5.7-7.1	4.1-5.0	2.96-3.3	3.5-4.11
**Refinement**				
Initial model used (PDB code)			Alphafold3	Alphafold3
Model resolution (Å)			3.0	3.7
FSC threshold			0.143	0.143
Model resolution range (Å)				
Map sharpening *B* factor (Å^2)				
Model composition				
Non-hydrogen atoms			6708	7511
Protein residues			834	1038
Ligands			4 Zn^{2+}; 2 ACE	
B factors (Å^2)				
Protein			49.84	101.34
Ligand			55.35	
R.m.s. deviations				
Bond lengths (Å)			0.003	0.002
Bond angles (°)			0.506	0.433
Validation				
MolProbity score			1.35	1.32
Clashscore			6.27	4.24
Poor rotamers (%)			0.00	0.00
Ramachandran plot				
Favored (%)			98.28	97.42
Allowed (%)			1.72	2.58
Disallowed (%)			0.00	0.00
