# Supplementary material for: Multimodal substrate recruitment enables CTLHMKLN1 E3 ligase to target acetylated N-, C-, and internal degrons
Source: Nat Commun. 2026 Oct 17;17:10877. doi: 10.1038/s41467-026-78311-4 (PMC13676621; doi:10.1038/s41467-026-78311-4)
Supplement: Supplementary file 2 [file 41467_2026_78311_MOESM3_ESM.pdf]

nature portfolio

Peer Review File

Multimodal substrate recruitment enables CTLH^{MKLN1} E3 ligase to target N-, C-, and internal degrons

Corresponding Author: Professor Brenda Schulman

This file contains all reviewer reports in order by version, followed by all author rebuttals in order by version.

Version 0:

Reviewer comments:

Reviewer #1

(Remarks to the Author)
The manuscript by Sepic et al. presents an extensive structural and biochemical analysis of substrate recognition by the CTLHMKLN1 E3 ligase complex. Using cryo-EM, in vitro ubiquitylation assays, mutational analysis, and cellular experiments, the authors propose that MKLN1 employs combinatorial use of discoidin and Kelch β-propeller surfaces to recruit distinct substrate classes, including Arg/C-degrons, internal loop motifs, and adaptor-mediated Ac/N-degrons.
The study is technically strong and represents a significant advance for the field. The structural work is impressive, and the biochemical reconstitution is rigorous. However, several mechanistic conclusions currently extend beyond the available evidence, and additional clarification and moderation of claims are required before publication in Nature Communications.
Major points
1. The manuscript repeatedly refers to several motifs as “degrons,” although sufficiency for degradation has not been demonstrated. The authors should either provide reporter-based degron assays or moderate the terminology throughout.
2. The proposed competition between MKLN1 self-recruitment and recruitment of other substrates is attractive but remains indirect. This model could be tested more directly by quantitative competition assays in which ZMYND19 or FAM72A–UNG2 are titrated against MKLN1 autoubiquitylation, or by reciprocal experiments testing whether overexpression of MKLN1 suppresses ZMYND19/UNG2 ubiquitylation and degradation.
3. Cellular validation is comparatively limited relative to the structural claims. Additional degradation or protein half-life analyses would strengthen the physiological relevance of the proposed interfaces.
Minor points
1. Terminology should be standardized throughout (e.g., CTLH^MKLN1 versus CTLHMKLN1).
2. P18, l. 567, 578, 579 replace ubiqitin with ubiquitin
3. P21, l. 668 replace sequnece with sequence
4. Santt et al., 2008 appears two times citation 26 and 34
5. Figure 2 C CRACC -CT is supposed to be CRA-CTLH
6. Figure 1b: label the MKLN1-*UB band and crossreacting bands more precisely

Reviewer #2

(Remarks to the Author)
This study investigates substrate binding by the muskelin (MKLN1)-CTLH complex using cryo-EM. The authors have determined cryo-EM structures of the MKLN1-CTLH complex with its substrates MKLN1 itself, ZMYND19 and FAM72A-UNG2 complex. Their data reveals that 2 dimers of muskelin are recruited, one as part of the supramolecular complex, the other a substrate. They identify structural requirements for the self-association of muskelin and found that this assembly prevents association of other substrates, such a ZMYND19 and FAM72A that were tested in this study. Cryo-EM analysis of association of ZMYND19 and FAM72A show interactions with the discoidin and the Kelch domains of MKLN1 result in symmetrical and asymmetrical arrangements respectively. The authors identify and characterize distinct mutations that affect MKLN1 dimer recruitment and ZMYND19 and FAM72A recruitment. They found that loss of MKLN1 substrate binding promotes recruitment of alternate substrate (ZMYND19/FAM72) suggesting MKLN1 self assembly and ubiquitination mediates competes with other substrates’ modification. They also characterize MKLN1 as a Arg/C-degron receptor, although also functioning through an adaptor-mediated Ac/N-degron recognition mechanism.

This is a very elegant and complete study that reveals the structural details of substrate recruitment by the MKLN1-CTLH complex and provide evidence of distinct mechanisms explain how it recruits diverse substrates.

There are no major changes, only a few specific revisions are requested below.

1) None of the assays that lead to a quantitative statement or conclusion are actually quantified but rely on a visual assessment. In particular, cell assays need to be quantified, such as Fig 6b, e, f, k.

2) The effect of the N447R MKLN1 mutation has an effect in vitro on ZMYND19 ubiquitination, but there does not appear to have an effect in vivo (Fig 6e) as levels of ZMYND19 appear similar in WT and N447R-transfected cells in the inputs. Could the authors comment on this?

3) Also, there is no testing of UNG2 in these assays (done in cells as in Fig. 6e,f), confirming an effect on UNG2 would strengthen the model that is established in vitro. Notably, it could be done by hybridizing the blot shown in 6f.

4) line 68: reference 53 does not address GID4-dependent recruitment, this reference should be removed

5) line 335: the authors state that loss of “discoidin domain mutant paralleled those of the Kelch Loop-1 mutant …… increased alternative autoubiquitylation of other CTLH E3 subunits…” It appears only TWA1 ubiquitination is increased (in Suppl 4b), it is not clear which other CTLH subunit shows ubiquitination, this should be clarified.

6) why was the “N-terminally fluorescently-labeled “K0” ubiquitin with all lysines mutated to arginines” used to visualize direct substrate modification? (without additional banding from chain formation). It is not clear what the advantage is. Could preventing the formation of ubiquitin chains affect the overall result?

Reviewer #3

(Remarks to the Author)
The manuscript by Sepic et al discusses interactions of three distinct substrates (MKLN1, ZMYND19, and UNG2 via FAM72A) with the ligase CTLHMKLN1. It provides high-resolution insights into various modes of substrate recruitment, which is particularly valuable given the broad nature of the degrons recognized by this complex. The study also further supports several previous observations about MKLN1 tetramer structure and FAM72A-mediated UNG2 recruitment. Overall, the cryo-EM data are of high quality and convincing, and the resulting structural information are well supported by biochemical observations.
I recommend the manuscript for publication. I have several minor comments that could be addressed before publishing:

1- Competition between MKLN1 and other substrates
The manuscript often mentions how MKLN1 limits the binding of other substrates, such as ZMYND19 and UNG2, to the ligase. For example, lines 296–299 state: “Sub-MKLN1 binding is mutually exclusive with other substrates, and that neither ZMYND19 nor FAM72A engages the Kelch Loop-1 surface. The data indicate that MKLN1 self-recruitment limits CTLH^MKLN1 E3 access to other substrates competing for the discoidin domain trench.”)

While I agree that this is likely the case, it would be helpful to demonstrate this experimentally in one figure, either as a displacement assay with combinations of substrates, or by measuring the Kds of individual substrates to determine which would be the “preferred” substrate. It would also be useful to include a brief discussion of the cellular conditions under which CTLH^MKLN1 targets these substrates. For example, do these substrates compete under physiological conditions? Is one substrate present at a higher cellular concentration than the others? Could substrate selection be condition-dependent?

2- Structure of the manuscript

I found the order of the figures somewhat confusing. For example, when reading about binding of the MKLN1 homodimers, E3- and Sub-MKLN1, a natural question is whether only one type is ubiquitinated. This is addressed later in Fig. 6, but it would be more informative if this information appeared closer to the relevant structural data. A similar point applies to the other substrates.
Given the number of mutations analyzed, it would be helpful to include at least a schematic, if not the molecular contact details, next to the ubiquitination gels in which the individual mutants appear. This would make it easier to connect the structural observations with the biochemical assays.

3- representation of cryoEM maps
The representation of cryo-EM maps in the figures is inconsistent. I think docking surface views of models works well for the whole CTLH^MKLN1 complex, although, if the local-refinement cryo-EM structures are available, I think these would be more convincing. For the high-resolution local refinements, color-coded local-refinement maps, as shown in Fig. 5b for FAM72A-UNG2, for each of the three substrates and included in the main figures would be very helpful. There is also inconsistency in how molecular contacts are shown: some are shown with an overlay of the cryo-EM density, whereas others are not. All should be shown with the density. If the density is lacking in these regions, this should be clearly stated in the text. I don’t think this is the case though: the cryo-EM map are of high quality, and including them in overlays would only strengthen the manuscript.

>E3- and Sub-MKLN1 contacts , and ZMYND19 or FAM72-UNG2 contacts to MKLN1 – linear domain representation and contacts in between like in fig2c would make it easier to understand – a comparison in supplemental figure for example.

T>he introduction comments on the limitations of previous low-resolution CTLH^MKLN1 maps and substrate placement. However, some maps in the present study, particularly of the whole complex, are also at lower resolution. It may therefore be helpful to soften this language and frame the advance more neutrally, for example by emphasizing that the present work provides higher-resolution subcomplex structures that complement and extend previous studies.

>Fig. 1 gels: Could you label the additional bands as well, at least with E2/E1 labels, or show this in the supplementary figures somewhere as a reference to make all bands on the gel easy to understand?

>Fig. 2c: Could you fit the model into the map and perhaps show another view, such as a 90-degree rotation, to better visualize the dimerization of MKLN1?

>Substrate:MKLN1
Does your MKLN1 tetramer structure agree with the published structures? Can you comment on this in text?

In Fig. 3b, is the structure clipped? It appears that the full structure is not shown. Could the authors show the full structure and/or annotate that this is a clipped view or slice?

>Substrate:ZMYND19

Is there a known function for ZMYND19 dimerization? Are both protomers ubiquitinated? Could this be tested biochemically, or could the authors at least speculate based on the position of UBE2H~ubiquitin and the distance to each ZMYND19 protomer?

Line 217: Is it a more dynamic association, or is the binding more flexible? Also, decreasing the threshold of the class II map shows some density that looks like a second ZMYND19 bound.

What is the role of MKLN1 G273 in binding to ZMYND19? This residue is not described in the text as binding the C-terminus of ZMYND19-B, but it is used as a mutant in combination with W291A.

Fig. 2 and Supplementary Fig. 3 have the ZMYND19 A/B colors exchanged

Please state in text (main text ideally) that no important interfaces lie in separate local refinements.

>Substrate:UNG2
Has it been shown previously that acetylated UNG2 binds FAM72A? Could you expand on this in the text and cite relevant work, if applicable?
Does acetylation regulate only ubiquitination, or also binding? Is the N-terminus necessary for binding? Is it known whether any other regions of UNG2 interact with FAM72A?
Could you please make it clear in the text that the majority of UNG2 cannot be resolved in your cryo-EM maps?
Could you speculate on the function of W125 in FAM72A based on your structure? W125R is thought to be a mutation that no longer binds UNG2, despite not forming the interaction surface (Guo et al 2008 , Feng et al 2021, Rogier et al 2021 etc)

Version 1:

Reviewer comments:

Reviewer #1

(Remarks to the Author)
Dear Editor,

I have carefully reviewed the revised version of the manuscript and the authors’ responses to the previously raised comments and concerns.

The authors have addressed all points raised during the previous round of review and I consider the remaining issues to have been fully resolved.

In my opinion, the revised manuscript is now suitable for publication, and I recommend its acceptance in its present form.

Reviewer #2

(Remarks to the Author)
The authors have answered my comments satisfactorily.
I have no additional comments. The manuscript presents a complete and rigorous study that makes a valuable contribution to the field. I endorse it for publication.

Reviewer #3

(Remarks to the Author)
The manuscript by Sepic et al discusses interactions of three distinct substrates (MKLN1, ZMYND19, and UNG2 via FAM72A) with the ligase CTLHMKLN1. It provides high-resolution insights into various modes of substrate recruitment, which is particularly valuable given the broad nature of the degrons recognized by this complex. The study also further supports several previous observations about MKLN1 tetramer structure and FAM72A-mediated UNG2 recruitment. Overall, the cryo-EM data are of high quality and convincing, and the resulting structural information are well supported by biochemical observations.

All of my comments have been addressed and I recommend the manuscript for publication.

Open Access This Peer Review File is licensed under a Creative Commons Attribution 4.0 International License, which permits use, sharing, adaptation, distribution and reproduction in any medium or format, as long as you give appropriate credit to the original author(s) and the source, provide a link to the Creative Commons license, and indicate if changes were made.
In cases where reviewers are anonymous, credit should be given to 'Anonymous Referee' and the source.
The images or other third party material in this Peer Review File are included in the article's Creative Commons license, unless indicated otherwise in a credit line to the material. If material is not included in the article's Creative Commons license and your intended use is not permitted by statutory regulation or exceeds the permitted use, you will need to obtain permission directly from the copyright holder.
To view a copy of this license, visit https://creativecommons.org/licenses/by/4.0/

Multimodal substrate recruitment enables CTLH^{MKLN1} E3 ligase to target N-, C-, and internal degrons
Sara Sepic, Karthik V. Gottemukkala, Jiale Du, Samuel A. Maiwald, Jakub Chrustowicz, Eleftheria C. Papadopoulou, Luca J. Stier, Susanne von Gronau, Brenda A. Schulman

Point-by-Point Responses to Reviewer Comments (in blue)

Reviewer #1 (Remarks to the Author):

The manuscript by Sepic et al. presents an extensive structural and biochemical analysis of substrate recognition by the CTLHMKLN1 E3 ligase complex. Using cryo-EM, in vitro ubiquitylation assays, mutational analysis, and cellular experiments, the authors propose that MKLN1 employs combinatorial use of discoidin and Kelch β-propeller surfaces to recruit distinct substrate classes, including Arg/C-degrons, internal loop motifs, and adaptor-mediated Ac/N-degrons.

The study is technically strong and represents a significant advance for the field. The structural work is impressive, and the biochemical reconstitution is rigorous. However, several mechanistic conclusions currently extend beyond the available evidence, and additional clarification and moderation of claims are required before publication in Nature Communications.

Major points

1.The manuscript repeatedly refers to several motifs as “degrons,” although sufficiency for degradation has not been demonstrated. The authors should either provide reporter-based degron assays or moderate the terminology throughout.

We have added much new data to demonstrate sufficiency of the motifs for E3 ligase recognition.

C-terminal Arg degron:
In vitro binding: First, we now show, by fluorescence polarization assays, that fluorescein-labeled synthetic peptides corresponding to the 19 C-terminal residues of ZMYND19 and FAM72A are sufficient to bind the WT MKLN1 Kelch propeller (Fig. 6k of the revised manuscript). Importantly, the binding was substantially reduced for structure-based mutants. Second, the C-terminal Arg of full-length ZMYND19 is required for the protein to outcompete binding of the peptide. Notably, the WT ZMYND19 was able to outcompete the peptide, whereas the ZMYND19 lacking the C-terminal Arg was not (Supplementary Fig. 5d of the revised manuscript).

Structural data: During the time since manuscript submission, we also obtained a high-resolution experimental structure showing the last 4 residues of FAM72A binding to the MKLN1 Kelch domain pore (Fig. 6f). This complements our high-resolution experimental structure showing similar interactions for the ZMYND19 C-terminus. Thus, our paper provides the only experimental structural data showing this E3 recognition of the Arg C-terminal sequence, and we show that it is common for multiple MKLN1 partner proteins.

Cellular data: We added new cellular data showing that the ZMYND19 C-terminal deletion mutant is protected from proteasomal degradation compared to the WT protein (Fig. 6m and n of the revised manuscript).

UNG acetylated N-terminus:

In vitro experiments with purified proteins: First, we now added experiments showing that an N-terminally acetylated UNG2 peptide is sufficient to bind directly to MKLN1(N447R)-FAM72A ((Fig. 7g of the revised manuscript). The N447R MKLN1 mutant selectively prevents Sub-MKLN1 recruitment. Importantly, interactions were below the limit of detection of the assay for the unacetylated peptide. Second, full-length UNG2 that was N-terminally acetylated during production in insect cells could compete with the peptide for binding, but competition was substantially reduced for the unacetylated (bacterially produced) counterpart (Fig. 7h). Third, N-terminal acetylation is sufficient to substantially potentiate in vitro ubiquitylation of UNG2 (Fig. 7i). Finally, a natural control for the UNG2 N-terminus is UNG1, which differs from UNG2 only by its N-terminal sequence. UNG1 was not a substrate in vitro (Fig. 7i of the revised manuscript).

Cellular data: while the cellular levels of endogenous UNG2 increased upon expression of a MKLN1 structure-based mutant, UNG1 was unaffected (Fig. 6i and 6j).

Overall, our study shows that the motifs are sufficient and required to bind to MKLN1, and that they are necessary for ubiquitylation and degradation. Thus, we have modified our terminology to indicate that we define the recognition motifs. Contemporaneous, complementary work posted on bioRxiv while our manuscript was in preparation shows sufficiency for degradation, but lack the detailed mechanistic analyses. We cite those studies throughout our manuscript. Importantly, we uniquely experimentally - and comprehensively - reveal the structural bases for these interactions. Thus, based on our study together with the work of others, we feel comfortable with statements that our study reveals the structural basis for degron recognition.

2.The proposed competition between MKLN1 self-recruitment and recruitment of other substrates is attractive but remains indirect. This model could be tested more directly by quantitative competition assays in which ZMYND19 or FAM72A–UNG2 are titrated against MKLN1 autoubiquitylation, or by reciprocal experiments testing whether overexpression of MKLN1 suppresses ZMYND19/UNG2 ubiquitylation and degradation.

We performed the experiments examining competitive ubiquitylation. The new data are shown in Fig. 7j-l of the revised manuscript. Specifically, we have added titration experiments clearly showing that ZMYND19 competes with Sub-MKLN1, and that FAM72A/UNG2 competes with Sub-MKLN1. We also provide evidence that ZMYND19 competes with FAM72A which is not itself a substrate by showing that ZMYND19 suppresses autoubiquitylation across the $CTLH^{MKLN1}$-FAM72A complex. (We note that these experiments were performed with lysineless, aka K0, ubiquitin to visualize the direct substrates rather than polyubiquitylation).

3. Cellular validation is comparatively limited relative to the structural claims. Additional degradation or protein half-life analyses would strengthen the physiological relevance of the proposed interfaces.

We have addressed this by examining effects of cellular treatment with MG132 to prevent proteasomal degradation. First, we examined Torin1-dependent degradation of MKLN1. Co-treatment with MG132 and Torin1 rescued the levels of WT MKLN1 to those of the untreated condition. Similar results were obtained for the control Kelch domain mutant (G273W/W291G that does not impact Sub-MKLN1 interaction or in vitro ubiquitylation). However, the N447R mutant MKLN1, which selectively abolishes the interaction with Sub-MKLN1 and whose levels are not reduced by Torin1, is not affected by co-treatment with MG132 (Fig 6d-e). Second, we now show that MG132 also impairs C-terminal Arg-dependent degradation of ZMYND19 (Fig. 6m and n). Third, similar results were obtained for UNG2 (Fig. 6i and j). Briefly, we analyzed UNG2 degradation in MKLN1 KO cells upon ectopic expression of MKLN1 and FAM72A (for which endogenous is not detectable).

MG132 increased the level of UNG2 only for WT MKLN1 and the N447R mutant (control, which affects Sub-MKLN1 but not FAM72A binding in vitro). However, the effect of MG132 was not observed for the MKLN1 mutant (W291A, G273W) impaired for binding to the FAM72A C-terminal Arg (Fig. 6i-j of the revised manuscript).

Minor points

1. Terminology should be standardized throughout (e.g., CTLH^MKLN1 versus CTLHMKLN1).
We have standardized the notation to $CTLH^{MKLN1}$ (superscript) throughout the revised manuscript.

2. P18, l. 567, 578, 579 replace ubiqitin with ubiquitin
We thank the reviewer for spotting the typos. We have corrected them in the revised manuscript.

3. P21, l. 668 replace sequnece with sequence
We thank the reviewer for spotting the typo. We have corrected this in the revised manuscript.

4. Santt et al., 2008 appears two times citation 26 and 34
We thank the reviewer for spotting this. We have removed the duplicate citation and renumbered accordingly.

5. Figure 2 C CRACC -CT is supposed to be CRA-CTLH
We have corrected this label in the revised Figure 2C.

6. Figure 1b: label the MKLN1-*UB band and crossreacting bands more precisely
We updated Figure 1 accordingly.

Reviewer #2 (Remarks to the Author):

This study investigates substrate binding by the muskelin (MKLN1)-CTLH complex using cryo-EM. The authors have determined cryo-EM structures of the MKLN1-CTLH complex with its substrates MKLN1 itself, ZMYND19 and FAM72A-UNG2 complex. Their data reveals that 2 dimers of muskelin are recruited, one as part of the supramolecular complex, the other a substrate. They identify structural requirements for the self-association of muskelin and found that this assembly prevents association of other substrates, such a ZMYND19 and FAM72A that were tested in this study. Cryo-EM analysis of association of ZMYND19 and FAM72A show interactions with the discoidin and the Kelch domains of MKLN1 result in symmetrical and asymmetrical arrangements respectively. The authors identify and characterize distinct mutations that affect MKLN1 dimer recruitment and ZMYND19 and FAM72A recruitment. They found that loss of MKLN1 substrate binding promotes recruitment of alternate substrate (ZMYND19/FAM72) suggesting MKLN1 self assembly and ubiquitination mediates competes with other substrates' modification. They also characterize MKLN1 as a Arg/C-degron receptor, although also functioning through an adaptor-mediated Ac/N-degron recognition mechanism.

This is a very elegant and complete study that reveals the structural details of substrate recruitment by the MKLN1-CTLH complex and provide evidence of distinct mechanisms explain how it recruits diverse substrates.

There are no major changes, only a few specific revisions are requested below.

1) None of the assays that lead to a quantitative statement or conclusion are actually quantified but rely on a visual assessment. In particular, cell assays need to be quantified, such as Fig 6b, e, f, k.

We provide the quantification for these immunoblots, as well as Fig. 6d and 6i, 6m to address suggestions from Reviewer #1.

2) The effect of the N447R MKLN1 mutation has an effect in vitro on ZMYND19 ubiquitination, but there does not appear to have an effect in vivo (Fig 6e) as levels of ZMYND19 appear similar in WT and N447R-transfected cells in the inputs. Could the authors comment on this?

In the cellular assays ZMYND19 and MKLN1 are co-expressed. We speculate that ZMYND19 dominates over Sub-MKLN1 under these circumstances, especially because ZMYND19 outcompetes Sub-MKLN1 in our titration experiments (Fig. 7j of the revised manuscript). We now address this in the revised Discussion.

3) Also, there is no testing of UNG2 in these assays (done in cells as in Fig. 6e,f), confirming an effect on UNG2 would strengthen the model that is established in vitro. Notably, it could be done by hybridizing the blot shown in 6f.

We performed this experiment now shown in Fig. 6i and j of the revised manuscript.

4) line 68: reference 53 does not address GID4-dependent recruitment, this reference should be removed.

We have removed the citation and renumbered accordingly.

5) line 335: the authors state that loss of “discoidin domain mutant paralleled those of the Kelch Loop-1 mutant ……increased alternative autoubiquitylation of other CTLH E3 subunits…” It appears only TWA1 ubiquitination is increased (in Suppl 4b), it is not clear which other CTLH subunit shows ubiquitination, this should be clarified.

The Reviewer is correct that the increased ubiquitylation is most clearly observed for TWA1. We have revised the text accordingly to read: *"…paralleled those of the Kelch Loop-1 mutant..., with increased autoubiquitylation of the TWA1 subunit..."*

6) why was the “N-terminally fluorescently-labeled “K0” ubiquitin with all lysines mutated to arginines” used to visualize direct substrate modification? (without additional banding from chain formation). It is not clear what the advantage is. Could preventing the formation of ubiquitin chains affect the overall result?

Use of lysineless K0 ubiquitin allows distinguishing substrates in the same reaction based on unambiguous banding patterns. The banding patterns become too complicated to do this upon polyubiquitylation (including in the background of multi-monoubiquitylation) where MKLN1, multiple CTLH subunits (in the absence of robust substrate ubiquitylation), and in some experiments multiple substrates are all contributing to multiple bands.

Reviewer #3 (Remarks to the Author):

The manuscript by Sepic et al discusses interactions of three distinct substrates (MKLN1, ZMYND19, and UNG2 via FAM72A) with the ligase CTLHMKLN1. It provides high-resolution insights into various modes of substrate recruitment, which is particularly valuable given the broad nature of the degrons recognized by this complex. The study also further supports several previous observations about MKLN1 tetramer structure and FAM72A-mediated

UNG2 recruitment. Overall, the cryo-EM data are of high quality and convincing, and the resulting structural information are well supported by biochemical observations.
I recommend the manuscript for publication. I have several minor comments that could be addressed before publishing:

1- Competition between MKLN1 and other substrates
The manuscript often mentions how MKLN1 limits the binding of other substrates, such as ZMYND19 and UNG2, to the ligase. For example, lines 296–299 state: "Sub-MKLN1 binding is mutually exclusive with other substrates, and that neither ZMYND19 nor FAM72A engages the Kelch Loop-1 surface. The data indicate that MKLN1 self-recruitment limits CTLH^MKLN1 E3 access to other substrates competing for the discoidin domain trench.") While I agree that this is likely the case, it would be helpful to demonstrate this experimentally in one figure, either as a displacement assay with combinations of substrates, or by measuring the Kds of individual substrates to determine which would be the "preferred" substrate. It would also be useful to include a brief discussion of the cellular conditions under which CTLH^MKLN1 targets these substrates. For example, do these substrates compete under physiological conditions? Is one substrate present at a higher cellular concentration than the others? Could substrate selection be condition-dependent?

We performed the experiments examining competitive ubiquitylation. The new data are shown in Fig. 7j-l of the revised manuscript. Specifically, we have added titration experiments clearly showing that ZMYND19 competes with Sub-MKLN1, and that FAM72A/UNG2 competes with Sub-MKLN1. We also provide evidence that ZMYND19 competes with FAM72A which is not itself a substrate by showing that ZMYND19 suppresses autoubiquitylation across the $CTLH^{MKLN1}$-FAM72A complex. (We note that these experiments were performed with lysineless, aka K0, ubiquitin to visualize the direct substrates rather than polyubiquitylation).

We also expanded the Discussion to consider that substrate specificity may be context dependent. For example, ZMYND19 and MKLN1 degradation are stimulated by inhibition of mTORC1 signaling pathway, whereas FAM72A expression is highly cell-type specific and enriched in B cells. Thus, cellular substrate availability and physiological state likely influence $CTLH^{MKLN1}$ substrate selection.

2- Structure of the manuscript
I found the order of the figures somewhat confusing. For example, when reading about binding of the MKLN1 homodimers, E3- and Sub-MKLN1, a natural question is whether only one type is ubiquitinated. This is addressed later in Fig. 6, but it would be more informative if this information appeared closer to the relevant structural data. A similar point applies to the other substrates.
Given the number of mutations analyzed, it would be helpful to include at least a schematic, if not the molecular contact details, next to the ubiquitination gels in which the individual mutants appear. This would make it easier to connect the structural observations with the biochemical assays.

We have added structural cartoons to Fig. 2 showing the overall schematic, and to Figs. 6, and 7 to highlight the locations of the mutations.

3- representation of cryoEM maps
The representation of cryo-EM maps in the figures is inconsistent. I think docking surface views of models works well for the whole CTLH^MKLN1 complex, although, if the local-refinement cryo-EM structures are available, I think these would be more convincing. For the high-resolution local refinements, color-coded local-refinement maps, as shown in Fig. 5b for FAM72A-UNG2, for each of the three substrates and included in the main figures would be very helpful. There is also inconsistency in how molecular contacts are shown: some are

shown with an overlay of the cryo-EM density, whereas others are not. All should be shown with the density. If the density is lacking in these regions, this should be clearly stated in the text. I don't think this is the case though: the cryo-EM map are of high quality, and including them in overlays would only strengthen the manuscript.

The ZMYND19 subcomplex structure is built from several local refinements, which are superior in quality compared to the overall composite map. Because the composite map encompasses the complete subcomplex assembly, we prefer to show this map transparently superimposed with the structures in the main figures. However, to address the Reviewer, we also now show all three color-coded individual local refined maps side-by-side showing discoidin domain interactions in Supp. Fig. 6. We now also show the cryo-EM density in all the figures with molecular contacts.

Additional points

>E3- and Sub-MKLN1 contacts, and ZMYND19 or FAM72-UNG2 contacts to MKLN1 – linear domain representation and contacts in between like in fig2c would make it easier to understand – a comparison in supplemental figure for example.

We now expanded Fig. 2 to provide cartoons of the full complexes. We also show cartoons of subcomplexes between E3-MKLN1 and the substrates in Figures 6 and 7. The new cartoons provide domain representation and contacts. We are showing these as structural representations rather than linear representation because of the complexities of the assemblies.

>The introduction comments on the limitations of previous low-resolution CTLH^MKLN1 maps and substrate placement. However, some maps in the present study, particularly of the whole complex, are also at lower resolution. It may therefore be helpful to soften this language and frame the advance more neutrally, for example by emphasizing that the present work provides higher-resolution subcomplex structures that complement and extend previous studies.

We have softened the language accordingly and now state: "These cryo-EM maps and focus-refined maps revealing molecular details of subcomplexes, taken together with biochemical and cell-based data herein and from previous and recent studies[53,66,67], reveal a diversity of mechanisms for $CTLH^{MKLN1}$ placement of various substrates for ubiquitylation."

>Fig. 1 gels: Could you label the additional bands as well, at least with E2/E1 labels, or show this in the supplementary figures somewhere as a reference to make all bands on the gel easy to understand?

We have updated Fig. 1, and added Supp. Fig. 1 which is an annotated Coomassie-stained gel (corresponding to Fig. 1d) with labeled bands.

>Fig. 2c: Could you fit the model into the map and perhaps show another view, such as a 90-degree rotation, to better visualize the dimerization of MKLN1?

We apologize for any confusion, but Fig. 2c (Fig. 2d in the revised manuscript) shows a model derived from the $CTLH^{MKLN1}$ map and our high-resolution structure of E3-MKLN1-A bound to Sub-MKLN1 (PDB: 29UE). We have now clarified this in the figure legend. In addition, to provide greater understanding of interactions, we have now added cartoons.

>Substrate:MKLN1

Does your MKLN1 tetramer structure agree with the published structures? Can you comment on this in text?

We apologize for any confusion in our original text. That was unintentional. We replaced the original statement with: “Such arrangements had been observed in prior cryo-EM maps of MKLN1 alone (EMD-45088, EMD-41612)[44] and in subcomplexes (EMD-12547)[,57], although this arrangement had not been previously observed in the context of a CTLH E3 complex. “

In Fig. 3b, is the structure clipped? It appears that the full structure is not shown. Could the authors show the full structure and/or annotate that this is a clipped view or slice?

We have now updated the Fig. 3b legend as follows: Map of E3-MKLN1–Sub-MKLN1 tetrameric assembly shown over model of E3-MKLN1 discoidin domains engaging the Sub-MKLN1-A Kelch β-propeller fitted from E3-MKLN1-A–Sub-MKLN1-A high-resolution structure (PDB: 29UE).

>Substrate: ZMYND19
Is there a known function for ZMYND19 dimerization? Are both protomers ubiquitinated? Could this be tested biochemically, or could the authors at least speculate based on the position of UBE2H~ubiquitin and the distance to each ZMYND19 protomer?

In our 3D classification, the ZMYND domain of one ZMYND19 protomer is less resolved, consistent with the asymmetric binding mode observed in our structures. However, analysis of the various 3D classes indicates that both protomers are positioned within reach of the UBE2H~ubiquitin catalytic machinery, suggesting that both are capable of being ubiquitylated.

Line 217: Is it a more dynamic association, or is the binding more flexible? Also, decreasing the threshold of the class II map shows some density that looks like a second ZMYND19 bound.

We apologize for the lack of clarity. We changed the sentence to: “Third, different 3D classes showed varying extents of density for ZMYND19. The entirety of one molecule (hereafter termed ZMYND19-A) is visible in all classes, along with the C-terminus of the second (hereafter termed ZMYND19-B) (Fig. 4a). A subset of classes also show density across the full ZMYND19-B protomer, albeit at low contour and at relatively low resolution (Supplementary Fig. 4a).”

What is the role of MKLN1 G273 in binding to ZMYND19? This residue is not described in the text as binding the C-terminus of ZMYND19-B, but it is used as a mutant in combination with W291A.

We apologize for the confusion. We have now re-labeled our figures to indicate the position of G273. G273 and W291 sandwich the C-terminal Arg. The W291 aromatic ring stacks against the Arg side chain, thus the Ala substitution would eliminate this interaction. Meanwhile, introducing a bulky Trp at the position of G273 would sterically block the substrate's C-terminus. We have added this information to the description of the structure, and to the section on mutational analysis.

Fig. 2 and Supplementary Fig. 3 have the ZMYND19 A/B colors exchanged

We have corrected the color assignments for ZMYND19 A/B in both Fig. 2 and Supplementary Fig. 3 of the revised manuscript.

Please state in text (main text ideally) that no important interfaces lie in separate local refinements.

We have added a sentence in the main text stating: "In each case, the side-chains described in interactions below were resolved within a single local refinement, ensuring that the relative positioning of interacting residues was directly supported by the cryo-EM density rather than inferred from merging separately refined regions".

>Substrate: UNG2
Has it been shown previously that acetylated UNG2 binds FAM72A? Could you expand on this in the text and cite relevant work, if applicable?

Although the interaction has been mapped to the N-terminus of UNG2, the role of N-terminal acetylation was neither suggested nor tested previously. To our knowledge, our paper is the first to report that N-terminal acetylation of UNG2 mediates its interaction with an E3 ligase.

Does acetylation regulate only ubiquitination, or also binding? Is the N-terminus necessary for binding? Is it known whether any other regions of UNG2 interact with FAM72A?

We now added experiments showing that an N-terminally acetylated UNG2 peptide is sufficient to bind directly to MKLN1(N447R)-FAM72A (Fig. 7g of the revised manuscript). Here, the N447R mutant version of MKLN1 was used to prevent Sub-MKLN1 recruitment. Importantly, interactions were below the limit of detection of the assay for the unacetylated peptide. Also, full-length UNG2 that was N-terminally acetylated during production in insect cells could compete with the peptide for binding, but competition was substantially reduced for the unacetylated (bacterially produced) counterpart (Fig. 7h). Moreover, N-terminal acetylation is sufficient to substantially potentiate in vitro ubiquitylation of UNG2 (Fig. 7i). Finally, a natural control for the UNG2 N-terminus is UNG1, which differs from UNG2 only by its N-terminal sequence. UNG1 was not a substrate in vitro (Fig. 7i of the revised manuscript).

Could you please make it clear in the text that the majority of UNG2 cannot be resolved in your cryo-EM maps?

We have now added a sentence: "…we obtained cryo-EM data for complexes representing ubiquitylation of the three substrates, MKLN1, ZMYND19, and FAM72A-UNG2. Ultimately, we visualized only a short peptide sequence from UNG2 bound to MKLN1-FAM72A, suggesting that the remainder of this substrate is flexibly tethered to the E3."

Could you speculate on the function of W125 in FAM72A based on your structure? W125R is thought to be a mutation that no longer binds UNG2, despite not forming the interaction surface (Guo et al 2008, Feng et al 2021, Rogier et al 2021 etc)

Indeed, a W125R mutation used in these studies prevented binding to UNG2. Our high-resolution MKLN1-FAM72A-UNG2 structure shows this Trp125 does not directly bind UNG2. Instead, it is central to the fold of a structural region adjacent to the UNG2 binding site, where it organizes seven side-chains from five different secondary structure elements. An Arg substitution, if tolerated, would at minimum cause substantial structural rearrangements, if not misfolding of a region whose effects would reverberate to the substrate-binding pocket.

RESPONSE TO REVIEWERS' COMMENTS

Reviewer #1 (Remarks to the Author):

Dear Editor,

I have carefully reviewed the revised version of the manuscript and the authors' responses to the previously raised comments and concerns.

The authors have addressed all points raised during the previous round of review and I consider the remaining issues to have been fully resolved.

In my opinion, the revised manuscript is now suitable for publication, and I recommend its acceptance in its present form.

We are very pleased by the review of our revision and the recommendation to publish our manuscript!

Reviewer #2 (Remarks to the Author):

The authors have answered my comments satisfactorily.
I have no additional comments. The manuscript presents a complete and rigorous study that makes a valuable contribution to the field. I endorse it for publication.

We are very pleased that the Reviewer finds our work to be a valuable contribution to the field, and by the recommendation to publish our manuscript!

Reviewer #3 (Remarks to the Author):

The manuscript by Sepic et al discusses interactions of three distinct substrates (MKLN1, ZMYND19, and UNG2 via FAM72A) with the ligase CTLHMKLN1. It

provides high-resolution insights into various modes of substrate recruitment, which is particularly valuable given the broad nature of the degrons recognized by this complex. The study also further supports several previous observations about MKLN1 tetramer structure and FAM72A-mediated UNG2 recruitment. Overall, the cryo-EM data are of high quality and convincing, and the resulting structural information are well supported by biochemical observations.

All of my comments have been addressed and I recommend the manuscript for publication.

We are very pleased that the Reviewer finds our work valuable, and by the recommendation to publish our manuscript!
